# A General Solution for the 2-Pyridyl Problem**

**DOI:** 10.1002/anie.201108608

**Published:** 2012-01-27

**Authors:** Graham R Dick, Eric M Woerly, Martin D Burke

**Affiliations:** Howard Hughes Medical Institute, Department of Chemistry, University of Illinois at Urbana-Champaign600 S. Mathews Ave, Urbana, IL 61801 (USA) E-mail: burke@scs.uiuc.edu Homepage: http://www.scs.illinois.edu/burke

**Keywords:** boronates, copper, cross-coupling, heterocycles, synthetic methods

Most small molecules are highly modular in their constitution, which suggests a potential general capacity for simple, efficient, and flexible construction through iterative coupling of preassembled building blocks.^1^, ^2^ In an idealized form of this approach, stable subunits representing the most common substructural elements found in a wide range of small-molecule targets are readily linked together using only cross-coupling reactions.^1^ In this vein, the 2-pyridyl subunit is one of the most prevalent and therefore important motifs, being found in a wide range of pharmaceuticals,^3^ natural products,^4^ unnatural nucleotides,^5^ fluorescent probes,^6^ materials,^7^ and metal-complexing ligands^8^ (Figure 1). Tremendous effort over the past three decades has been dedicated to the development of 2-pyridyl organometallic reagents that can be efficiently employed in cross-coupling reactions.^9^–^16^ All of these methods, however, suffer from one or more important limitations, including lack of air stability of the 2-pyridyl building blocks,^10^, ^12^–^16^ use of toxic metals,^9^ inability to isolate the building blocks in chemically pure form,^11^ and inefficient couplings with more challenging halide coupling partners such as deactivated aryl chlorides.^11^, ^12^, 13b–17 Overcoming all of these limitations, we herein report the first general solution for the 2-pyridyl problem.

**Figure 1:**
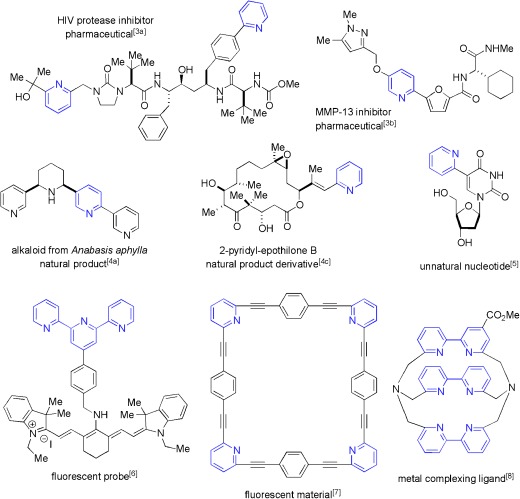
The 2-pyridyl motif is found in many important small molecules.

Typically, the 2-pyridyl–boron bond is exquisitely sensitive to protodeborylation, making most 2-pyridyl boranes unstable. In contrast, we recently identified 2-pyridyl *N*-methyliminodiacetic acid (MIDA) boronate (**1 a**; Scheme 1 a) as the first 2-pyridyl borane that is both air stable and can be isolated in a chemically pure form.^17^ We also developed an inexpensive, environmentally friendly, and scalable method for preparing **1 a** and many of its derivatives (Scheme 1 b).^18^ Importantly, all of these new building blocks are monomeric, highly crystalline, free-flowing solids that can be stored indefinitely on the bench top in air without decomposition, and several are now commercially available.^19^

**Scheme 1:**
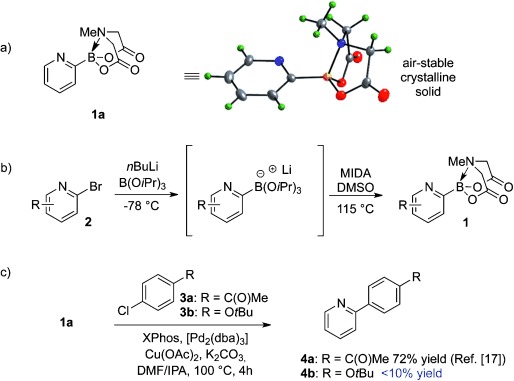
a) 2-pyridyl MIDA boronate **1 a** is the first air-stable 2-pyridyl borane that can be isolated in chemically pure form. b) An inexpensive, environmentally friendly, and scalable method for preparing 2-pyridyl MIDA boronates. c) A preliminary method for cross-coupling **1 a** with activated aryl chlorides. This method is ineffective with more challenging deactivated aryl halides. dba=dibenzylideneacetone, DMF=*N*,*N*′-dimethylfomamide, DMSO=dimethyl sulfoxide, IPA=isopropyl alcohol.

Having achieved all of these long sought after features in a collection of 2-pyridyl boranes, finding maximally general conditions to promote the cross-coupling of these building blocks became the final key goal. Kinetically competitive in situ decomposition usually hinders the effective cross-coupling of 2-pyridyl boranes. Because electronically and sterically deactivated aryl halides tend to react more slowly than their activated counterparts, they are especially challenging coupling partners.^20^ To overcome similar challenges with other sensitive 2-heterocyclic boronic acids, we introduced the slow-release cross-coupling strategy.^17^ Specifically, in the presence of mild bases and water as a cosolvent, air-stable MIDA boronates undergo in situ hydrolysis to liberate the corresponding boronic acids at a rate that is slower than catalyst turnover.^17^ Analogous to utilizing a syringe pump, such conditions strongly favor cross-coupling over boronic acid decomposition.^17^, ^21^

Presumably as a result of the extreme lability of the 2-pyridyl–boron bond, even under these slow-release conditions, the cross-coupling of 2-pyridyl MIDA boronate remained challenging. As shown in Scheme 1 c, modified reaction conditions employing isopropyl alcohol instead of water as a cosolvent and Cu(OAc)_2_ as a substoichiometric additive were somewhat effective with activated, electron-deficient aryl chlorides such as **3 a**. However, when we attempted to cross-couple **1 a** with more challenging deactivated aryl chlorides, such as **3 b**, very little of the desired cross-coupling product **4 b** was observed.

An extensive survey of palladium/ligand combinations,^22^ copper salts,^14^, ^16^, ^17^, ^23^ bases, solvents, temperatures, and reaction times resulted in reaction conditions that were somewhat more effective, but the yield of **4 b** remained modest (Table 1, entry 1). Driven by our then working hypothesis that the role of IPA in these reactions was to promote initial transligation of **1 a** to the corresponding 2-pyridyl isopropyl boronic ester, we investigated a range of different alcohols as additives. However, less (entries 2 and 3) or more (entry 4) sterically bulky alcohols were all inferior to IPA, and common diols also provided no notable advantage (entries 5–7). In contrast, addition of the trivalent ligand diethanolamine (DEA) resulted in the intriguing formation of a royal-blue reaction mixture and the formation of **4 b** in a substantially increased yield of 70 % (entry 8).

**Table 1:** Cross-coupling of 2-pyridyl MIDA boronate 1 a with deactivated aryl chloride 3 b^[a]^
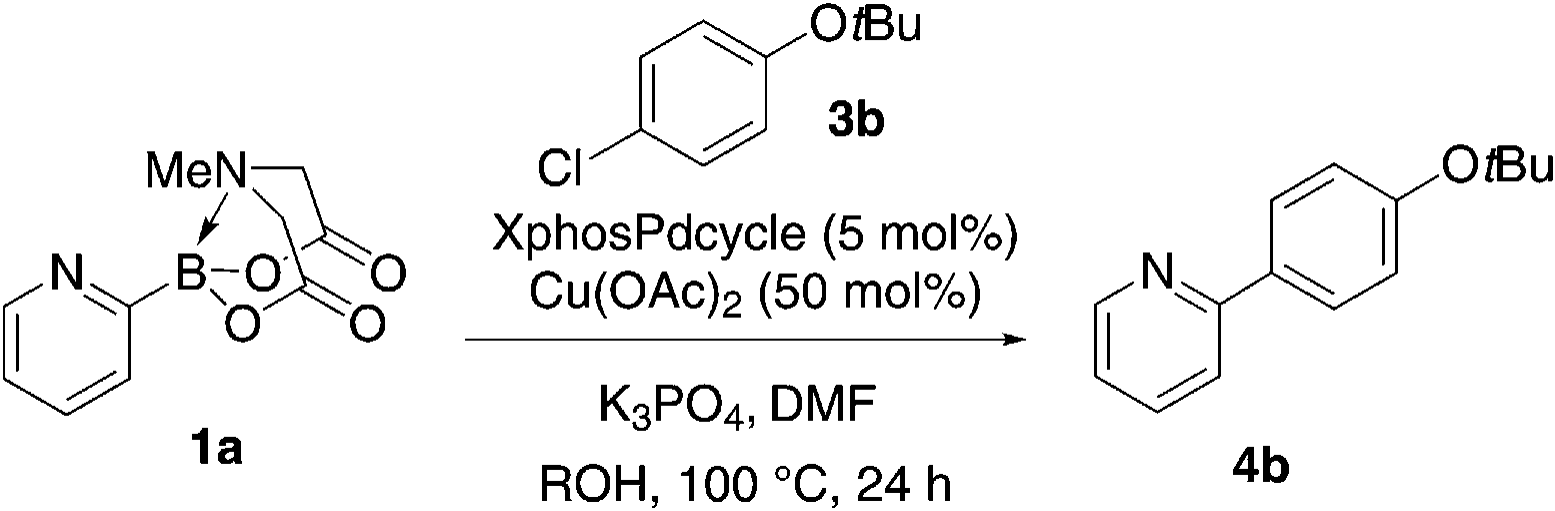

Entry	ROH	Equiv	Yield [%]^[b]^
1	IPA	3	49
2	MeOH	3	36
3	EtOH	3	39
4	*t*BuOH	3	43
5	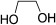	1.5	51
6	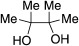	1.5	35
7	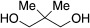	1.5	31
8	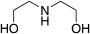	1.5	70
	DEA		

[a] Reaction conditions: 1.0 equiv **3 b** (1.0 mmol), 1.5 equiv **1 a**, 5 mol % XphosPdcycle, 50 mol % Cu(OAc)_2_, 5 equiv K_3_PO_4_, 0.125 m in DMF. [b] Determined by GC analysis. XPhosPdcycle=chloro(2-dicyclohexylphosphino-2′,4′,6′-triisopropyl-1,1′-biphenyl)[2-(2-aminoethyl)phenyl]-palladium(II) methyl *tert*-butyl ether adduct.

To enable further optimization of these reaction conditions, we sought to understand the mechanistic underpinnings of this DEA-promoted increase in efficiency. Deng and co-workers have shown that the cross-coupling of 2-pyridyl boronic esters promoted by copper(I) salts likely involves an initial C–B to C–Cu transmetalation to produce an intermediate 2-pyridyl copper species which, in turn, undergoes transmetalation with palladium(II).14a Starting with this general mechanistic framework, we considered two possible pathways for DEA to promote the transformation of **1 a** into a the putative 2-pyridyl copper intermediate **6** (Scheme 2). In pathway 1, DEA reacts with the conformationally rigid **1 a** in a novel transligation reaction to form a conformationally flexible and thereby more reactive DEA adduct **5**,1d which in turn transmetalates with Cu(OAc)_2_ to form **6**. In pathway 2, DEA alternatively reacts with Cu(OAc)_2_ to yield a Cu(DEA)_*n*_ complex^24^, ^25^ and KOAc. The released KOAc then reacts with **1 a** to generate a reactive 2-pyridyl boronate intermediate **7** (X=acetate^26^ or other ion), which in turn transmetalates with Cu(DEA)_*n*_ to form **6**.

**Scheme 2:**
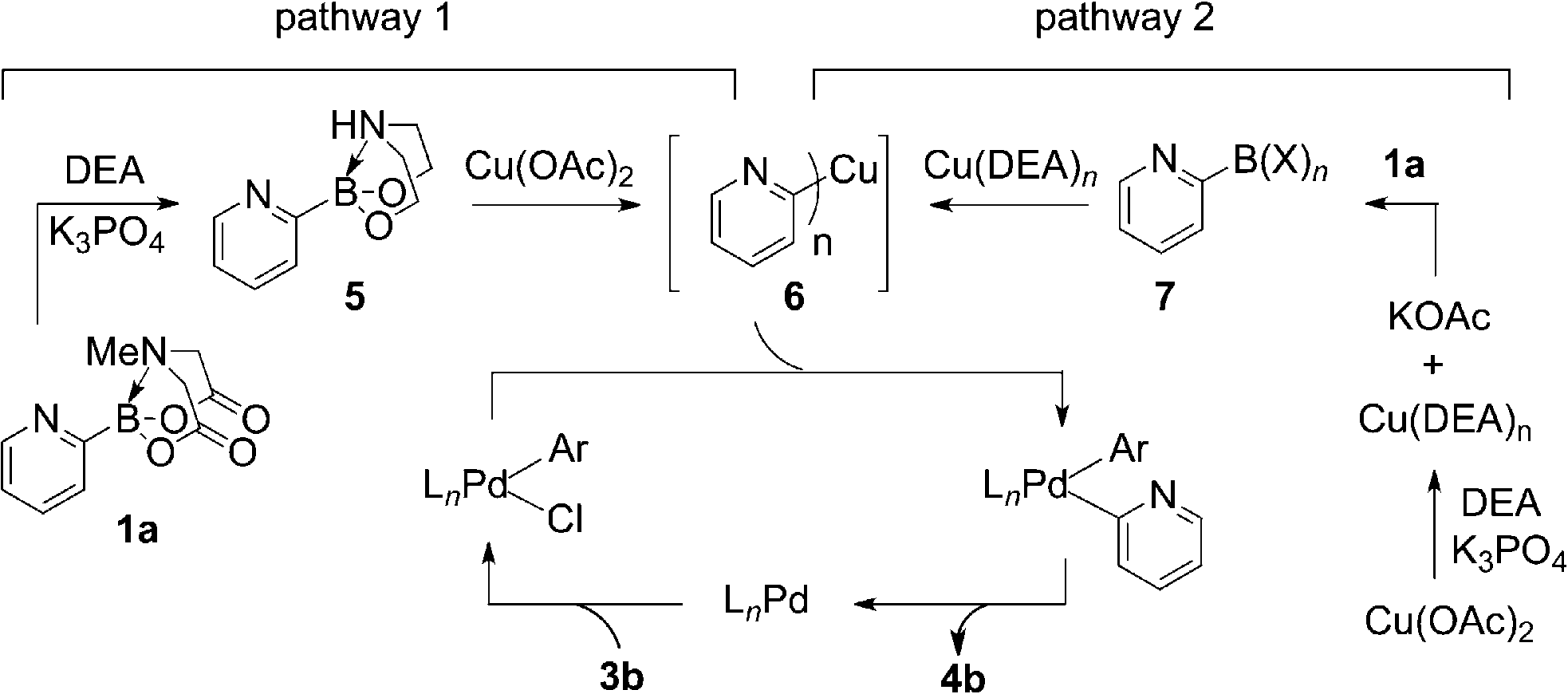
Two possible pathways for the DEA-promoted coupling of **1 a**.

To determine whether pathway 1 was operative, we first mixed DEA with **1 a** in the presence of K_3_PO_4_ in deuterated DMF at 100 °C and monitored the reaction by ^1^H NMR spectroscopy. Seeming to support this mechanism, we observed the slow transligation of **1 a** to **5** over the course of four hours (see the Supporting Information) and succeeded in isolating **5** as a crystalline solid (Scheme 3). However, when we attempted to couple to **5** to **3 b** with or without syringe-pump-mediated slow addition of **5** over the course of four hours to mimic the rate of its in situ formation,^17^ we observed only very low yields of **4 b** (Scheme 3). Thus, pathway 1 cannot account for the beneficial effects of DEA on the coupling of **1 a** and **3 b**.

**Scheme 3:**
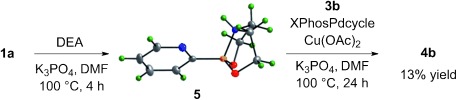


To interrogate the possibility of pathway 2, we alternatively combined DEA with Cu(OAc)_2_ in the presence of K_3_PO_4_ in DMF at 100 °C (Scheme 4). In less than 15 minutes the reaction turned royal blue, and both Cu(DEA)_2_ and KOAc were formed. After extensive experimentation, we developed a new procedure for preparing and purifying Cu(DEA)_2_ from CuCl_2_, DEA, and K_3_PO_4_ (see the Supporting Information).^27^ Strikingly, when we attempted to couple **1 a** and **3 b** in the presence of purified Cu(DEA)_2_ and KOAc under our otherwise standard conditions we observed an 84 % yield of **4 b** (Scheme 5; see entry 1). Consistent with important roles for both of these additives, in the absence of Cu(DEA)_2_, without or with added KOAc, none of this cross-coupling product was observed (Scheme 5; see entries 2 and 3). The Cu(DEA)_2_ was superior to Cu(OAc)_2_ (Scheme 5; see entry 4), and the addition of Cu(DEA)_2_ but not KOAc provided only a modest yield of **4 b** (entry 5).^28^

**Scheme 4:**
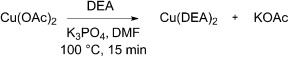


**Scheme 5:**
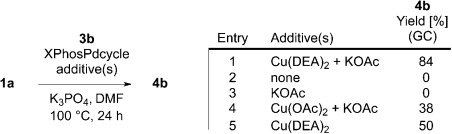


To further probe the role of KOAc in this reaction, we treated **1 a** with K_3_PO_4_ in DMF at 100 °C with or without adding KOAc and monitored the consumption of **1 a** by ^1^H NMR spectroscopy. In both the absence and presence of Cu(DEA)_2_, the addition of KOAc to otherwise identical reaction conditions resulted in substantially accelerated conversion of **1 a** into pyridine, presumably through protodemetalation of the short-lived intermediates **7** or **6**. Although additional studies will be needed to characterize this mechanism in further detail, all of this data is consistent with pathway 2 (Scheme 2).

Importantly, this mechanism also proved to be predictive for further optimizing this cross-coupling system. Specifically, the intermediacy of Cu(DEA)_2_ predicts that the optimum ratio of DEA/Cu(OAc)_2_ would be 2:1. We tested this hypothesis systematically and found that in fact a 2:1 ratio provided the highest yield (GC) of **4 b** (Scheme 6). By employing this rationally optimized ratio of additives on a 1 mmol scale, we obtained a 94 % yield upon product isolation for this very challenging cross-coupling reaction.

**Scheme 6:**
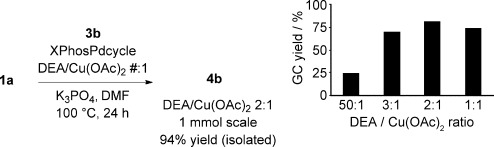


With this optimized methodology in hand, we explored its scope with respect to both the 2-pyridyl MIDA boronate and halide coupling partners. Remarkably, the same set of reaction conditions proved to be highly general. For example, as shown in Table 2, a series of electron-rich and sterically bulky aryl chlorides were coupled with **1 a** in typically good to excellent yields (entries 1–5). Even the highly deactivated 2,6-dimethoxy chlorobenzene (**3 h**) was coupled in synthetically useful yield (entry 6). Importantly, the same reaction conditions optimized for deactivated substrates were also effective for coupling **1 a** with electronically activated aryl chlorides (entries 7–9), and a diverse series of heteroaryl chlorides (entries 10–14).

**Table 2:** General cross-coupling of air-stable 2-pyridyl MIDA boronate 1 a with aryl and heteroaryl chlorides 3^[a]^
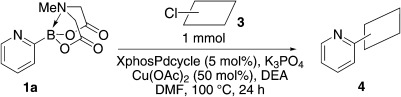

Entry	3		4		Yield [%]^[b]^
1	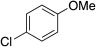	**3 c**	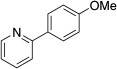	**4 c**	76
2	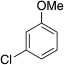	**3 d**	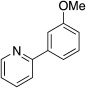	**4 d**	96
3	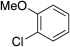	**3 e**	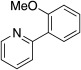	**4 e**	75
4	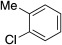	**3 f**	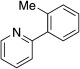	**4 f**	79
5	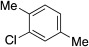	**3 g**	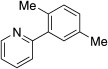	**4 g**	72
6^[c]^	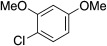	**3 h**	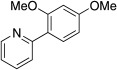	**4 h**	49
7	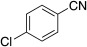	**3 i**	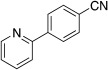	**4 i**	86
8	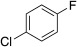	**3 j**	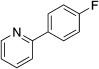	**4 j**	82
9^[c]^	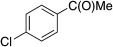	**3 a**	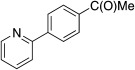	**4 a**	83
10	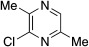	**3 k**	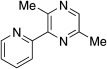	**4 k**	77
11	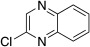	**3 l**	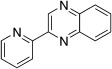	**4 l**	80
12	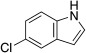	**3 m**	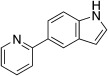	**4 m**	62
13	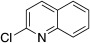	**3 n**	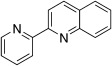	**4 n**	64
14	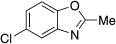	**3 o**	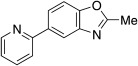	**4 o**	82

[a] General reaction conditions: 1.0 equiv aryl halide (1 mmol), 1.5 equiv MIDA boronate **1 a**, 5 mol % XphosPdcycle, 50 mol % Cu(OAc)_2_, 1.0 equiv DEA, 5.0 equiv K_3_PO_4_, 0.125 m DMF, 100 °C, 24 h. [b] Yield of isolated product. [c] 80 °C.

As shown in Table 3, these same reaction conditions were also successfully applied to very challenging couplings with a range of other 2-pyridyl MIDA boronate derivatives. Specifically, a series of both electron-rich (**1 b**–**e**) and electron-deficient (**1 f**–**h**) 2-pyridyl MIDA boronates, representing substructural motifs that appear in a wide variety of pharmaceuticals, materials, and ligands, were successfully coupled to a representative series of sterically and electronically deactivated aryl chlorides (entries 1–7).

**Table 3:** General cross-coupling of air-stable 2-pyridyl MIDA boronate derivatives 1 with deactivated aryl chlorides 3
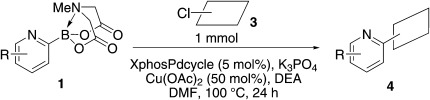
^[a]^

Entry	1		3		4		Yield [%]^[b]^
1	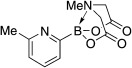	**1 b**	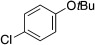	**3 b**	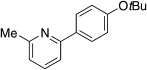	**4 p**	72
2	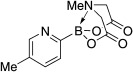	**1 c**	**3 b**		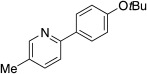	**4 q**	81
3	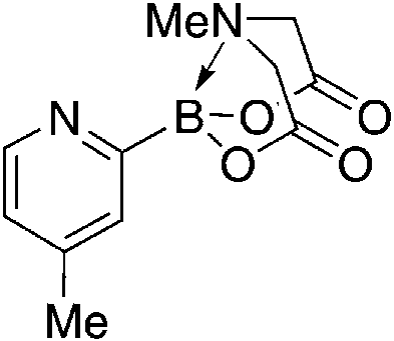	**1 d**	**3 b**		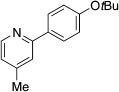	**4 r**	81
4	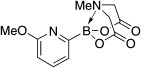	**1 e**	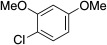	**3 h**	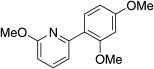	**4 s**	77
5	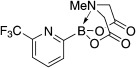	**1 f**	**3 b**		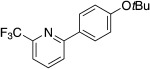	**4 t**	87
6	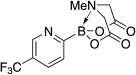	**1 g**	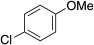	**3 c**	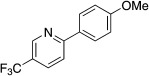	**4 u**	78
7	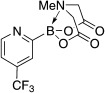	**1 h**	**3 b**		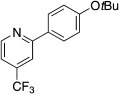	**4 v**	85

[a] General reaction conditions: 1 equiv aryl halide (1 mmol), 1.5 equiv MIDA boronate, 5 mol % XphosPdcycle, 50 mol % Cu(OAc)_2_, 5 equiv K_3_PO_4_, 0.125 m DMF, 100 °C, 24 h. [b] Yield of isolated product.

Finally, it is often the case that reaction conditions optimized for one class of halides or pseudohalides are less effective with others. However, the exact same conditions also promote the efficient coupling of **1 a** with a diverse range of electrophilic coupling partners (Table 4), including bromides (**8 a**–**e**), iodides (**8 f**–**i**), and triflates (**8 j**–**n**), with all three classes of electrophiles represented as deactivated, activated, and heteroaryl variants.

**Table 4:** General coupling of air-stable 2-pyridyl MIDA boronate 1 a with aryl and heteroaryl bromides, iodides, and triflates 8
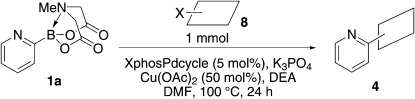
^[a]^

Entry	8		4		Yield [%]^[b]^
1	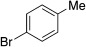	**8 a**	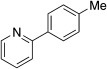	**4 w**	83
2	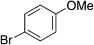	**8 b**	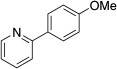	**4 c**	79
3	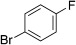	**8 c**	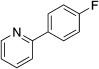	**4 j**	84
4	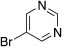	**8 d**	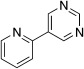	**4 x**	47
5	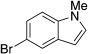	**8 e**	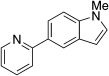	**4 y**	55
6	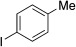	**8 f**	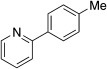	**4 w**	75
7	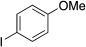	**8 g**	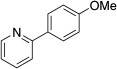	**4 c**	75
8	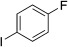	**8 h**	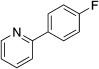	**4 j**	81
9^[c]^	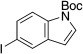	**8 i**	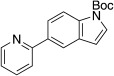	**4 z**	67
10	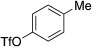	**8 j**	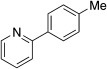	**4 w**	81
11	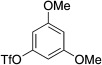	**8 k**	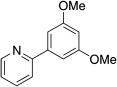	**4 aa**	87
12^[c]^	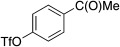	**8 l**	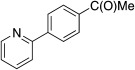	**4 a**	52
13	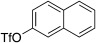	**8 m**	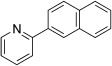	**4 bb**	90
14	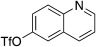	**8 n**	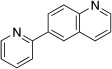	**4 cc**	80

[a] General reaction conditions: 1.0 equiv aryl halide (1 mmol), 1.5 equiv MIDA boronate **1 a**, 5 mol % XphosPdcycle, 50 mol % Cu(OAc)_2_, 1.0 equiv DEA, 5.0 equiv K_3_PO_4_, 0.125 m DMF, 100 °C, 24 h. [b] Yield of isolated product. [c] 80 °C. Boc=*tert*-butoxycarbonyl, Tf=trifluoromethanesulfonyl.

The ubiquity of the 2-pyridyl subunit in a wide range of important small molecules has for decades stimulated the search for an isolable 2-pyridyl borane that is both air-stable and a generally effective cross-coupling partner. The Cu(DEA)_2_/KOAc-promoted cross-coupling with the air-stable 2-pyridyl MIDA boronates described herein represents the first general solution to this problem. As the 2-pyridyl motif is in many ways the archetype for unstable boronic acids, this discovery has widespread implications for many other types of challenging cross-coupling processes. Moreover, this platform stands to immediately enable the more effective exploration of the functional potential of 2-pyridyl-containing small molecules for a wide range of important applications in science and medicine.

## Supplementary material

Detailed facts of importance to specialist readers are published as ”Supporting Information”. Such documents are peer-reviewed, but not copy-edited or typeset. They are made available as submitted by the authors.
