# Supplementary material for: A General Solution for the 2-Pyridyl Problem
Source: Angew Chem Int Ed Engl. 2012 Jan 27;51(11):2667–72. doi: 10.1002/anie.201108608 (PMC3433254; doi:10.1002/anie.201108608)

Angewandte Chemie
Eine Zeitschrift der Gesellschaft Deutscher Chemiker

Supporting Information

© Wiley-VCH 2012

69451 Weinheim, Germany

A General Solution for the 2-Pyridyl Problem**

*Graham R. Dick, Eric M. Woerly, and Martin D. Burke**

anie_201108608_sm_miscellaneous_information.pdf

Supporting Information

Part A

I.	General methods	S1-S2
II.	Figure 2C	S2-S3
III.	Table 1	S3-S4
IV.	Scheme 1	S4-S6
V.	Scheme 2	S6-S7
VI.	Scheme 3	S7-S9
VII.	2-pyridyl MIDA boronate consumption studies	S9-S11
VIII.	Scheme 4	S11-S12
IX.	Table 2	S12-S19
X.	Table 3	S19-S23
XI.	Table 4	S23-S29
XII.	Cross-coupling of 2-pyridyl MIDA boronate without a glovebox	S29

Part B

XIII.	NMR Spectra	S30-S79

I. General methods

Materials. Commercial reagents were obtained from Sigma-Aldrich, Fisher Scientific, TCI America, Frontier Scientific, Matrix Scientific, Combi-Blocks, Cambridge Isotopes Laboratories, Acros Organics, or Alfa Aesar and were used without further purification. Solvents were purified via passage through packed columns as described by Pangborn and coworkers[1] (THF, Et_2O, CH_3CN, CH_2Cl_2: dry neutral alumina; hexane, benzene, and toluene: dry neutral alumina and Q-5 reactant (copper(II) oxide on alumina); DMSO, DMF: activated molecular sieves). Water was deionized prior to use. MIDA boronates were prepared according to literature procedures.[2] The following MIDA boronates are now commercially available from Sigma-Aldrich http://sigma-aldrich.com/mida: **1a** 719390, **1b** 723959, **1e** 723053.

General experimental procedures. Unless otherwise noted, all reactions were performed in flame-dried glassware under argon. Organic solutions were concentrated via rotary evaporation under reduced pressure with a bath temperature of 20-60 ºC. Reactions were monitored by analytical thin layer chromatography (TLC) performed using the indicated solvent on E. Merck silica gel 60 F254 plates (0.25mm). Compounds were visualized by exposure to a UV lamp (λ = 254 or 366 nm) and treatment with a solution of $KMnO_4$ followed by brief heating with a Varitemp heat gun. MIDA boronates are compatible with standard silica gel chromatography, including standard loading techniques. Column chromatography was performed using standard methods[3] or with a Teledyne-Isco CombiFlash Rf purification system. Both methods were performed using Merck silica gel grade 9385 60 Å (230-400 mesh) or Fischer Florisil Absorbent (100-200 mesh). For loading, compounds were adsorbed onto non acid-washed Celite 545 (app.

[1] Pangborn, A. B.; Giardello, M. A; Grubbs, R. H.; Rosen, R. K.; Timmers, F.J. *Organometallics* **1996**, *15*, 1518-1520.
[2] Dick, G. R.; Knapp, D. M.; Gillis, E. P.; Burke, M. D. *Org. Lett.* **2010**, *12*, 2314-2317.
[3] Still, W.C.; Kahn, M.; Mitra, A. *J Org. Chem.* **1978**, *43*, 2923-2925.

4g/mmol crude product) i*n vacuo* from a diethyl ether solution. Specifically, in each case the crude residue was dissolved/suspended in diethyl ether and to the mixture was added Celite. The mixture was concentrated *in vacuo* to afford a free flowing powder which was then loaded on top of a silica gel column. To ensure quantitative transfer, this procedure was repeated with a small amount of diethyl ether and Celite to transfer any remaining residue.

Structural analysis. ^{1}H-NMR spectra were recorded at 23 °C on a Varian Unity 500 MHz spectrometer, Varian VXR-500 MHz spectrometer, Varian Unity Inova 400 MHz spectrometer, or Varian Unity 400 MHz spectrometer. Chemical shifts (δ) are reported in parts per million (ppm) downfield from tetramethylsilane and referenced to residual protium in the NMR solvent (CD_2HCN, δ = 1.93, center line, CD_2HCOCD_3, δ = 2.04, center line). Alternatively, NMR-solvents designated as "w/ TMS" were referenced to tetramethylsilane (δ = 0.00 ppm) added as an internal standard. Data are reported as follows: chemical shift, multiplicity (s = singlet, d = doublet, t = triplet, q = quartet, quint = quintet, sept = septet, m = multiplet, br = broad, app = apparent), coupling constant (*J*) in Hertz (Hz), and integration. ^{13}C NMR spectra were recorded at 23 °C on a Varian VXR-500 MHz spectrometer. Chemical shifts (δ) are reported in ppm downfield from tetramethylsilane and referenced to carbon resonances in the NMR solvent (Acetone-d_6, δ = 29.8, center line, DMSO-d_6 δ = 39.5, center line) or to added tetramethylsilane (δ = 0.00). High resolution mass spectra (HRMS) were performed by Furong Sun, Haijun Yao, and Elizabeth Eves at the University of Illinois, School of Chemical Sciences Mass Spectrometry Laboratory. Gas chromatography analysis was conducted on an Agilent Technologies 7890A instrument. GC yields are based on a biphenyl internal standard using an Agilent Technologies HP-5 column (part number 19091J-413). A standard GC method was used for all analyses: the oven was held at 75 ˚C for 0.5 min then heated to 100 ˚C over 3 min followed by heating to 230 ˚C over 9.5 min and held at 230 ˚C for 1 min. H_2 flow was 30 mL/min; Air flow was 400 mL/min; and N_2 flow was 25 mL/min. The t_r of the biphenyl internal standard 4.96 min; the t_r of 2-(4-tertbutoxyphenyl)-pyridine was 8.01 min; and the t_r of 2-(4-acetophenone)-pyridine was 8.03 min. X-ray crystallographic analysis of **5** was carried out by Dr. Danielle Gray, Dr. Yi Gui Gao, and Dr. Amy Fuller at the University of Illinois George L. Clark X-Ray facility. CHN analysis was performed by Marie Keel and ICP-MS analysis was performed by Dr. Rudiger Laufhutte at the University of Illinois Microanalysis Lab.

II. Figure 2C[4]

Under air to a 40 mL I-CHEM vial was added K_2CO_3 (691 mg, 5.0 mmol) and 2-pyridyl MIDA boronate **1a** (351 mg, 1.5 mmol). The vial was sealed with a septum cap and backfilled under argon. To the vial was then added the aryl chloride (1.0 mmol) via syringe. Into a separate 20 mL I-CHEM vial with septum cap and PTFE coated stirbar was added DMF (8 mL). Both vials were then brought into a glovebox. To the vial containing the chloride, K_2CO_3, and 2-pyridyl MIDA boronate was added copper (II) acetate (91 mg, 0.5 mmol) and biphenyl (154 mg, 1.0 mmol). To the vial containing DMF was added $Pd_2(dba)_3$ (14 mg, 0.015 mmol) and XPhos (29 mg, 0.06 mmol). The vial containing DMF, catalyst, and ligand was removed from the glovebox and incubated at 100 ˚C for 5 minutes with stirring and returned to the glovebox. This solution and the stirbar were transferred at ~40 ˚C to the 40 mL I-CHEM vial, and the vial was sealed. The

[4] Knapp, D. M.; Gillis, E. P.; Burke, M. D. *J. Am. Chem. Soc.* **2009**, *131*, 6961-6963.

vial was removed from the glovebox and a needle with a positive pressure of argon was inserted into the septum. To the vial by syringe was added isopropanol (2 mL, sparged with argon for 20 min and dried with 5 Å molecular sieves). The argon needle was removed from the vial and the reaction was heated to 100 ˚C with stirring for 4 h. The reaction was then allowed to cool to room temperature over 0.5 h. An aliquot was removed and filtered through a pad of silica gel washing with acetone into a GC vial and analyzed by gas chromatography using the biphenyl as an internal standard. GC yields were determined by the average of three injections. Each reaction was run in duplicate and the yields were averaged.

GC Yield for the coupling of 2-pyridyl MIDA boronate (**1a**) to 4-chloroacetophenone (**4a**): 72%

GC Yield for the coupling of 2-pyridyl MIDA boronate (**1a**) to 1-(tert-butoxy)-4-chlorobenzene (**4b**): 7%

III. Table 1

Cross-coupling of 2-pyridyl MIDA boronate to 1-(*tert*-butoxy)-4-chlorobenzene utilizing various alcohols and diols

Under air to a 40 mL I-CHEM vial equipped with PTFE coated stir bar was added XPhos Palladacycle, chloro(2-dicyclohexylphosphino-2',4',6'-tri-i-propyl-1,1'-biphenyl)[2-(2-aminoethyl)phenyl] palladium(II) methyl-t-butylether adduct, (37 mg, 0.05 mmol) and 2-pyridyl MIDA boronate **1a** (351 mg, 1.5 mmol). The vial was sealed with a septum cap and back-filled with argon. To the vial was added DMF (8 mL) and 1-(tert-butoxy)-4-chlorobenzene **3b** (174 µL, 1.00 mmol) by syringe. The vial was brought into a glove box where K_3PO_4 (1.061 g, 5.0 mmol), biphenyl (154 mg, 1.00 mmol), and $Cu(OAc)_2$ (91 mg, 0.5 mmol) were added. At this point, solid diols with high melting points were added. The vial was sealed with a septum cap and removed from the glove box. A needle under positive pressure of argon was inserted into the septum and using a syringe, the appropriate liquid or low melting point alcohol was added. The syringe was removed and the vial was heated to 100 ˚C with stirring for 24 h. The reaction was then allowed to cool to room temperature over 0.5 h. An aliquot was removed and filtered through a pad of silica gel washing with acetone into a GC vial and analyzed by gas chromatography using the biphenyl as the internal standard. GC yields were determined by the average of three injections. Each reaction was run in duplicate and the yields were averaged.

Entry	ROH	Quantity	Equivalents	% GC Yield
1	Isopropanol	229 µL	3	49
2	Methanol	121 µL	3	36
3	Ethanol	175 µL	3	39
4	*Tert*-butanol	285 µL	3	43
5	Ethylene Glycol	84 µL	1.5	51
6	Pinacol	177 mg	1.5	35
7	Neopentyl Glycol	156 mg	1.5	31
8	Diethanolamine (DEA)	144 µL	1.5	70

IV. Scheme 1

Kinetics of the transligation of 2-pyridyl MIDA boronate with diethanolamine (DEA)

Under air to a 20 mL I-CHEM vial was added 2-pyridyl MIDA boronate **1a** (394 mg, 1.69 mmol). The vial was sealed with a septum cap and back filled under argon. To the vial was added by syringe diethanolamine (170 µL, 1.69 mmol), *tert*-butylbenzene (260 µL, 1.69 mmol), and DMF-d_7 (9 mL). This vial was brought into a glovebox along with sixteen 2 mL vials with PTFE coated stirbars. Into each 2 mL vial was massed K_3PO_4 (66 mg, 0.31 mmol) and using a pipetman, 500 µL of the MIDA boronate containing stock solution was added to each vial. Each vial was then sealed with a PTFE lined cap and the vials were removed from the glovebox. The vials were heated to 100 ˚C with stirring. The remaining stock solution was transferred to a NMR tube and a ^{1}H-NMR was taken (nt = 2, d1 = 30) this represents t = 0. At regular intervals, two vials were removed from heating and allowed to cool to 23 ˚C after which the solution was transferred under air into a ^{1}H-NMR tube. A ^{1}H-NMR was then taken of these duplicate samples. The rate of transligation and decomposition of the MIDA boronate were determined by integration of the diastereotopic methylene protons of the MIDA boronate (δ 4.55 (d, J = 17 Hz, 2H)) and of the DEA boronate (δ 3.97 (m, 2H)) and comparing them to the integration of methyl protons of the *tert*-butyl benzene (δ 1.29 (s, 9H)). The major decomposition product was determined to be pyridine.

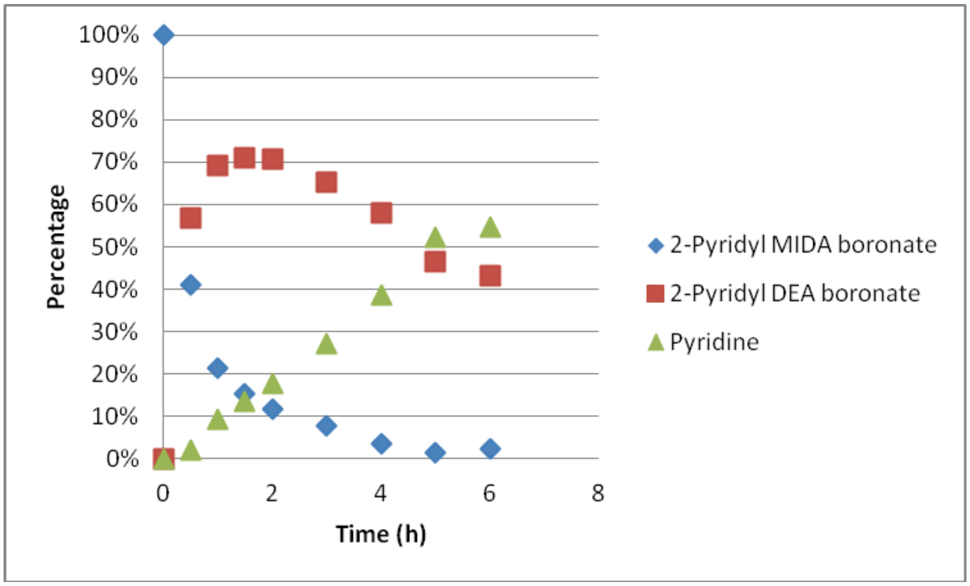

Synthesis of 2-pyridyl DEA boronate

2-pyridyl DEA boronate (5). To a 100 mL Schlenk flask with PTFE coated stirbar was added 2-pyridyl MIDA boronate **1a** (1.87 g, 8.00 mmol) and finely ground K_3PO_4 (8.78 g, 41.4 mmol) under positive argon pressure. The Schlenk flask was sealed with a septum and to the flask was then added acetonitrile (40 mL) and diethanolamine (1.6 mL, 17 mmol). The Schlenk flask was then heated to 80 ˚C with stirring for 7 h. After 7 h while still stirring at 80 ˚C, the acetonitrile

solution was cannulated into a tared 250 mL round bottom. The acetonitrile was then concentrated *in vacuo* to approximately 20 mL of solution and then allowed to cool to room temperature. A stirbar was added to the round bottom and with stirring, 100 mL of Et_2O was added to the flask over 0.5 h. After the addition was complete, the solution was allowed to stir for another 10 min before the stir bar was removed and the solution was decanted. The resulting solid was then placed under high vacuum for 10 min. To the round bottom was then added 20 mL of acetonitrile. The round bottom was sealed with a glass stopper and PTFE tape and heated to 80 ˚C for 15-20 min or until the solid completely dissolved. The stirbar was then removed from the round bottom and the reaction was allowed to cool to room temperature and sit overnight. The acetonitrile solution was decanted and the resulting solid was washed with small amounts of diethyl ether. The round bottom was then placed under high vacuum to remove residual acetonitrile and diethyl ether affording **5** as white spindly crystals (1.00 g, 65%).

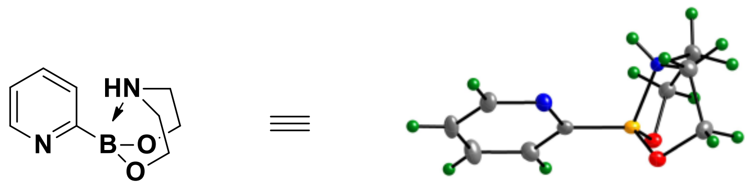

5

^{1}H-NMR (400 MHz, DMSO-d$_6$)

δ 8.52 (d, J = 4.0 Hz, 1H), 7.50 (t, J = 6.0 Hz, 1H), 7.40 (d, J = 6.0 Hz, 1H), 7.09 (t, J = 4.8 Hz, 1H), 7.02 (s, br, 1H), 3.84 (m, 2H), 3.72 (m, 2H), 3.15 (m, 2H), 2.82 (m, 2H).

^{13}C-NMR (125 MHz, DMSO-d$_6$)

δ 148.4, 133.4, 125.8, 120.9, 62.6, 50.8

^{11}B-NMR (128 MHz, DMSO-d$_6$)

δ 9.8

HRMS (ESI+)

Calculated for $C_9H_{14}BN_2O_2$ $(M+H)^+$:	193.1148
Found:	193.1147

Cross-coupling of 2-pyridyl DEA boronate to 1-(*tert*-butoxy)-4-chlorobenzene

Under air to a 40 mL I-CHEM vial equipped with a PTFE coated stir bar was added XPhos Palladacycle, chloro(2-dicyclohexylphosphino-2',4',6'-tri-i-propyl-1,1'-biphenyl)[2-(2-aminoethyl)phenyl] palladium(II) methyl-t-butylether adduct, (37 mg, 0.05 mmol). The vial was sealed with a septum cap and back-filled with argon. To the vial was added DMF (8 mL) and 1-(tert-butoxy)-4-chlorobenzene **3b** (174 µL, 1.0 mmol) by syringe. The vial was brought into a glove box where K_3PO_4 (1.061 g, 5.0 mmol), biphenyl (154 mg, 1.0 mmol), $Cu(OAc)_2$ (91 mg, 0.5 mmol), and 2-pyridyl DEA boronate **5** (288 mg, 1.5 mmol) were added. The vial was re-sealed with the septum cap and removed from the glove box and heated to 100 ˚C with stirring for 24 h. The reaction was then allowed to cool to room temperature over 0.5 h. An aliquot was removed and filtered through a pad of silica gel washing with acetone into a GC vial and

analyzed by gas chromatography using the biphenyl as the internal standard. GC yields were determined by the average of three injections. Each reaction was run in duplicate and the yields were averaged. Based on the biphenyl internal standard, the yield was 9%.

Slow addition cross-coupling of 2-pyridyl DEA boronate to 1-(*tert*-butoxy)-4-chlorobenzene

Under air to a 20 mL I-CHEM vial equipped with PTFE coated stir bar was added XPhos Palladacycle, chloro(2-dicyclohexylphosphino-2',4',6'-tri-i-propyl-1,1'-biphenyl)[2-(2-aminoethyl)phenyl] palladium(II) methyl-t-butylether adduct, (20 mg, 0.027 mmol). The vial was sealed with a septum cap and back-filled with argon. To the vial was added DMF (2 mL) and 1-(tert-butoxy)-4-chlorobenzene **3b** (87 µL, 0.5 mmol) by syringe. Into a second 20 mL I-CHEM vial with septum cap was added DMF (10 mL). The vials were brought into a glove box where K_3PO_4 (555 mg, 2.61 mmol), and $Cu(OAc)_2$ (46 mg, 0.25 mmol) were added to the vial containing the chloride. 2-pyridyl DEA boronate **5** (178 mg, 0.93 mmol) was added to the vial containing just 10 mL DMF. Both vials were re-sealed with septum caps and removed from the glove box. A needle under positive pressure of argon was inserted into both vials and the vial containing the catalyst and chloride was heated to 100 ˚C with stirring. A 10 mL syringe with a 20 gauge steel needle was used to draw up 8 mL of the 2-pyridyl DEA boronate **5** solution. This syringe was fitted into a syringe pump and the needle was inserted into the vial containing the catalyst and chloride. The 2-pyridyl DEA boronate **5** solution was added slowly over 4 h 15 min. The needles were then removed and the reaction was stirred for a further 20 h at 100 ˚C. The reaction was then allowed to cool to room temperature over 0.5 h. The reaction was transferred to a 60 mL separatory funnel, diluted with 10 mL of 2N HCl and shaken. The reaction was then diluted with 10 mL 2N NaOH and shaken. The resulting aqueous solution was extracted twice with 10 mL of diethyl ether. The organic layers were combined and washed with 5 mL of brine. The organic layers were dried using Na_2SO_4, filtered, and concentrated *in vacuo*. The resulting residue was adsorbed onto Celite and subjected to Florisil chromatography (EtOAc:Hexanes 5:95 → 20:80) to afford **4b** as a pale yellow solid (15 mg, 13%).

V. Scheme 2

Preparation of $Cu(DEA)_2$

To a 100 mL Schlenk flask in the glovebox was added $Cu(OAc)_2$ (229 mg, 1.26 mmol), K_3PO_4 (2.49 g, 11.7 mmol) and a PTFE coated stirbar. The Schlenk flask was sealed with a septum and removed from the glovebox. The Schlenk flask was attached to a vacuum manifold and placed under argon maintenance. To the flask was added diethanolamine (360 µL, 3.75 mmol) and DMF (20 mL). The Schlenk flask was then heated at 100 ˚C with stirring for 15 min. An aliquot was removed from the crude reaction mixture and was found to contain $Cu(DEA)_2$ by ESI HRMS.

The reaction was then allowed to cool to room temperature and the solids were allowed to settle. The DMF solution was decanted and isopropanol (20 mL) was introduced, dissolving the product. The reaction was then air-free filtered into a tared 200 mL Schlenk flask. The filtrate was concentrated using the vacuum manifold to ¼ of its original volume. With stirring, 20 mL of dry, degassed acetone was slowly added precipitating a reddish purple solid. The solution was

decanted and the solid was washed twice with 5 mL of acetone. The solid was dried on the vacuum line for 0.5 h to afford the product as a reddish purple powder with white specks (260 mg, 76%).

A NMR (in CD_3OD) was taken of the product which indicated that potassium acetate was contaminating the product.

HRMS (ESI+)

Calculated for $C_8H_{21}CuN_2O_4$ $(M+H)^+$: 272.0797

Found: 272.0793

VI. Scheme 3

Synthesis and purification of $Cu(DEA)_2$

To a 100 mL Schlenk flask in the glovebox was added $CuCl_2$ (840 mg, 6.3 mmol), K_3PO_4 (6.87g, 32.4 mmol) and a PTFE coated stirbar. The Schlenk flask was sealed with a septum and removed from the glovebox. The Schlenk flask was attached to a vacuum manifold and placed under argon maintenance. To the flask was added diethanolamine (3.0 mL, 31.0 mmol) and isopropanol (50 mL). The Schlenk flask was then heated at 60 ˚C with stirring for 15 h. The reaction was then air-free filtered into a tared 200 mL Schlenk flask. The filtrate was concentrated using the vacuum manifold to ½ of its original volume. With stirring, 100 mL of dry, degassed acetone was slowly added, precipitating a reddish purple solid. The solution was decanted and the solid was washed twice with 20 mL of acetone. The solid was dried on the vacuum line for 0.5 h to afford the product as a reddish purple powder (1.38 g, 81%).

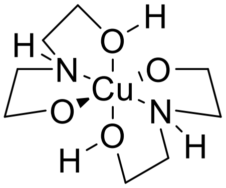

IR (nujol, cm^{-1})

ν 3067, 1181, 1115, 1093, 1067, 1043, 1023

CHN/ICP-MS

Calculated: C: 35.35%, H: 7.42%, N: 10.31%, Cu: 23.38%

Found: C: 35.45%, H: 7.69%, N: 9.96%, Cu: 22.60%

HRMS (ESI+)

Calculated for $C_8H_{21}CuN_2O_4$ $(M+H)^+$: 272.0797

Found: 272.0804

Cross-coupling using $Cu(DEA)_2$ and KOAc.

Under air to a 40 mL I-CHEM vial equipped with PTFE coated stir bar was added 2-pyridyl MIDA boronate **1a** (351 mg, 1.5 mmol), XPhos Palladacycle, chloro(2-dicyclohexylphosphino-2',4',6'-tri-i-propyl-1,1'-biphenyl)[2-(2-aminoethyl)phenyl] palladium(II) methyl-t-butylether adduct, (37 mg, 0.05 mmol), and KOAc (98 mg, 1.0 mmol). The vial was sealed with a septum cap and back-filled with argon. To the vial was added DMF (8 mL) and 1-(tert-butoxy)-4-chlorobenzene **3b** (174 µL, 1.0 mmol) by syringe. The vial was brought into a glove box where K_3PO_4 (1.061 g, 5.0 mmol), biphenyl (154 mg, 1.0 mmol), and $Cu(DEA)_2$ (136 mg, 0.5 mmol) were added. The vial was re-sealed with the septum cap and removed from the glove box and heated to 100 ˚C with stirring for 24 h. The reaction was then allowed to cool to room temperature over 0.5 h. An aliquot was removed and filtered through a pad of silica gel washing with acetone into a GC vial and analyzed by gas chromatography using the biphenyl as the internal standard. GC yields were determined by the average of three injections. Each reaction was run in duplicate and the yields were averaged. Based on the biphenyl internal standard, the yield was 84%.

Cross-coupling using no copper.

Under air to a 40 mL I-CHEM vial equipped with PTFE stir bar was added 2-pyridyl MIDA boronate **1a** (351 mg, 1.5 mmol) and XPhos Palladacycle, chloro(2-dicyclohexylphosphino-2',4',6'-tri-i-propyl-1,1'-biphenyl)[2-(2-aminoethyl)phenyl] palladium(II) methyl-t-butylether adduct, (37 mg, 0.05 mmol). The vial was sealed with a septum cap and back-filled with argon. To the vial was added DMF (8 mL) and 1-(tert-butoxy)-4-chlorobenzene **3b** (174 µL, 1.0 mmol) by syringe. The vial was brought into a glove box where K_3PO_4 (1.061 g, 5.0 mmol) and biphenyl (154 mg, 1.0 mmol) were added. The vial was re-sealed with the septum cap and removed from the glove box and heated to 100 ˚C with stirring for 24 h. The reaction was then allowed to cool to room temperature over 0.5 h. An aliquot was removed and filtered through a pad of silica gel washing with acetone into a GC vial and analyzed by gas chromatography using the biphenyl as the internal standard. GC yields were determined by the average of three injections. Each reaction was run in duplicate and the yields were averaged. Based on the biphenyl internal standard, the yield was <5%.

Cross-coupling using KOAc and no copper.

Under air to a 40 mL I-CHEM vial equipped with PTFE coated stir bar was added 2-pyridyl MIDA boronate **1a** (351 mg, 1.5 mmol), XPhos Palladacycle, chloro(2-dicyclohexylphosphino-2',4',6'-tri-i-propyl-1,1'-biphenyl)[2-(2-aminoethyl)phenyl] palladium(II) methyl-t-butylether adduct, (37 mg, 0.05 mmol), and KOAc (98 mg, 1.0 mmol). The vial was sealed with a septum cap and back-filled with argon. To the vial was added DMF (8 mL) and 1-(tert-butoxy)-4-chlorobenzene **3b** (174 µL, 1.0 mmol) by syringe. The vial was brought into a glove box where K_3PO_4 (1.061 g, 5.0 mmol) and biphenyl (154 mg, 1.0 mmol) were added. The vial was re-sealed with the septum cap and removed from the glove box and heated to 100 ˚C with stirring for 24 h. The reaction was then allowed to cool to room temperature over 0.5 h. An aliquot was removed and filtered through a pad of silica gel washing with acetone into a GC vial and analyzed by gas chromatography using the biphenyl as the internal standard. GC yields were determined by the average of three injections. Each reaction was run in duplicate and the yields were averaged. Based on the biphenyl internal standard, the yield was <5%.

Cross-coupling using $Cu(OAc)_2$ and KOAc.

Under air to a 40 mL I-CHEM vial equipped with PTFE coated stir bar was added 2-pyridyl MIDA boronate **1a** (351 mg, 1.5 mmol), XPhos Palladacycle, chloro(2-dicyclohexylphosphino-2',4',6'-tri-i-propyl-1,1'-biphenyl)[2-(2-aminoethyl)phenyl] palladium(II) methyl-t-butylether adduct, (37 mg, 0.05 mmol), and KOAc (98 mg, 1.0 mmol). The vial was sealed with a septum cap and back-filled with argon. To the vial was added DMF (8 mL) and 1-(tert-butoxy)-4-chlorobenzene **3b** (174 µL, 1.0 mmol) by syringe. The vial was brought into a glove box where K_3PO_4 (1.061 g, 5.0 mmol), biphenyl (154 mg, 1.0 mmol), and $Cu(OAc)_2$ (91 mg, 0.5 mmol) were added. The vial was re-sealed with the septum cap and removed from the glove box and heated to 100 ˚C with stirring for 24 h. The reaction was then allowed to cool to room temperature over 0.5 h. An aliquot was removed and filtered through a pad of silica gel washing with acetone into a GC vial and analyzed by gas chromatography using the biphenyl as the internal standard. GC yields were determined by the average of three injections. Each reaction was run in duplicate and the yields were averaged. Based on the biphenyl internal standard, the yield was 38%.

Cross-coupling using $Cu(DEA)_2$.

Under air to a 40 mL I-CHEM vial equipped with PTFE coated stir bar was added 2-pyridyl MIDA boronate **1a** (351 mg, 1.5 mmol) and XPhos Palladacycle, chloro(2-dicyclohexylphosphino-2',4',6'-tri-i-propyl-1,1'-biphenyl)[2-(2-aminoethyl)phenyl] palladium(II) methyl-t-butylether adduct, (37 mg, 0.05 mmol). The vial was sealed with a septum cap and back-filled with argon. To the vial was added DMF (8 mL) and 1-(tert-butoxy)-4-chlorobenzene **3b** (174 µL, 1.0 mmol) by syringe. The vial was brought into a glove box where K_3PO_4 (1.061 g, 5.0 mmol), biphenyl (154 mg, 1.0 mmol), and $Cu(DEA)_2$ (136 mg, 0.5 mmol) were added. The vial was re-sealed with the septum cap and removed from the glove box and heated to 100 ˚C with stirring for 24 h. The reaction was then allowed to cool to room temperature over 0.5 h. An aliquot was removed and filtered through a pad of silica gel washing with acetone into a GC vial and analyzed by gas chromatography using the biphenyl as the internal standard. GC yields were determined by the average of three injections. Each reaction was run in duplicate and the yields were averaged. Based on the biphenyl internal standard, the yield was 50%.

VII. 2-pyridyl MIDA boronate consumption studies

Under air, to a 20 mL I-CHEM vial was added 2-pyridyl MIDA boronate **1a** (623 mg, 2.7 mmol), *tert*-butyl benzene (410 µL, 2.7 mmol), and DMF-d_7 (14.0 mL). The vial was sealed with a septum cap and backfilled under argon. This solution was taken into a glovebox. To six 7 mL vials containing stir bars was added K_3PO_4 (80.6 mg each, 0.38 mmol). To six 7 mL vials containing stir bars was added K_3PO_4 (80.6 mg each, 0.38 mmol) and KOAc (7.5 mg each, 0.076 mmol). To five 7 mL vials containing stir bars was added K_3PO_4 (80.6 mg each, 0.38 mmol) and $Cu(DEA)_2$ (10.2 mg each, 0.038 mmol). To five 7 mL vials containing stir bars was added K_3PO_4 (80.6 mg each, 0.38 mmol), KOAc (7.5 mg each, 0.076 mmol), and $Cu(DEA)_2$ (10.2 mg each, 0.038 mmol). A portion (0.6 mL) of the MIDA boronate containing stock solution was added to each of these vials. The vials were capped, removed from the glovebox, and heated to

100 °C with stirring. At intervals, a vial from each set was removed and cooled to room temperature. A ^{1}H-NMR was taken of the solutions taking two transients with d1=30. A ^{1}H-NMR was taken of the initial stock solution. The quantity of 2-pyridyl MIDA boronate remaining was determined by integrating the diastereotopic methylene protons of the MIDA boronate (δ 4.55 (d, *J* = 17 Hz, 2H)) and comparing to the integration of the methyl protons of the *tert*-butyl benzene internal standard (δ 1.28 (s, 9H)).

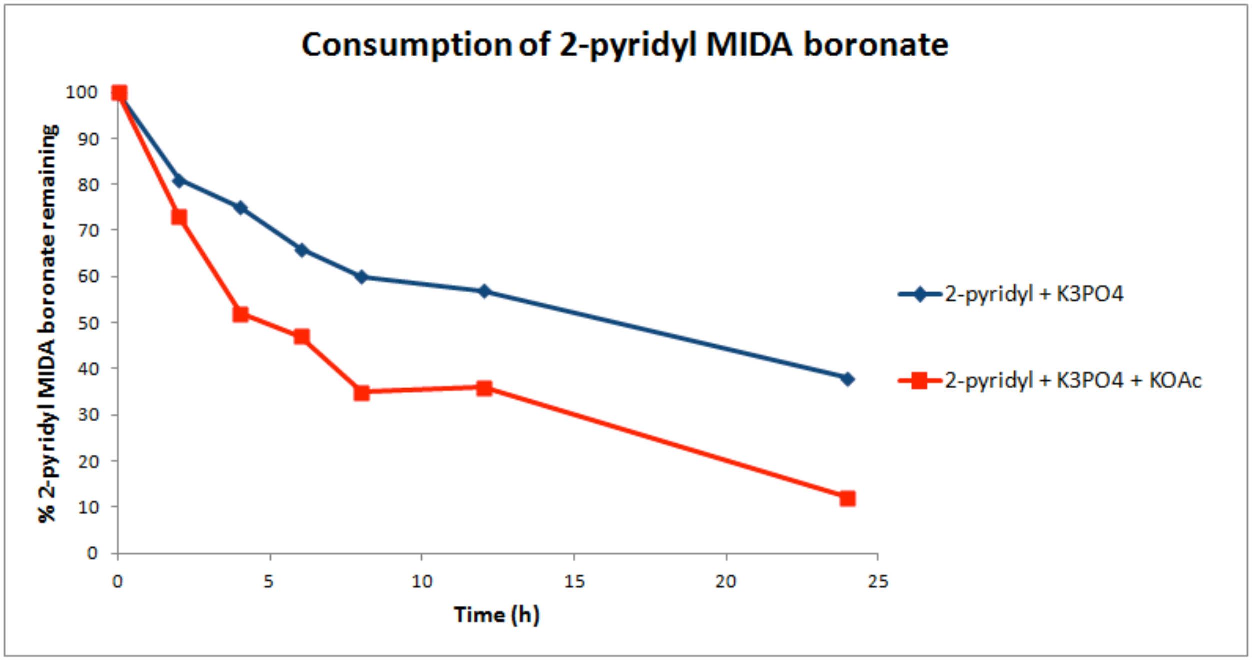

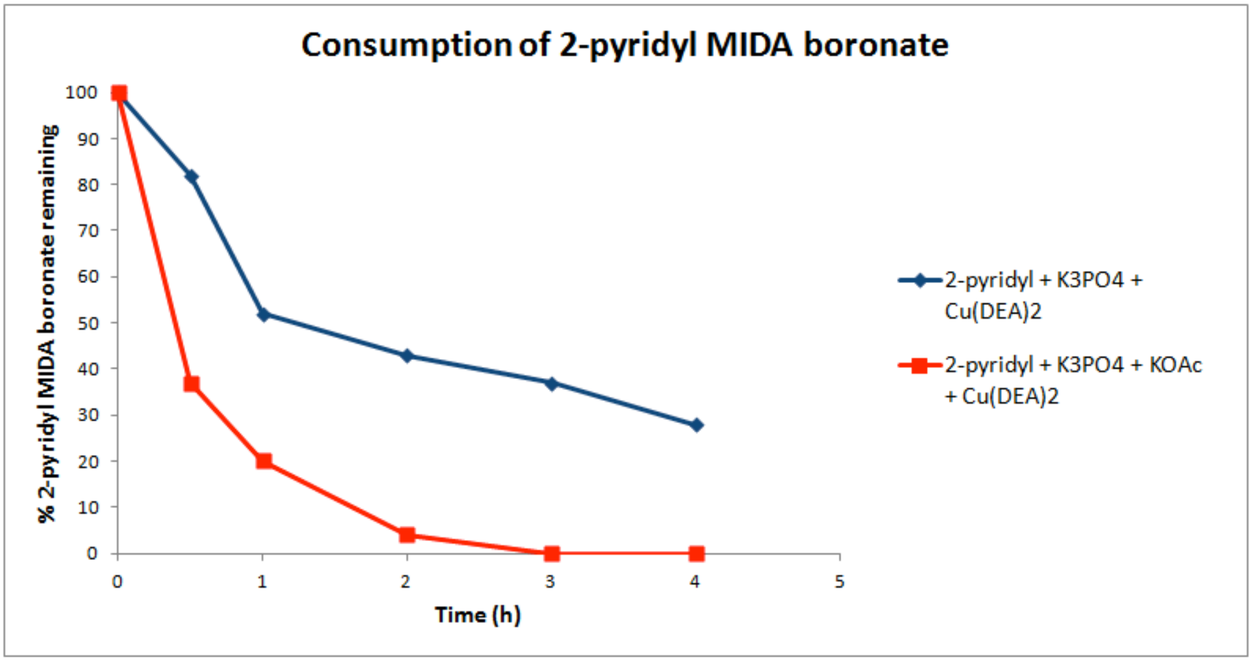

VIII. Scheme 4

Under air to a 40 mL I-CHEM vial equipped with PTFE coated stir bar was added XPhos Palladacycle, chloro(2-dicyclohexylphosphino-2',4',6'-tri-i-propyl-1,1'-biphenyl)[2-(2-aminoethyl)phenyl] palladium(II) methyl-t-butylether adduct, (37 mg, 0.05 mmol) and 2-pyridyl MIDA boronate **1a** (351 mg, 1.5 mmol). The vial was sealed with a septum cap and back-filled with argon. To the vial was added DMF (8 mL) and 1-(tert-butoxy)-4-chlorobenzene **3b** (174 µL, 1.00 mmol) by syringe. The vial was brought into a glove box where K_3PO_4 (1.061 g, 5.0 mmol), biphenyl (154 mg, 1.00 mmol), and $Cu(OAc)_2$ (91 mg, 0.5 mmol) were added. The vial was sealed with a septum cap and removed from the glove box. A needle under positive pressure of argon was inserted into the septum and using a syringe, the appropriate alcohol was added. The syringe was removed and the vial was heated to 100 ˚C with stirring for 24 h. The reaction was then allowed to cool to room temperature over 0.5 h. An aliquot was removed and filtered through a pad of silica gel washing with acetone into a GC vial and analyzed by gas chromatography using the biphenyl as the internal standard. GC yields were determined by the average of three injections. Each reaction was run in duplicate and the yields were averaged.

Entry	ROH	Quantity	Equivalents	% GC Yield
1	Diethanolamine (DEA)	144 µL	1.5	70
2	Diethanolamine (DEA)	96 µL	1	81
3	Diethanolamine (DEA)	2.4 mL	25	24
4	Diethanolamine (DEA)	48 µL	0.5	73

Cross-coupling on a 1 mmol scale.

Under air to a 40 mL I-CHEM vial equipped with PTFE coated stir bar was added XPhos Palladacycle, chloro(2-dicyclohexylphosphino-2',4',6'-tri-i-propyl-1,1'-biphenyl)[2-(2-aminoethyl)phenyl] palladium(II) methyl-t-butylether adduct, (42 mg, 0.057 mmol) and 2-pyridyl MIDA boronate **1a** (357 mg, 1.53 mmol). The vial was sealed with a septum cap and back-filled with argon. To the vial was added DMF (20 mL), 1-(tert-butoxy)-4-chlorobenzene **3b** (175 µL, 1.0 mmol), and diethanolamine (95 µL, 0.99 mmol) by syringe. The vial was brought into a glove box where K_3PO_4 (1.086 g, 5.1 mmol) and $Cu(OAc)_2$ (92 mg, 0.51 mmol) were added. The vial was sealed with a PTFE lined cap and removed from the glove box. The vial was heated to 100 ˚C with stirring for 24 h. The reaction was then allowed to cool to room temperature over 0.5 h. The reaction was transferred to a 60 mL separatory funnel, diluted with 10 mL of 2N HCl and shaken. The reaction was then diluted with 10 mL 2N NaOH and shaken. The resulting aqueous solution was extracted three times with 10 mL of diethyl ether. The organic layers were combined and washed with 10 mL of brine. The organic layers were dried using Na_2SO_4, filtered, and concentrated *in vacuo*. The resulting residue was adsorbed onto Celite and subjected to Florisil chromatography (EtOAc:Hexanes 5:95 → 20:80) to afford **4b** as a pale yellow solid (215 mg, 94%).

IX. Table 2

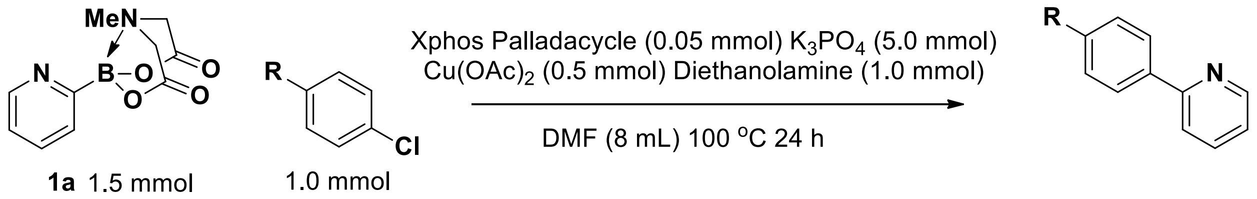

General procedure for the cross-coupling of 2-pyridyl MIDA boronate (Table 2, Table 3, and Table 4).

Under air to a flame-dried 40 mL I-CHEM vial equipped with PTFE coated stir bar was added halide (1.0 mmol), XPhos Palladacycle, chloro(2-dicyclohexylphosphino-2',4',6'-tri-i-propyl-1,1'-biphenyl)[2-(2-aminoethyl)phenyl] palladium(II) methyl-t-butylether adduct, (37 mg, 0.05 mmol), and 2-pyridyl MIDA boronate **1a** (351 mg, 1.5 mmol). The vial was sealed with a septum cap and back-filled with argon. To the vial was added DMF (8 mL) and diethanolamine (96 µL, 1.0 mmol) via syringe. The vial was brought into a glove box where K_3PO_4 (1.061 g, 5.0 mmol) and $Cu(OAc)_2$ (91 mg, 0.5 mmol) were added. The vial was sealed with a septum cap and removed from the glove box. The vial was heated to 100 ˚C with stirring for 24 h. The vial was then cooled to 23 ˚C over 0.5 h. To the vial was added 10 mL of 2N HCl and the resulting solution was shaken. To the vial was then added 10 mL of 2N NaOH and the resulting solution was shaken and transferred using ~20mL of Et_2O to a 100 mL separatory funnel. The mixture was shaken and the organic phase was separated. The aqueous phase was extracted twice with 10 mL of Et_2O. The organic fractions were combined, washed with 10 mL of brine, and dried with Na_2SO_4. The solution was then filtered and concentrated *in vacuo*. The resulting residue was adsorbed onto Celite and subjected to column chromatography on SiO_2 or Florisil to afford the purified product.

2-(4-(tert-butoxy)phenyl)pyridine (4b). The general procedure was followed using MIDA boronate **1a** (360 mg, 1.54 mmol) and chloride **3b** (175 µL, 1.00 mmol). The crude product was subjected to silica gel chromatography (EtOAc:Hexanes 5:95 → 20:80) to afford **4b** as a pale yellow solid (199 mg, 88%).

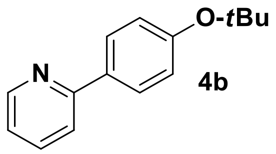

4b

TLC (EtOAc:Hexanes 20:80)

R_f = 0.4, visualized by UV (λ = 254 nm)

^{1}H-NMR (500 MHz, CD_3CN)

δ 8.61 (d, *J* = 5.0 Hz, 1H), 7.96 (d, *J* = 9.0 Hz, 2H), 7.79 (m, 2H), 7.24 (m, 1H), 7.08 (d, *J* = 8.5 Hz, 2H), 1.36 (s, 9H)

^{13}C-NMR (125 MHz, Acetone-d_6)

δ 157.6, 157.3, 150.3, 137.6, 134.9, 128.1, 124.5, 122.6, 120.3, 79.0, 29.1

HRMS (ESI+)
Calculated for $C_{15}H_{18}NO$ $(M+H)^+$: 228.1388
Found: 228.1388

2-(4-methoxyphenyl)pyridine (4c) [Table 2, entry 1]. The general procedure was followed using MIDA boronate **1a** (361 mg, 1.54 mmol) and chloride **3c** (122.5 µL, 1.00 mmol). The crude product was subjected to Florisil chromatography (EtOAc:Hexanes 5:95 → 20:80) to afford **4c** as an off-white solid (142 mg, 76%).

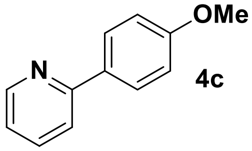

4c

TLC (EtOAc:Hexanes 20:80)
R_f = 0.29, visualized by UV (λ = 254 nm)

^{1}H-NMR (500 MHz, CD_3CN)
δ 8.59 (d, *J* = 4.5 Hz, 1H), 8.00 (d, *J* = 6.5 Hz, 2H), 7.77 (m, 2H), 7.22 (dt, *J* = 3.5, 5 Hz, 1H), 7.02 (d, *J* = 9.0 Hz, 2H), 3.38 (s, 3H).

^{13}C-NMR (125 MHz, Acetone-d_6)
δ 161.5, 157.3, 150.3, 137.5, 132.6, 128.7, 122.3, 120.0, 114.7, 55.6

HRMS (ESI+)
Calculated for $C_{12}H_{12}NO$ $(M+H)^+$: 186.0919
Found: 186.0914

2-(3-methoxyphenyl)pyridine (4d) [Table 2, entry 2]. The general procedure was followed using MIDA boronate **1a** (356 mg, 1.52 mmol) and chloride **3d** (125 µL, 1.02 mmol). The crude product was subjected to Florisil chromatography (EtOAc:Hexanes 5:95 → 20:80) to afford **4d** as a yellow oil (182 mg, 96%).

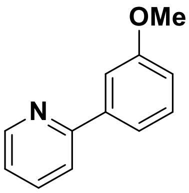

4d

TLC (EtOAc:Hexanes 20:80)
R_f = 0.3, visualized by UV (λ = 254 nm)

^{1}H-NMR (500 MHz, Acetone-d_6)
δ 8.65 (d, *J* = 5.5 Hz, 1H), 7.92 (dt, *J* = 1.0, 8.0 Hz, 1H), 7.84 (td, *J* = 2.0, 7.5 Hz, 1H), 7.71 (t, *J* = 2.0 Hz, 1H), 7.66 (dt, *J* = 1.5, 8.0 Hz, 1H), 7.38 (t, *J* = 8.0 Hz, 1H), 7.31 (ddd, *J* = 1.0, 5.0, 7.5 Hz, 1H), 6.99 (ddd, *J* = 0.5, 2.5, 7.5 Hz, 1H), 3.87 (s, 3H).

^{13}C-NMR (125 MHz, Acetone-d_6)
δ 161.0, 157.3, 150.3, 141.6, 137.6, 130.4, 123.2, 121.0, 119.7, 115.4, 112.8, 55.5

HRMS (ESI+)

Calculated for $C_{12}H_{12}NO$ $(M+H)^+$:	186.0919
Found:	186.0920

2-(2-methoxyphenyl)pyridine (4e) [Table 2, entry 3]. The general procedure was followed using MIDA boronate **1a** (365 mg, 1.56 mmol) and chloride **3e** (130 µL, 1.02 mmol). The crude product was subjected to Florisil chromatography (EtOAc:Hexanes 5:95 → 20:80) to afford **4e** as a yellow oil (142 mg, 75%).

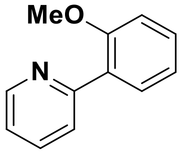

4e

TLC (EtOAc:Hexanes 20:80)
R_f = 0.29, visualized by UV (λ = 254 nm)

^{1}H-NMR (500 MHz, Acetone-d_6)
δ 8.64 (d, *J* = 5.0 Hz, 1H), 7.92 (dt, *J* = 1.0, 8.0 Hz, 1H), 7.85 (dd, *J* = 2.0, 8.0 Hz, 1H), 7.75 (td, *J* = 2.0, 8.0 Hz, 1H), 7.38 (td, *J* = 2.0, 8.0 Hz, 1H), 7.25 (ddd, *J* = 1.0, 6.0, 7.5 Hz, 1H), 7.12 (d, *J* = 8.0 Hz, 1H), 7.05 (td, *J* = 1.0, 8.0 Hz, 1H), 3.88 (s, 3H).

^{13}C-NMR (125 MHz, Acetone-d_6)
δ 158.1, 156.6, 150.1, 136.2, 131.9, 130.7, 129.7, 125.7, 122.4, 121.4, 112.4, 55.9

HRMS (ESI+)

Calculated for $C_{12}H_{12}NO$ $(M+H)^+$:	186.0919
Found:	186.0914

2-(*o*-tolyl)pyridine (4f) [Table 2, entry 4]. The general procedure was followed using MIDA boronate **1a** (358 mg, 1.53 mmol) and chloride **3f** (117.5 µL, 1.01 mmol). The crude product was subjected to Florisil chromatography (EtOAc:Hexanes 5:95 → 15:85) to afford **4f** as a yellow oil (134 mg, 79%).

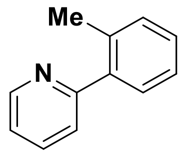

4f

TLC (EtOAc:Hexanes 17:83)
R_f = 0.35, visualized by UV (λ = 254 nm)

^{1}H-NMR (500 MHz, Acetone-d$_6$)

δ 8.65 (d, *J* = 5.0 Hz, 1H), 7.85 (td, *J* = 2.0, 8.0 Hz, 1H), 7.48 (dt, *J* = 1.0, 8.0 Hz, 1H), 7.39 (d, *J* = 2.0, 7.0 Hz, 1H), 7.29 (m, 4H), 2.35 (s, 3H).

^{13}C-NMR (125 MHz, Acetone-d$_6$)

δ 160.7, 149.8, 141.4, 137.0, 136.5, 131.4, 130.4, 128.8, 126.5, 124.7, 122.5, 20.6

HRMS (ESI+)

Calculated for $C_{12}H_{12}N$ $(M+H)^+$:	170.0970
Found:	170.0962

2-(2,5-dimethylphenyl)pyridine (4g) [Table 2, entry 5]. The general procedure was followed using MIDA boronate **1a** (350 mg, 1.50 mmol) and chloride **3g** (135 µL, 1.01 mmol). The crude product was subjected to Florisil chromatography (EtOAc:Hexanes 5:95 → 15:85) to afford **4g** as a yellow oil (134 mg, 72%).

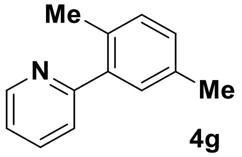

4g

TLC (EtOAc:Hexanes 17:83)

R_f = 0.32, visualized by UV (λ = 254 nm)

^{1}H-NMR (500 MHz, Acetone-d$_6$)

δ 8.64 (d, *J* = 5.0 Hz, 1H), 7.83 (td, *J* = 2.0, 8.0 Hz, 1H), 7.46 (dt, *J* = 1.0, 7.5 Hz, 1H), 7.30 (ddd, *J* = 1.0, 5.0, 7.5 Hz, 1H), 7.29 (s, 1H), 7.19 (d, *J* =20 Hz, 1H), 7.10 (dd, *J* = 1.0, 8.0 Hz, 1H), 2.32 (s, 3H), 2.30 (s, 3H).

^{13}C-NMR (125 MHz, Acetone-d$_6$)

δ 160.9, 149.8, 141.3, 136.9, 135.7, 133.4, 131.4, 131.1, 129.5, 124.7, 122.4, 20.9, 20.1

HRMS (ESI+)

Calculated for $C_{13}H_{14}N$ $(M+H)^+$:	184.1126
Found:	184.1120

2-(2,4-dimethoxyphenyl)pyridine (4h) [Table 2, entry 6]. The general procedure was followed using MIDA boronate **1a** (355 mg, 1.52 mmol), chloride **3h** (150 µL, 1.01 mmol), and running the reaction at 80 ˚C for 24 h. The crude product was subjected to Florisil chromatography (EtOAc:Hexanes 50:50) to afford **4h** as a pale yellow oil (107 mg, 49%).

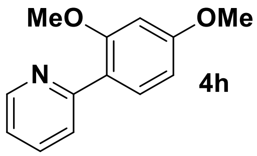

4h

TLC (EtOAc:Hexanes 50:50)
R_f = 0.45, visualized by UV (λ = 254 nm)

^{1}H-NMR (400 MHz, Acetone-d_6)
δ 8.59 (d, *J* = 4.8 Hz, 1H), 7.89 (t, *J* = 9.8 Hz, 2H), 7.71 (td, *J* = 2.0, 7.4 Hz, 1H), 7.18 (ddd, *J* = 1.4, 4.8, 7.4 Hz, 1H), 6.65 (m, 2H), 3.88 (s, 3H), 3.85 (s, 3H).

^{13}C-NMR (125 MHz, Acetone-d_6)
δ 162.4, 159.3, 156.4, 149.9, 136.2, 132.8, 125.2, 122.3, 121.8, 106.1, 99.3, 55.8, 55.6

HRMS (ESI+)

Calculated for $C_{13}H_{14}NO_2$ $(M+H)^+$:	216.1025
Found:	216.1022

4-(pyridin-2-yl)benzonitrile (4i) [Table 2, entry 7]. The general procedure was followed using MIDA boronate **1a** (351 mg, 1.50 mmol) and chloride **3i** (138 mg, 1.00 mmol). The crude product was subjected twice to silica gel chromatography (first Run, EtOAc:Hexanes 2.5:97.5 → 20:80; second Run, EtOAc:Hexanes 2.5:97.5 → 50:50) to afford **4i** as a white crystalline solid (159 mg, 86%). Characterization was consistent with literature.[4]

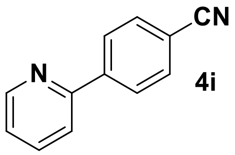

4i

2-(4-fluorophenyl)pyridine (4j) [Table 2, entry 8]. The general procedure was followed using MIDA boronate **1a** (361 mg, 1.54 mmol) and chloride **3j** (107.5 µL, 1.01 mmol). The crude product was subjected to silica gel chromatography (EtOAc:Hexanes 5:95 → 20:80) to afford **4j** as a pale yellow solid (143 mg, 82%).

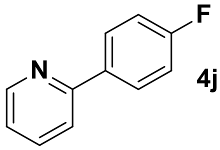

4j

TLC (EtOAc:Hexanes 20:80)
R_f = 0.42, visualized by UV (λ = 254 nm)

^{1}H-NMR (500 MHz, CD_3CN)
δ 8.63 (d, *J* = 4.5 Hz, 1H), 8.08 (dq, *J* = 5.5, 9.0 Hz, 2H), 7.81 (d, *J* = 3.5 Hz, 2H), 7.28 (q, *J* = 4.5, 8.5 Hz 1H), 7.22 (t, *J* = 9.0 Hz, 2H).

^{13}C-NMR (125 MHz, Acetone-d_6)
δ 164.3 (d, *J* = 245 Hz), 156.6, 150.5, 137.8, 136.5, 129.5 (d, *J* = 8.8 Hz), 123.1, 120.6,

[4] Knapp, D. M.; Gillis, E. P.; Burke, M. D. *J. Am. Chem. Soc.*, **2009**, *131*, 6961-6963.

116.2 (d, $J = 22$ Hz).

HRMS (ESI+)

Calculated for $C_{11}H_9NF$ $(M+H)^+$:	174.0719
Found:	174.0717

1-(4-(pyridin-2-yl)phenyl)ethanone (4a) [Table 2, entry 9]. The general procedure was followed using MIDA boronate **1a** (351 mg, 1.50 mmol), chloride **3a** (130 µL, 1.00 mmol) and running the reaction at 80 ˚C for 24 h. The crude product was subjected to silica gel chromatography (EtOAc:Hexanes 50:50) to afford **4a** as an off-white crystalline solid (164 mg, 83%). Characterization was consistent with literature.[4]

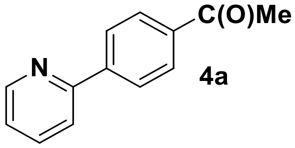

4a

2,5-dimethyl-3-(pyridin-2-yl)pyrazine (4k) [Table 2, entry 10]. The general procedure was followed using MIDA boronate **1a** (359 mg, 1.53 mmol) and chloride **3k** (120 µL, 1.00 mmol). The crude product was subjected twice to silica gel chromatography (first Run, MeCN:Et_2O 10:90; second Run, Acetone:Hexanes (with 1% Triethylamine) 65:35) to afford **4k** as a brown oil (141 mg, 77%). Characterization was consistent with literature.[4]

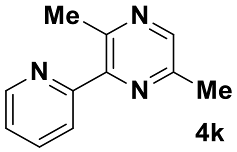

4k

2-(pyridin-2-yl)quinoxaline (4l) [Table 2, entry 11]. The general procedure was followed using MIDA boronate **1a** (352 mg, 1.50 mmol) and chloride **3l** (166 mg, 1.01 mmol). The crude product was subjected to Florisil chromatography (EtOAc:Hexanes 40:60) to afford **4l** as an off-white crystalline solid (166 mg, 80%). Characterization was consistent with literature.[4]

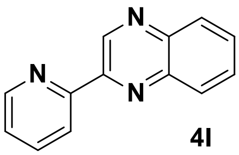

4l

5-(pyridin-2-yl)-1H-indole (4m) [Table 2, entry 12]. The general procedure was followed using MIDA boronate **1a** (351 mg, 1.50 mmol) and chloride **3m** (151 mg, 1.00 mmol. The crude product was subjected to Florisil chromatography (EtOAc:Hexanes 50:50) and silica gel chromatography (EtOAc:Hexanes 25:75) to afford **4m** as a brown crystalline solid (120 mg, 62%).

---

[4] Knapp, D. M.; Gillis, E. P.; Burke, M. D. *J. Am. Chem. Soc.*, **2009**, *131*, 6961-6963.
[4] Knapp, D. M.; Gillis, E. P.; Burke, M. D. *J. Am. Chem. Soc.*, **2009**, *131*, 6961-6963.

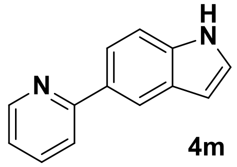

4m

TLC (EtOAc:Hexanes 50:50)

R_f = 0.36, visualized by UV (λ = 254 nm)

^{1}H-NMR (500 MHz, Acetone-d_6)

δ 10.36 (s, 1H), 8.61 (d, *J* = 3.5 Hz, 1H), 8.36 (s, 1H), 7.93 (ddd, *J* = 1.5, 8.5, 16.5 Hz, 2H), 7.79 (td, *J* = 1.5, 7.0 Hz, 1H), 7.50 (d, *J* = 8.5 Hz, 1H), 7.37 (t, *J* = 2.5 Hz, 1H), 7.21 (ddd, *J* = 1.0, 5.0, 7.5 Hz, 1H), 6.56 (d, *J* = 2.0 Hz, 1H).

^{13}C-NMR (125 MHz, Acetone-d_6)

δ 159.2, 150.1, 137.8, 137.4, 131.6, 129.3, 126.5, 121.8, 121.4, 120.4, 119.8, 112.2, 103.2

HRMS (ESI+)

Calculated for $C_{13}H_{11}N_2$ $(M+H)^+$:	195.0922
Found:	195.0918

2-(pyridin-2-yl)quinoxaline (4n) [Table 2, entry 13]. The general procedure was followed using MIDA boronate **1a** (350 mg, 1.50 mmol) and chloride **3n** (168 mg, 1.02 mmol). The crude product was subjected to silica gel chromatography (EtOAc:Hexanes 70:30) and Florisil chromatography (EtOAc:Hexanes 30:70 → 100:0) to afford **4n** as an amber oil (135 mg, 64%). Characterization was consistent with literature.[4]

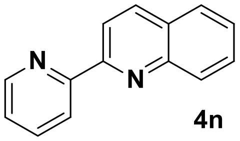

4n

2-methyl-5-(pyridin-2-yl)benzo[d]oxazole (4o) [Table 2, Entry 14]. The general procedure was followed using MIDA boronate **1a** (360 mg, 1.54 mmol) and chloride **3o** (168 mg, 1.00 mmol. The crude product was subjected to silica gel chromatography (EtOAc:Hexanes 5:95 → 75:25) to afford **4o** as an off-white crystalline solid (173 mg, 82%).

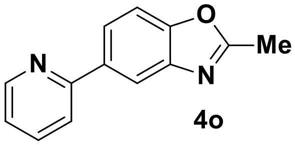

4o

TLC (EtOAc:Hexanes 75:25)

R_f = 0.38, visualized by UV (λ = 254 nm)

[4] Knapp, D. M.; Gillis, E. P.; Burke, M. D. *J. Am. Chem. Soc.*, **2009**, *131*, 6961-6963.

^{1}H-NMR (500 MHz, CD_3CN)
δ 8.65 (d, *J* = 4.0 Hz, 1H), 8.26 (d, *J* = 1.5 Hz, 1H), 8.06 (dd, *J* = 1.5, 8.5 Hz, 1H), 7.89 (dt, *J* = 1.0, 8.0 Hz, 1H), 7.83 (td, *J* = 2.0, 7.5 Hz, 1H), 7.61 (d, *J* = 8.5 Hz, 1H), 7.29 (ddd, *J* = 1.0, 5.0, 7.5 Hz, 1H), 2.60 (s, 3H).

^{13}C-NMR (125 MHz, Acetone-d_6)
δ 165.4, 157.3, 152.4, 150.4, 143.3, 137.7, 136.7, 124.3, 122.9, 120.9, 118.3, 110.8, 14.3

HRMS (ESI+)
Calculated for $C_{13}H_{11}N_2O$ $(M+H)^+$: 211.0871
Found: 211.0874

X. Table 3

2-(4-(tertbutoxy)phenyl)-6-methylpyridine (4p) [Table 3, entry 1]. The general procedure was followed using MIDA boronate **1b** (376 mg, 1.52 mmol) and chloride **3b** (175 µL, 1.00 mmol). The crude product was subjected to Florisil chromatography (EtOAc:Hexanes 10:90)**.** The mixed fractions were reconstituted and subject to a second Florisil column (EtOAc:Hexanes 10:90) to afford **4p** as an amber solid (174 mg, 72%).

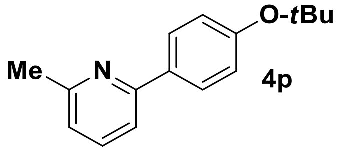

4p

TLC (EtOAc: Hexanes 10:90)
R_f = 0.36, visualized by UV (λ = 254 nm)

^{1}H-NMR (400 MHz, Acetone-d_6)
δ 8.03 (d, *J* = 8.4 Hz, 2H), 7.67 (m, 2H), 7.13 (d, *J* = 7.4 Hz, 1H), 7.07 (d, *J* = 8.8 Hz, 2H), 2.53 (s, 3H), 1.37 (s, 9H).

^{13}C-NMR (125 MHz, Acetone-d_6)
δ 158.7, 157.4, 156.5, 137.8, 135.0, 128.1, 124.4, 121.8, 117.2, 78.9, 29.1, 24.7

HRMS (ESI+)
Calculated for $C_{16}H_{20}NO$ $(M+H)^+$: 242.1545
Found: 242.1539

2-(4-(tertbutoxy)phenyl)-5-methylpyridine (4q) [Table 3, entry 2]. The general procedure was followed using MIDA boronate **1c** (375 mg, 1.51 mmol) and chloride **3b** (175 µL, 1.00 mmol). The crude product was subjected to Florisil chromatography (EtOAc:Hexanes 10:90) to afford 4q as an amber solid (197 mg, 81%).

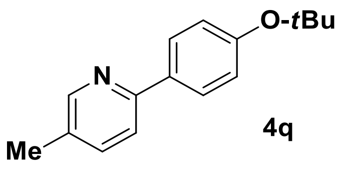

4q

TLC (EtOAc: Hexanes 20:80)
R_f = 0.36, visualized by UV (λ = 254 nm)

^{1}H-NMR (400 MHz, Acetone-d_6)
δ 8.46 (s, 1H), 8.01 (d, *J* = 8.8 Hz, 2H), 7.76 (d, *J* = 8.0 Hz, 1H), 7.63 (dd, *J* = 1.8, 8.4 Hz, 1H), 7.07 (d, *J* = 8.8 Hz, 2H), 2.33 (s, 3H), 1.36 (s, 9H).

^{13}C-NMR (125 MHz, Acetone-d_6)
δ 157.2, 154.6, 150.6, 137.9, 134.9, 131.8, 127.8, 124.4, 119.7, 78.8, 29.1, 18.0

HRMS (ESI+)

Calculated for $C_{16}H_{20}NO$ $(M+H)^+$:	242.1545
Found:	242.1539

2-(4-(tertbutoxy)phenyl)-4-methylpyridine (4r) [Table 3, entry 3]. The general procedure was followed using MIDA boronate **1d** (374 mg, 1.51 mmol) and chloride **3b** (175 µL, 1.00 mmol). The crude product was subjected to Florisil chromatography (EtOAc:Hexanes 10:90) to afford **4r** as an amber oil (197 mg, 81%).

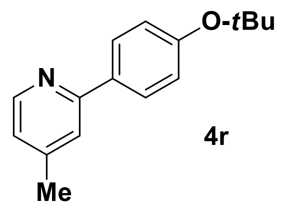

4r

TLC (EtOAc: Hexanes 10:90)
R_f = 0.22, visualized by UV (λ = 254 nm)

^{1}H-NMR (400 MHz, Acetone-d_6)
δ 8.46 (d, *J* = 5.0 Hz, 1H), 8.03 (d, *J* = 8.8 Hz, 2H), 7.71 (s, 1H), 7.08 (m, 3H), 2.39 (s, 3H), 1.36 (s, 9H).

^{13}C-NMR (125 MHz, Acetone-d_6)
δ 157.5, 157.2, 150.0, 148.4, 135.0, 128.1, 124.4, 123.4, 121.1, 78.9, 29.1, 21.0

HRMS (ESI+)

Calculated for $C_{16}H_{20}NO$ $(M+H)^+$:	242.1545
Found:	242.1539

2-(2,4-dimethoxyphenyl)-6-methoxypyridine (4s) [Table 3, entry 4]. The general procedure was followed using MIDA boronate **1e** (340 mg, 1.29 mmol) and chloride **3h** (127.5 µL, 0.86 mmol). The crude product was subjected to Florisil chromatography (EtOAc:Hexanes 10:90). The mixed fractions were reconstituted and subjected to silica gel chromatography (EtOAc:Hexanes 10:90) to afford **4s** as an amber oil (162 mg, 77%).

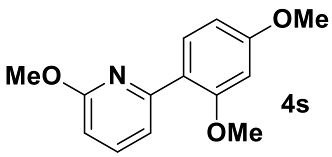

4s

TLC (EtOAc: Hexanes 10:90)

R_f = 0.3, visualized by UV (λ = 254 nm)

^{1}H-NMR (400 MHz, Acetone-d_6)

δ 8.01 (d, *J* = 8.4 Hz, 1H), 7.61 (dt, *J* = 8.0, 16.0 Hz, 2H), 6.63 (m, 3H), 3.93 (s, 3H), 3.90 (s, 3H), 3.85 (s, 3H).

^{13}C-NMR (125 MHz, Acetone-d_6)

δ 164.1, 162.3, 159.5, 153.5, 139.4, 132.5, 121.8, 117.9, 108.6, 106.1, 99.3, 55.9, 55.6, 53.1

HRMS (ESI+)

Calculated for $C_{14}H_{16}NO_3$ $(M+H)^+$:	246.1130
Found:	246.1130

2-(4-(tertbutoxy)phenyl)-6-trifluoromethylpyridine (4t) [Table 3, entry 5]. The general procedure was followed using MIDA boronate **1f** (454 mg, 1.50 mmol) and chloride **3b** (175 µL, 1.00 mmol). The crude product was subjected to Florisil chromatography (EtOAc:Hexanes 5:95). The mixed fractions were reconstituted and subject to a second Florisil column (EtOAc:Hexanes 5:95) to afford **4t** as an amber solid (258 mg, 87%).

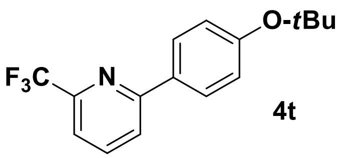

4t

TLC (EtOAc: Hexanes 5:95)

R_f = 0.3, visualized by UV (λ = 254 nm)

^{1}H-NMR (400 MHz, Acetone-d_6)

δ 8.12 (m, 4H), 7.74 (d, *J* = 7.4 Hz, 1H), 7.14 (d, *J* = 8.8 Hz, 2H), 1.39 (s, 9H).

^{13}C-NMR (125 MHz, Acetone-d_6)

δ 158.6, 158.0, 148.2 (q, *J* = 34 Hz), 139.7, 132.9, 128.6, 124.4, 123.4, 122.7 (q, J = 272

Hz), 119.0 (d, *J* = 3 Hz), 79.3, 29.1

HRMS (ESI+)
Calculated for $C_{16}H_{17}F_3NO$ $(M+H)^+$: 296.1262
Found: 296.1254

2-(4-methoxyphenyl)-5-trifluoromethylpyridine (4u) [Table 3, entry 6]. The general procedure was followed using MIDA boronate **1g** (229 mg, 0.76 mmol) and chloride **3c** (61 µL, 0.5 mmol). The crude product was subjected to Florisil chromatography (Et_2O:Hexanes 5:95). The mixed fractions were recombined and subject to a second Florisil column (Et_2O:Hexanes 5:95) to afford **4u** as an off white crystalline solid (99 mg, 78%).

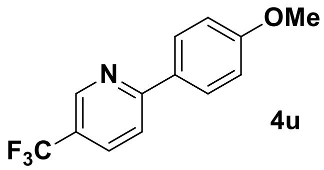

4u

TLC (Et_2O: Hexanes 10:90)
R_f = 0.39, visualized by UV (λ = 254 nm)

^{1}H-NMR (500 MHz, Acetone-d_6)
δ 8.92 (s, 1H), 8.17 (m, 3H), 8.08 (d, *J* = 8.5 Hz, 1H), 7.07 (d, *J* = 8.5 Hz, 2H), 3.88 (s, 3H).

^{13}C-NMR (125 MHz, Acetone-d_6)
δ 162.5, 161.0, 147.0 (d, *J* = 4 Hz), 134.9 (d, *J* = 4 Hz), 131.0, 129.5, 125.1 (q, *J* = 269 Hz), 124.3 (q, *J* = 32 Hz), 115.1, 55.7

HRMS (ESI+)
Calculated for $C_{13}H_{11}F_3NO$ $(M+H)^+$: 254.0793
Found: 254.0791

2-(4-(tertbutoxy)phenyl)-4-trifluoromethylpyridine (4v) [Table 3, entry 7]. The general procedure was followed using MIDA boronate **1h** (464 mg, 1.54 mmol) and chloride **3b** (175 µL, 1.00 mmol). The crude product was subjected first to florisil chromatography (EtOAc:Hexanes 2.5:97.5) and then to silica gel chromatography (EtOAc:Hexanes 5:95) to afford **4v** as an amber oil (251 mg, 85%).

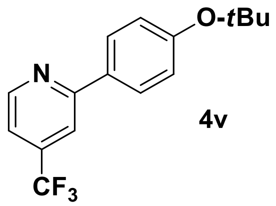

4v

TLC (EtOAc: Hexanes 10:90)

R_f = 0.4, visualized by UV (λ = 254 nm)

^{1}H-NMR (400 MHz, Acetone-d_6)

δ 8.89 (d, *J* = 5.4 Hz, 1H), 8.15 (d, *J* = 8.8 Hz, 3H), 7.59 (d, *J* = 5.4 Hz, 1H), 7.13 (d, *J* = 8.8 Hz, 2H), 1.39 (s, 9H).

^{13}C-NMR (125 MHz, Acetone-d_6)

δ 158.9, 158.6, 151.7, 139.3 (q, *J* = 33 Hz), 133.2, 128.6, 124.4, 124.2 (q, *J* = 271 Hz), 117.8 (q, *J* = 4 Hz), 115.7 (q, *J* = 4 Hz), 79.3, 29.1

HRMS (ESI+)

Calculated for $C_{16}H_{17}F_3NO$ (M+H)$^+$:	296.1262
Found:	296.1258

XI. Table 4

2-(p-tolyl)pyridine (4w) [Table 4, entry 1]. The general procedure was followed using MIDA boronate **1a** (355 mg, 1.52 mmol) and bromide **8a** (122.5 µL, 1.00 mmol. The crude product was subjected to silica gel chromatography (EtOAc:Hexanes 5:95 → 15:85) to afford **4w** as yellow liquid (140 mg, 83%).

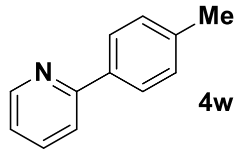

4w

TLC (EtOAc:Hexanes 17:83)

R_f = 0.45, visualized by UV (λ = 254 nm)

^{1}H-NMR (500 MHz, CD_3CN)

δ 8.62 (d, *J* = 4.5 Hz, 1H), 7.94 (d, *J* = 8.5 Hz, 2H), 7.80 (m, 2H), 7.29 (d, *J* = 8.0 Hz, 2H), 7.52 (ddd, *J* = 2.0, 5.0, 6.5 Hz, 1H), 2.38 (s, 3H).

^{13}C-NMR (125 MHz, Acetone-d_6)

δ 157.6, 150.3, 139.5, 137.5, 137.4, 130.1, 127.4, 122.8, 120.4, 21.2

HRMS (ESI+)

Calculated for $C_1H_{12}N$ (M+H)$^+$:	170.0970
Found:	170.0966

2-(4-methoxyphenyl)pyridine (4c) [Table 4, entry 2]. The general procedure was followed using MIDA boronate **1a** (356 mg, 1.52 mmol) and bromide **8b** (125 µL, 1.00 mmol). The crude product was subjected to silica gel chromatography (EtOAc:Hexanes 5:95 → 20:80) to afford **4c** as a orange solid (146 mg, 79%). Characterization was consistent with previous data.

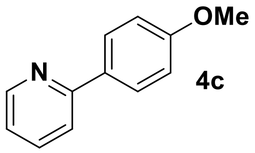

4c

2-(4-fluorophenyl)pyridine (4j) [Table 4, entry 3]. The general procedure was followed using MIDA boronate **1a** (358 mg, 1.53 mmol) and bromide **8c** (110 µL, 1.01 mmol). The crude product was subjected to silica gel chromatography (EtOAc:Hexanes 5:95 → 20:80) to afford **4j** as a pale yellow solid (145 mg, 84%). Characterization was consistent with previous data.

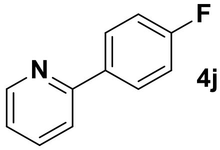

4j

5-(pyridin-2-yl)pyrimidine (4x) [Table 4, entry 4]. The general procedure was followed using MIDA boronate **1a** (353 mg, 1.51 mmol) and bromide **8d** (159 mg, 1.00 mmol). The crude product was subjected to silica gel chromatography (EtOAc) to afford **4x** as a white crystalline solid (74 mg, 47%).

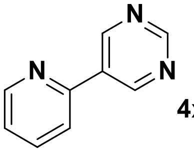

4x

TLC (EtOAc)
R_f = 0.27, visualized by UV (λ = 254 nm)

^{1}H-NMR (500 MHz, CD_3CN)
δ 9.34 (s, 2H), 9.18 (s, 1H), 8.72 (d, *J* = 4.5 Hz, 1H), 7.92 (m, 2H), 7.41 (ddd, *J* = 2.0, 5.0, 7.0 Hz, 1H).

^{13}C-NMR (125 MHz, Acetone-d_6)
δ 159.3, 155.7, 152.8, 151.1, 138.2, 133.1, 124.6, 121.5

HRMS (ESI+)

Calculated for $C_9H_8N_3$ $(M+H)^+$:	158.0718
Found:	158.0719

1-methyl-5-(pyridin-2-yl)-1H-indole (4y) [Table 4, entry 5]. The general procedure was followed using MIDA boronate **1a** (356 mg, 1.52 mmol) and bromide **8e** (213 mg, 1.01 mmol). The crude product was subjected to silica gel chromatography (EtOAc:Hexanes 5:95 →30:70) to afford **4y** as a brown crystalline solid (117 mg, 55%).

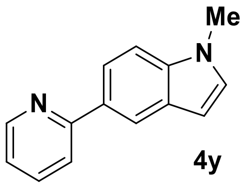

4y

TLC (EtOAc: Hexanes, 33:67)
R_f = 0.4, visualized by UV (λ = 254 nm)

^{1}H-NMR (500 MHz, Acetone-d_6)
δ 8.62 (d, *J* = 5.0 Hz, 1H), 8.34 (t, *J* = 1.0 Hz, 1H), 8.00 (dd, *J* = 2.0, 9.0 Hz, 1H), 7.92 (dt, *J* = 1.0, 8.0 Hz, 1H), 7.79 (m, 1H), 7.47 (d, *J* = 8.5 Hz, 1H), 7.26 (d, *J* = 3.0 Hz, 1H), 7.21 (ddd, *J* = 1.0, 5.0, 7.5 Hz, 1H), 6.52, (dd, *J* = 1.0, 3.0 Hz, 1H), 3.86 (s, 3H).

^{13}C-NMR (125 MHz, Acetone-d_6)
δ 159.1, 150.2, 138.3, 137.3, 131.5, 130.8, 129.8, 121.8, 121.3, 120.3, 120.0, 110.2, 102.3, 32.9

HRMS (ESI+)

Calculated for $C_{14}H_{13}N_2$ $(M+H)^+$:	209.1079
Found:	209.1079

2-(p-tolyl)pyridine (4w) [Table 4, entry 6]. The general procedure was followed using MIDA boronate **1a** (354 mg, 1.52 mmol) and iodide **8f** (130 µL, 1.02 mmol. The crude product was subjected to silica gel chromatography (EtOAc:Hexanes 5:95 → 15:85) to afford **4w** as yellow liquid (129 mg, 75%). Characterization was consistent with previous data.

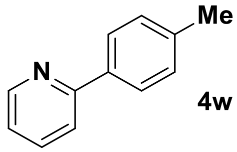

4w

2-(4-methoxyphenyl)pyridine (4c) [Table 4, entry 7]. The general procedure was followed using MIDA boronate **1a** (355 mg, 1.52 mmol) and iodide **8g** (235 mg, 1.00 mmol). The crude product was subjected to silica gel chromatography (EtOAc:Hexanes 5:95 → 20:80) to afford **4c** as a orange solid (139 mg, 75%). Characterization was consistent with previous data.

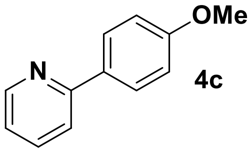

4c

2-(4-fluorophenyl)pyridine (4j) [Table 4, entry 8]. The general procedure was followed using MIDA boronate **1a** (360 mg, 1.54 mmol) and iodide **8h** (115 µL, 0.995 mmol). The crude product was subjected to silica gel chromatography (EtOAc:Hexanes 5:95 → 20:80) to afford **4j** as a pale yellow solid (139 mg, 81%). Characterization was consistent with previous data.

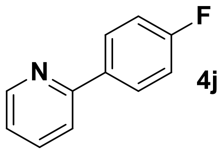
4j

tert-butyl 5-(pyridin-2-yl)-1H-indole-1-carboxylate (4z) [Table 4, entry 9]. The general procedure was followed using MIDA boronate **1a** (355 mg, 1.52 mmol), iodide **8i** (346 mg, 1.01 mmol), and heating the reaction to 80 ˚C for 24 h. The crude product was subjected to silica gel chromatography (EtOAc:Hexanes 5:95 → 20:80) to afford **4z** as a brown oil (199 mg, 67%).

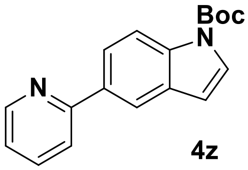
4z

TLC (EtOAc: Hexanes 20:80)
R_f = 0.42, visualized by UV (λ = 254 nm)

^{1}H-NMR (500 MHz, CD_3CN)
δ 8.64 (d, *J* = 5.0 Hz, 1H), 8.28 (d, *J* = 1.5 Hz, 1H), 8.20 (d, *J* = 8.5 Hz, 1H), 8.04 (dd, *J* = 2.0, 8.5 Hz, 1H), 7.89 (dt, *J* = 1.0, 8.0 Hz, 1H), 7.83 (td, *J* = 1.5, 8.0 Hz, 1H), 7.67 (d, *J* = 3.5 Hz, 1H), 7.27 (ddd, *J* = 1.5, 5.0, 7.5 Hz, 1H), 6.71 (d, *J* = 3.5 Hz, 1H), 1.66 (s, 9H).

^{13}C-NMR (125 MHz, Acetone-d_6)
δ 157.9, 150.3, 150.1, 137.5, 136.5, 134.9, 131.8, 127.4, 123.8, 122.6, 120.7, 120.2, 115.8, 108.5, 84.5, 28.2

HRMS (ESI+)
Calculated for $C_{18}H_{19}N_2O_2$ $(M+H)^+$: 295.1447
Found: 295.1446

2-(p-tolyl)pyridine (4q) [Table 4, entry 10]. The general procedure was followed using MIDA boronate **1a** (357 mg, 1.53 mmol) and triflate **8j** (180 µL, 1.01 mmol. The crude product was subjected to silica gel chromatography (EtOAc:Hexanes 5:95 → 15:85) to afford **4q** as yellow liquid (138 mg, 81%). Characterization was consistent with previous data.

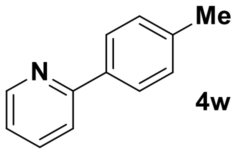
4w

2-(3,5-dimethoxyphenyl)pyridine (4aa) [Table 4, entry 11]. The general procedure was followed using MIDA boronate **1a** (352 mg, 1.50 mmol) and triflate **8k** (202.5 µL, 1.00 mmol). The crude product was subjected to silica gel chromatography (EtOAc:Hexanes 5:95 → 30:70) to afford **4aa** as an amber oil (187 mg, 87%).

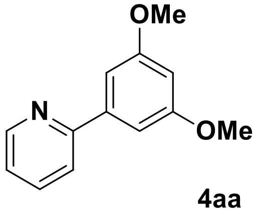

4aa

TLC (EtOAc: Hexanes 20:80)
R_f = 0.21, visualized by UV (λ = 254 nm)

^{1}H-NMR (500 MHz, CD_3CN)
δ 8.63 (d, *J* = 4.0 Hz, 1H), 7.83 (m, 2H), 7.29 (t, *J* = 8.5 Hz, 1H), 7.20 (d, *J* = 2.0 Hz, 2H), 6.55 (t, *J* = 2.5 Hz, 1H), 3.84 (s, 6H).

^{13}C-NMR (125 MHz, Acetone-d_6)
δ 162.1, 157.2, 150.2, 142.2, 137.6, 123.3, 121.1, 105.4, 101.7, 55.6

HRMS (ESI+)

Calculated for $C_{13}H_{14}NO_2$ $(M+H)^+$:	216.1025
Found:	216.1023

1-(4-(pyridin-2-yl)phenyl)ethanone (4a) [Table 4, entry 12]. The general procedure was followed using MIDA boronate **1a** (353 mg, 1.51 mmol), triflate **8l** (190 µL, 1.00 mmol) and running the reaction at 80 ˚C for 24 h. The crude product was subjected to Florisil chromatography (EtOAc:Hexanes 5:95 → 50:50) to afford **4a** as an off-white crystalline solid (103 mg, 52%). Characterization was consistent with literature.[4]

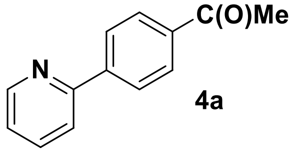

4a

2-(naphthalen-2-yl)pyridine (4bb) [Table 4, entry 13]. The general procedure was followed using MIDA boronate **1a** (353 mg, 1.51 mmol) and triflate **8m** (276 mg, 1.00 mmol). The crude product was subjected to silica gel chromatography (EtOAc:Hexanes 0:100 → 10:90) to afford **4bb** as an off-white crystalline solid (185 mg, 90%).

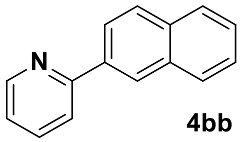

4bb

TLC (EtOAc: Hexanes 10:90)

R_f = 0.27, visualized by UV (λ = 254 nm)

^{1}H-NMR (400 MHz, Acetone-d_6)

δ 8.71 (d, *J* = 4.0 Hz, 1H), 8.65 (s, 1H), 8.30 (dd, *J* = 2, 7.4 Hz, 1H), 8.10 (d, *J* = 8.0 Hz, 1H), 8.02 (m, 2H), 7.92 (m, 2H), 7.54 (m, 2H), 7.35 (ddd, *J* = 1.4, 5.0, 7.4 Hz, 1H).

^{13}C-NMR (125 MHz, Acetone-d_6)

δ 157.4, 150.5, 137.7, 137.5, 134.6, 134.4, 129.5, 129.0, 128.4, 127.4, 127.1, 126.8, 125.3, 123.2, 121.1

HRMS (ESI+)

Calculated for $C_{15}H_{12}N$ $(M+H)^+$:	206.0970
Found:	206.0967

6-(pyridin-2-yl)quinoline (4cc) [Table 4, entry 14]. The general procedure was followed using MIDA boronate **1a** (350 mg, 1.50 mmol) and triflate **8n** (277 mg, 1.00 mmol). The crude product was subjected to Florisil chromatography (EtOAc:Hexanes 5:95 → 70:30). The appropriate fractions were concentrated and transferred washing with 20 mL Et_2O to a 60 mL separatory funnel. The solution was diluted with 5 mL 2N HCl and shaken. The organic layer was separated and discarded. The aqueous layer was extracted w/ 20 mL Et_2O and the organic layer was again discarded. The aqueous layer was diluted with 10 mL 2N NaOH and shaken. The aqueous layer was then extracted twice with 20 mL Et_2O. The organic fractions were combined and dried with Na_2SO_4. The solution was filtered and the filtrate was concentrated to afford **4cc** as an off-white crystalline solid (166 mg, 80%).

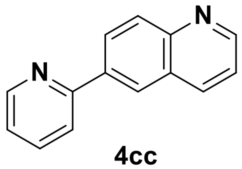

4cc

TLC (EtOAc: Hexanes 30:70)

R_f = 0.25, visualized by UV (λ = 254 nm)

^{1}H-NMR (500 MHz, Acetone-d_6)

δ 8.92 (dd, *J* = 1.5, 4.0 Hz, 1H), 8.74 (d, *J* = 5.0 Hz, 1H), 8.69 (d, *J* = 2.0 Hz, 1H), 8.55 (dd, *J* = 2.0, 8.5 Hz, 1H), 8.43 (d, *J* = 8 Hz, 1H), 8.13 (m, 2H), 7.93 (td, *J* = 1.5, 7.5 Hz, 1H), 7.54 (dd, *J* = 4.0, 8.5 Hz, 1H), 7.38 (ddd, *J* = 1.0, 5.0, 7.5 Hz, 1H).

^{13}C-NMR (125 MHz, Acetone-d_6)

δ 156.6, 151.7, 150.5, 149.4, 137.8, 137.2, 130.4, 129.0, 128.6, 126.7, 123.4, 122.4, 121.2, 121.2

HRMS (ESI+)

Calculated for $C_{14}H_{11}N_2$ $(M+H)^+$:	207.0922

Found: 207.0923

XII. Cross-coupling of 2-pyridyl MIDA boronate without a glovebox

This procedure was used for the coupling of 2-pyridyl MIDA boronate **1a** to 4-chloro-tertbutoxybenzene **3b**.

Under air to a 40 mL I-CHEM vial equipped with PTFE coated stir bar was added $Cu(OAc)_2$ (91 mg, 0.50 mmol), 2-pyridyl MIDA boronate **1a** (351 mg, 1.50 mmol), Xphos Palladacycle, chloro(2-dicyclohexylphosphino-2',4',6'-tri-i-propyl-1,1'-biphenyl)[2-(2-aminoethyl)phenyl] palladium(II) methyl-t-butylether adduct, (42 mg, 0.057 mmol) and K_3PO_4 (1.061g, 5.0 mmol). To the vial was added a PTFE coated stirbar. The vial was sealed with a septum cap and back-filled with argon using a 22.5 gauge needle with a gas adapter connecting to a schlenk line. To the vial was added DMF (8 mL), 1-(tert-butoxy)-4-chlorobenzene **3b** (174 µL, 1.00 mmol), and diethanolamine (96 µL, 1.00 mmol) by syringe using 22 or greater gauge needles. The argon line was then removed from the septum and the vial was heated to 100˚C with stirring for 24 h. The reaction was then allowed to cool to room temperature over 0.5 h. The reaction was transferred to a 60 mL separatory funnel, diluted with 10 mL of 2N HCl and shaken. The reaction was then diluted with 10 mL 2N 2N NaOH and shaken. The resulting aqueous solution was extracted three times with 10 mL of diethyl ether. The organic layers were combined and washed with 10 mL of brine. The organic layers were dried using Na_2SO_4, filtered, and concentrated *in vacuo*. The resulting residue was adsorbed onto Celite and subjected to Florisil chromatography (EtOAc:Hexanes 5:95 → 20:80) to afford **4b** as a pale yellow solid. This procedure was performed twice obtaining yields of 200 mg (88%), and 197 mg (87%).

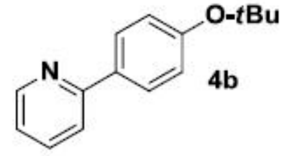

4b

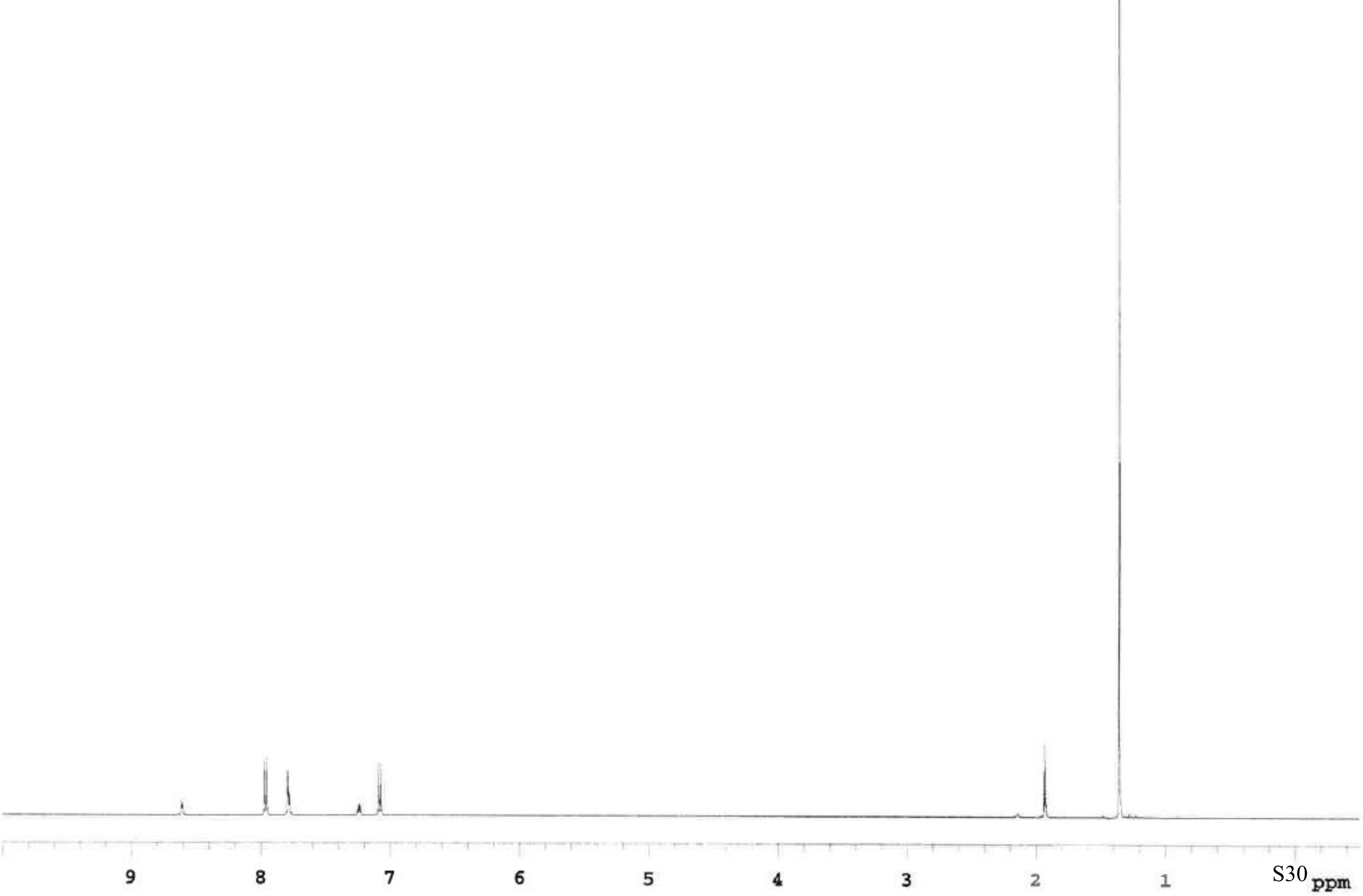

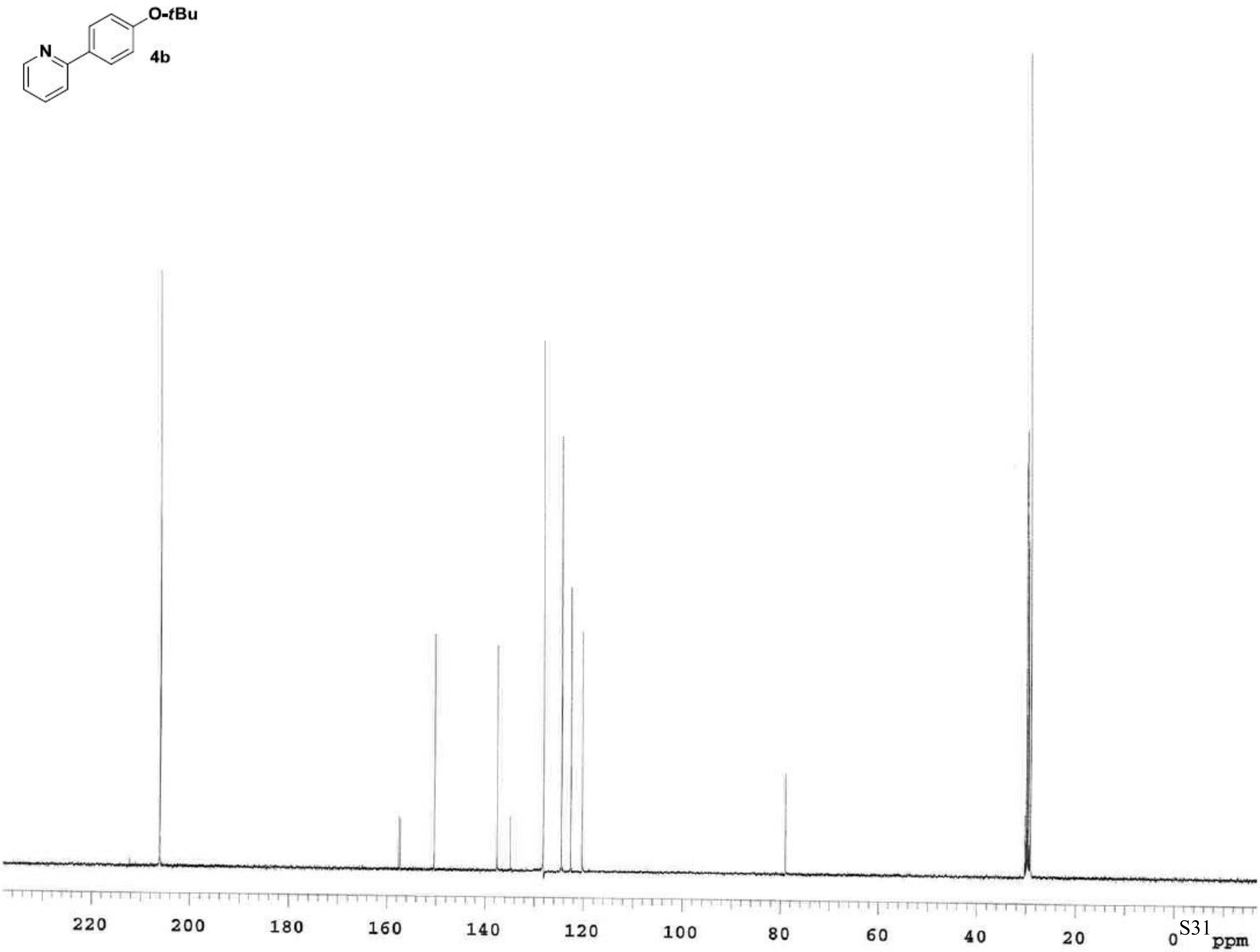
O-tBu
N
4b
220
200
180
160
140
120
100
80
60
40
20
0
ppm

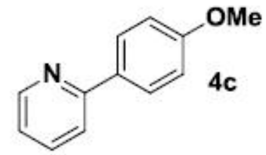

4c

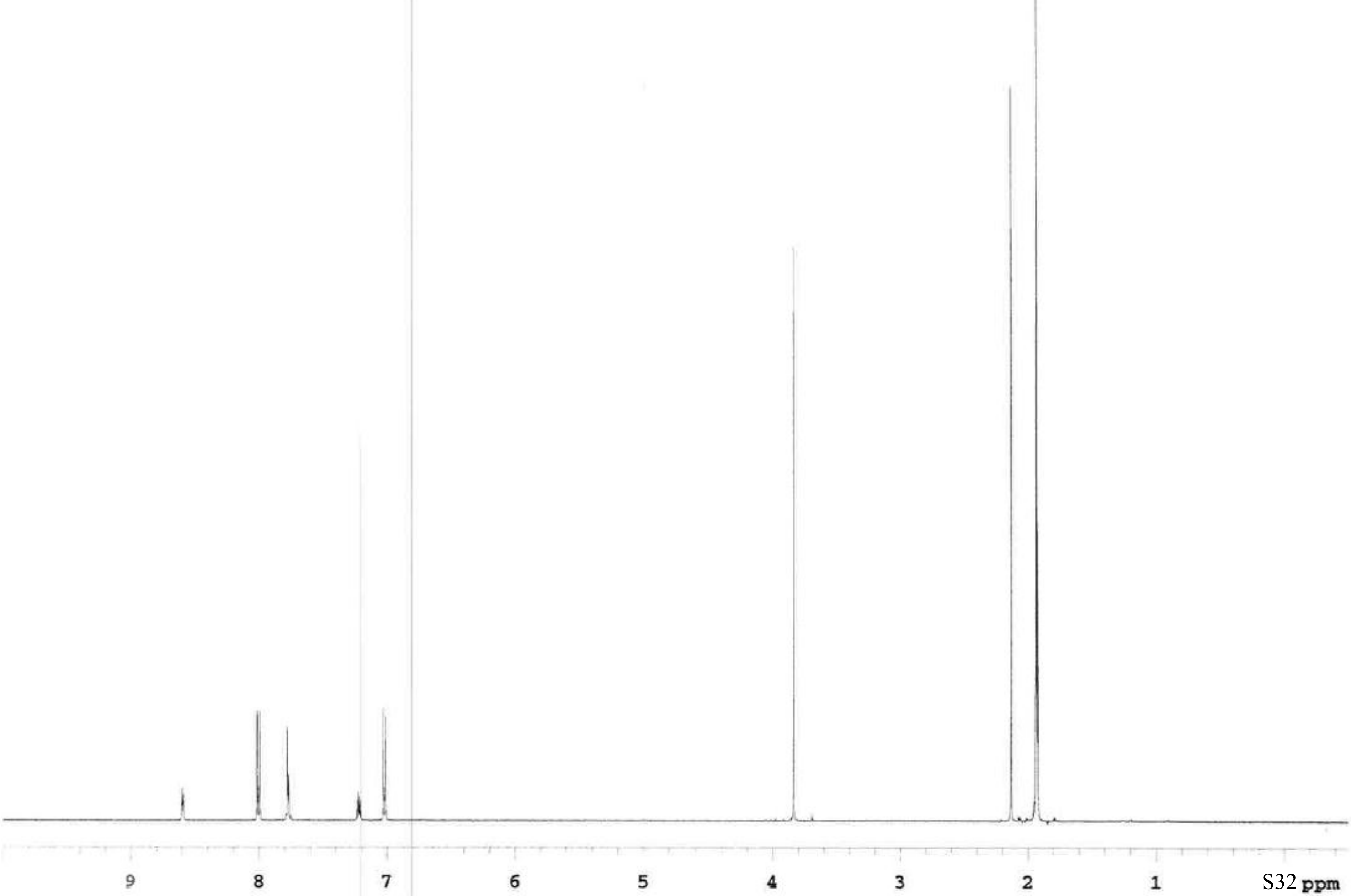

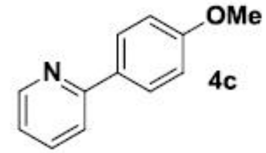

4c

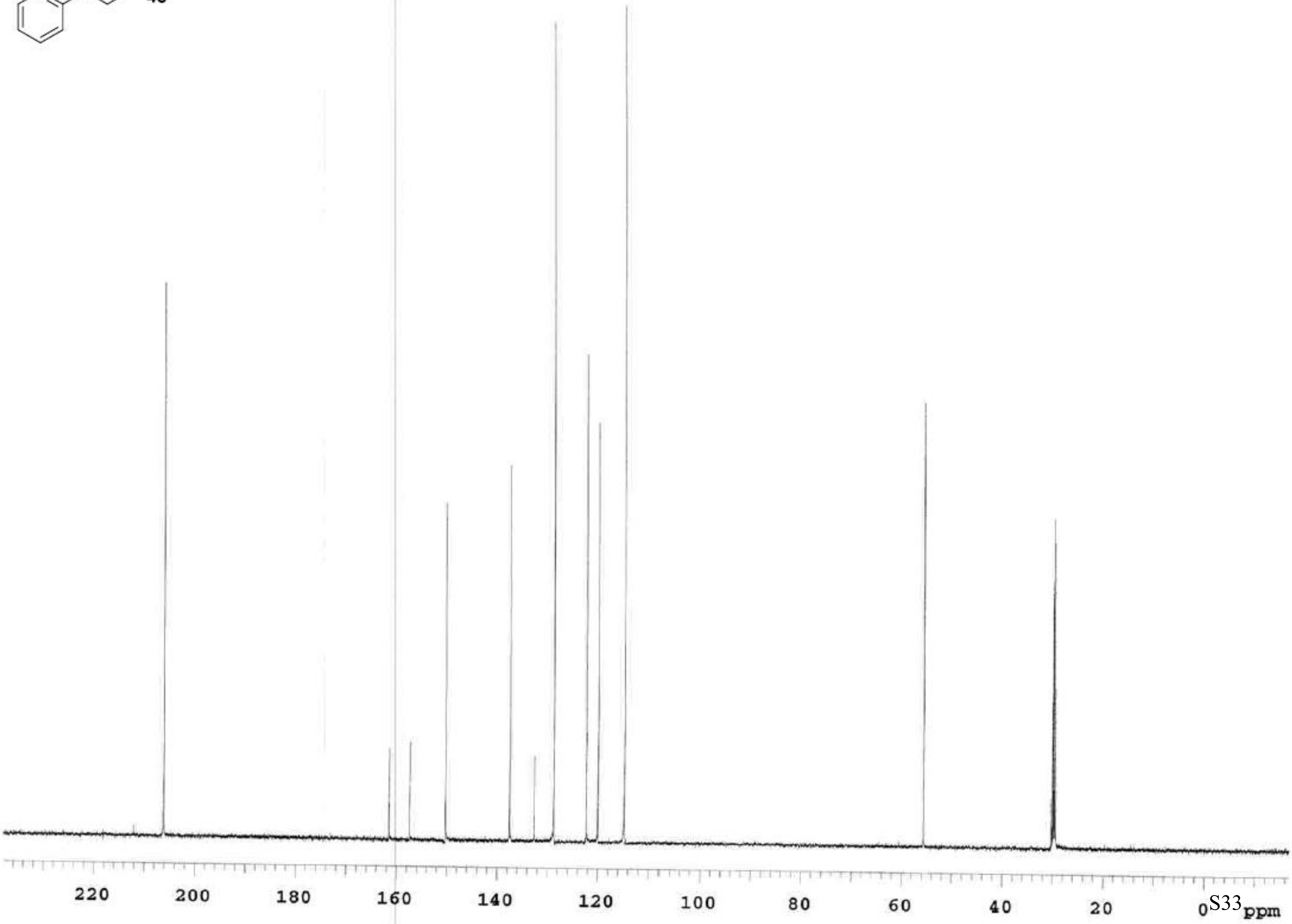

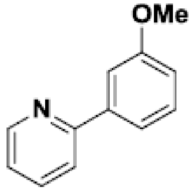

4d

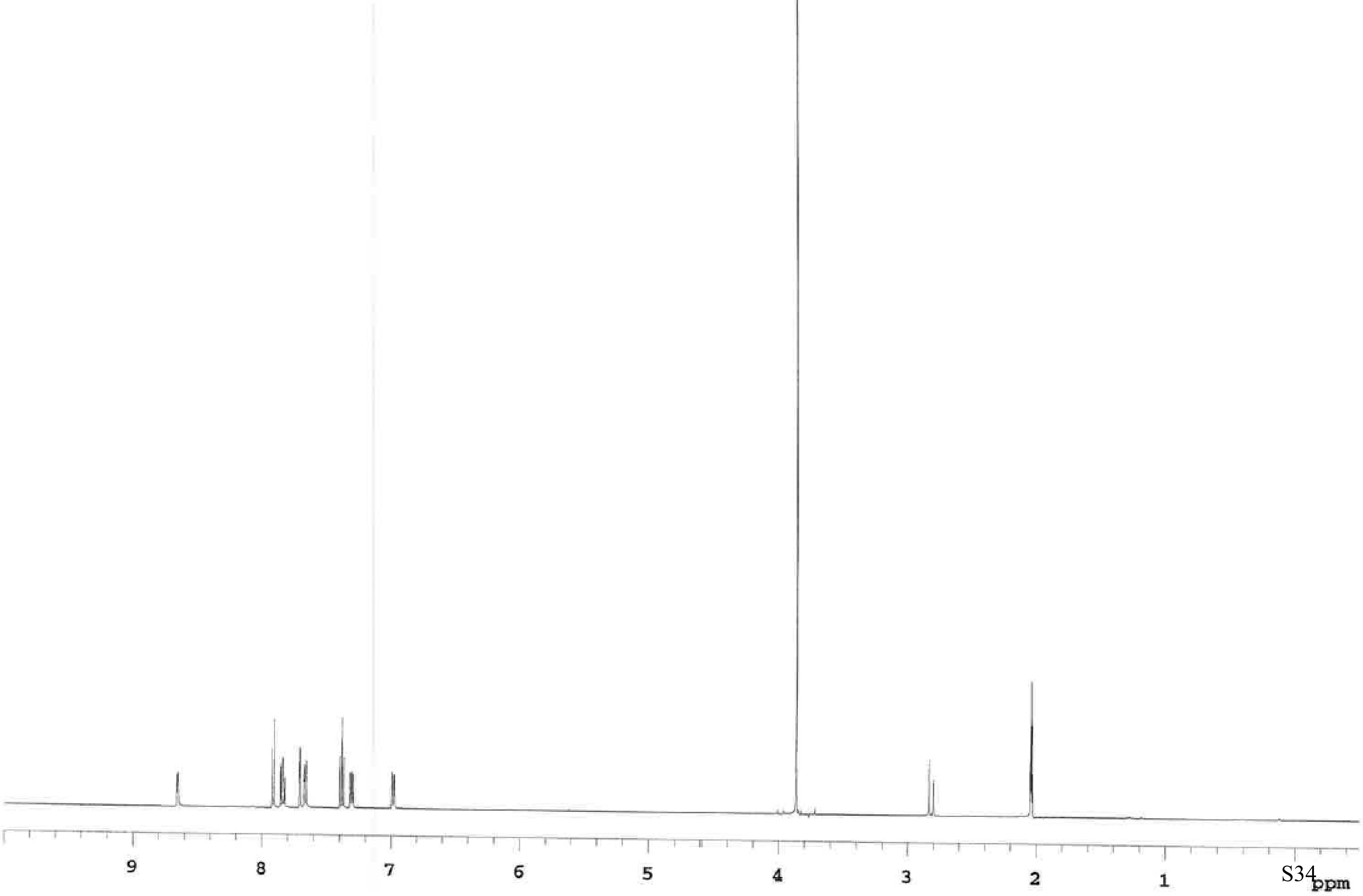

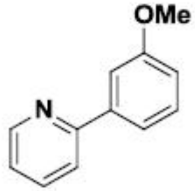

4d

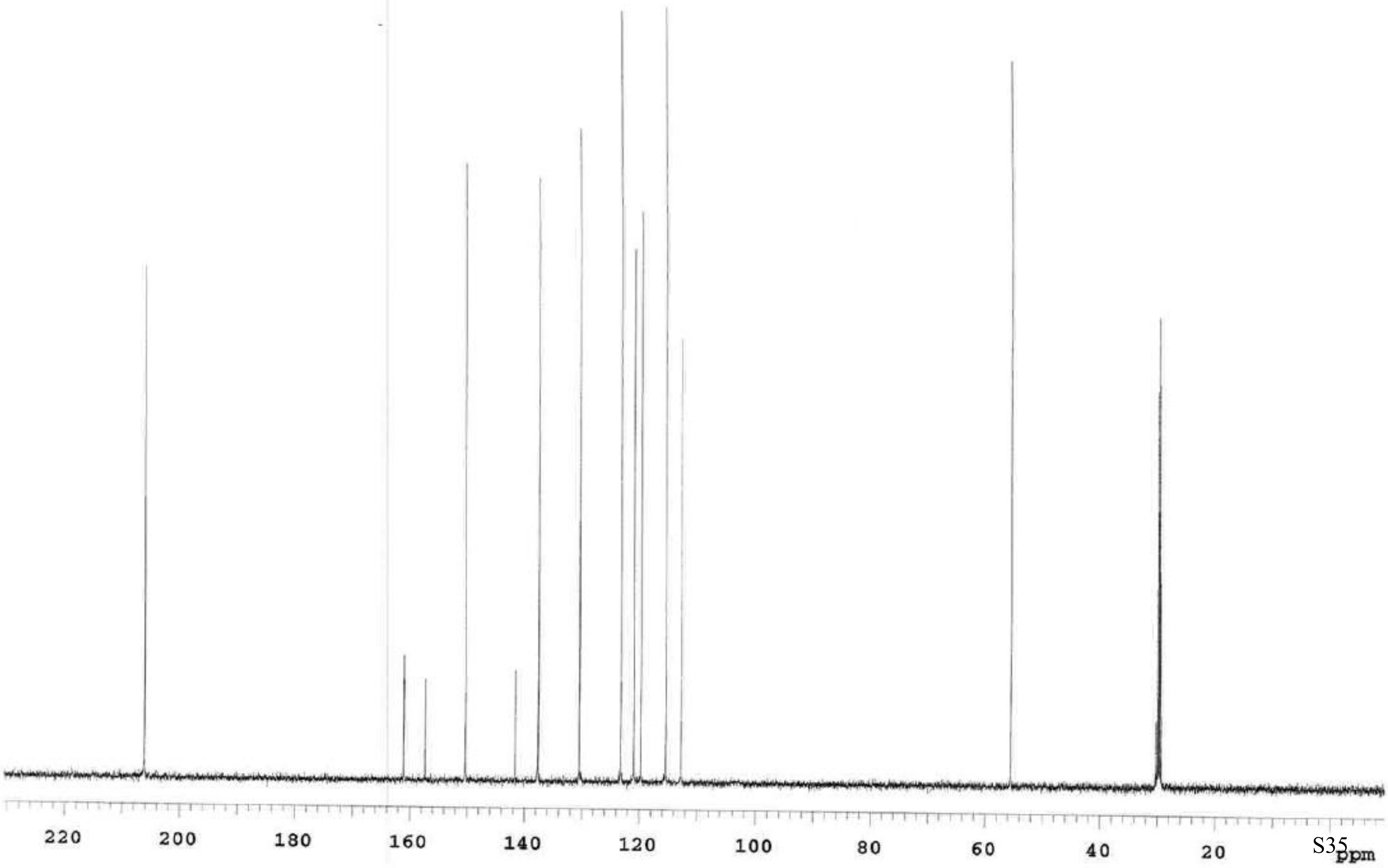

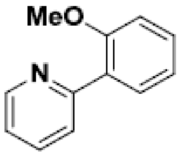

4e

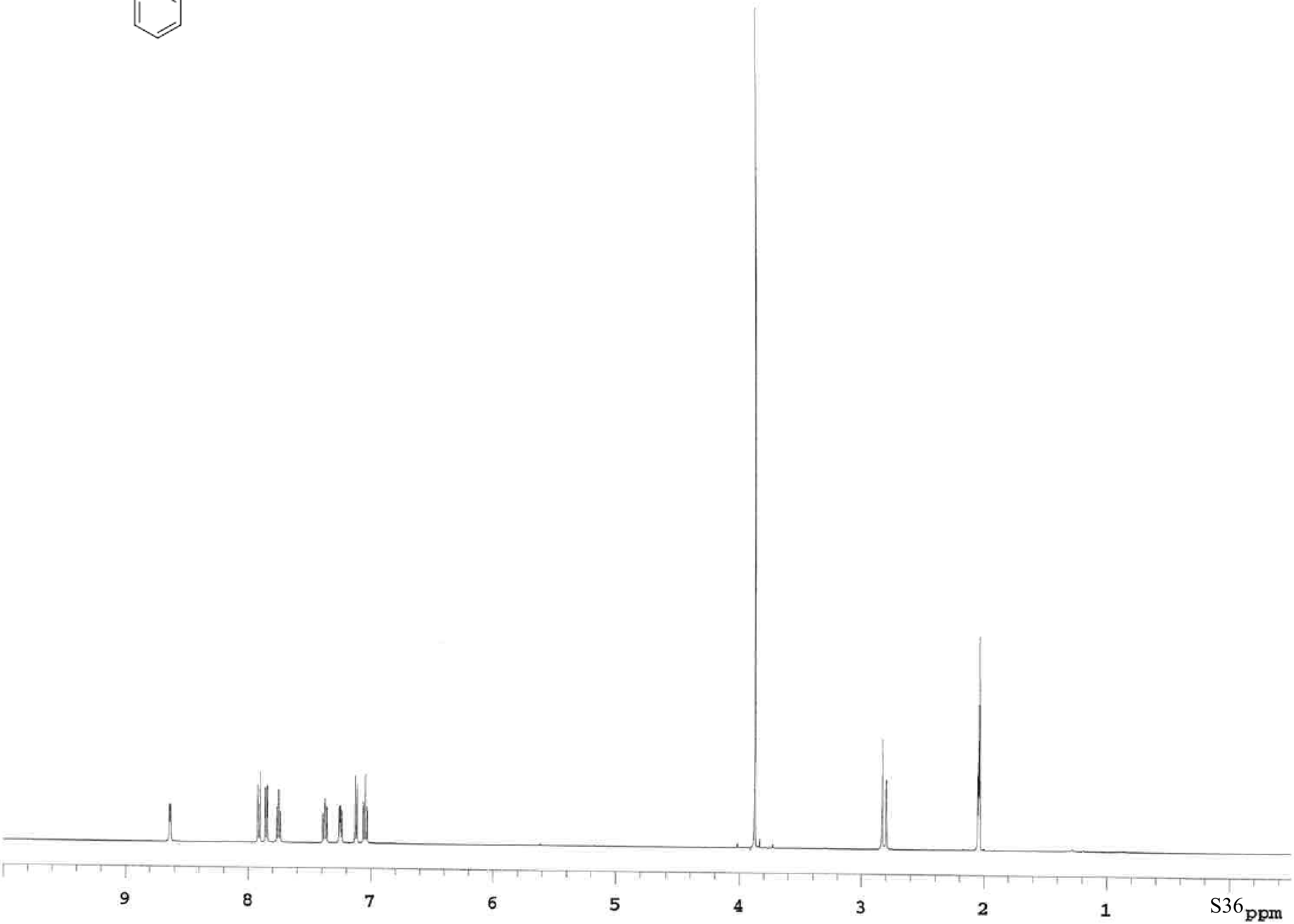

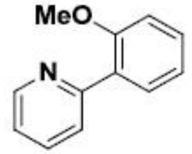

4e

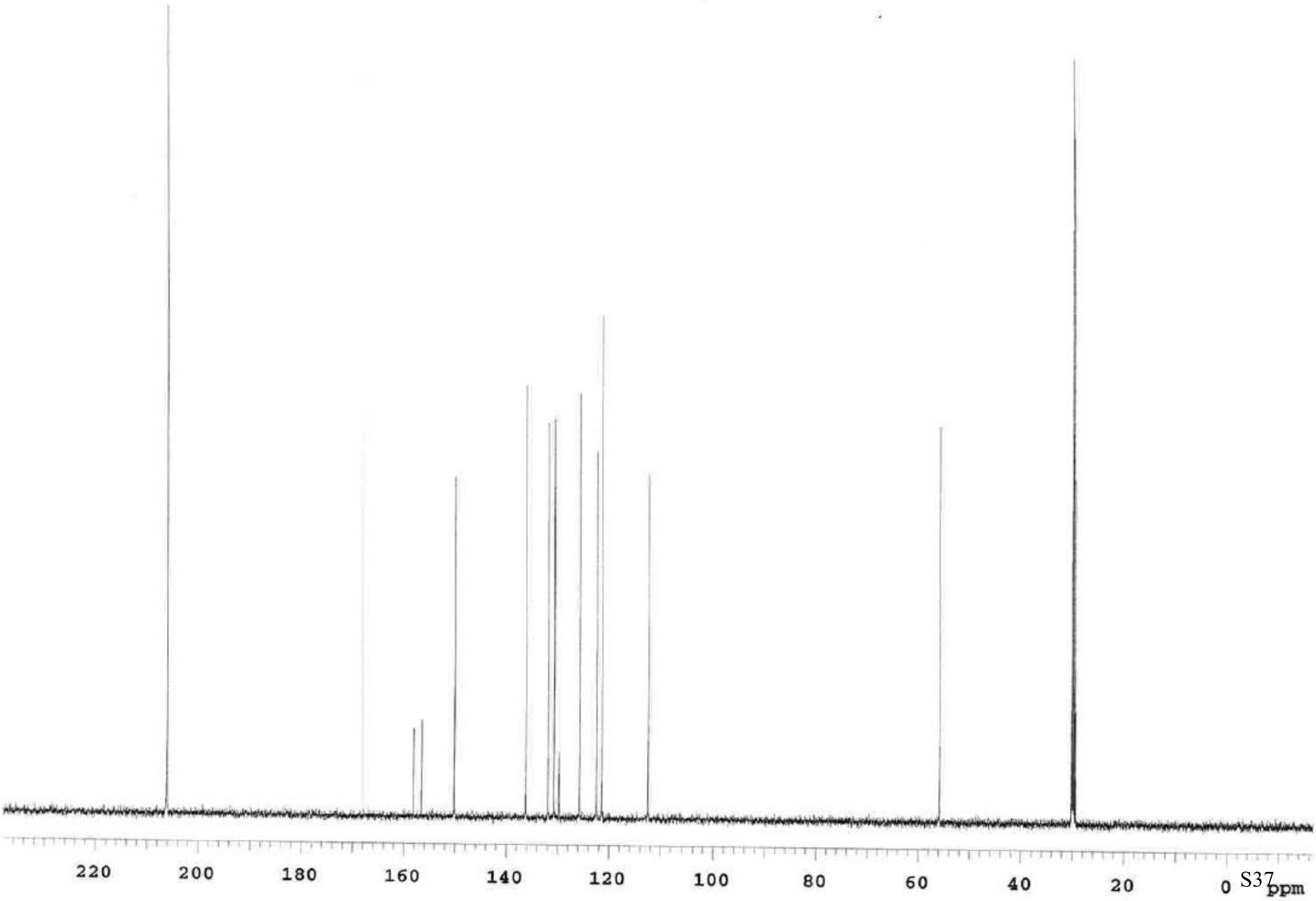

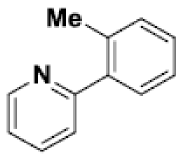

4f

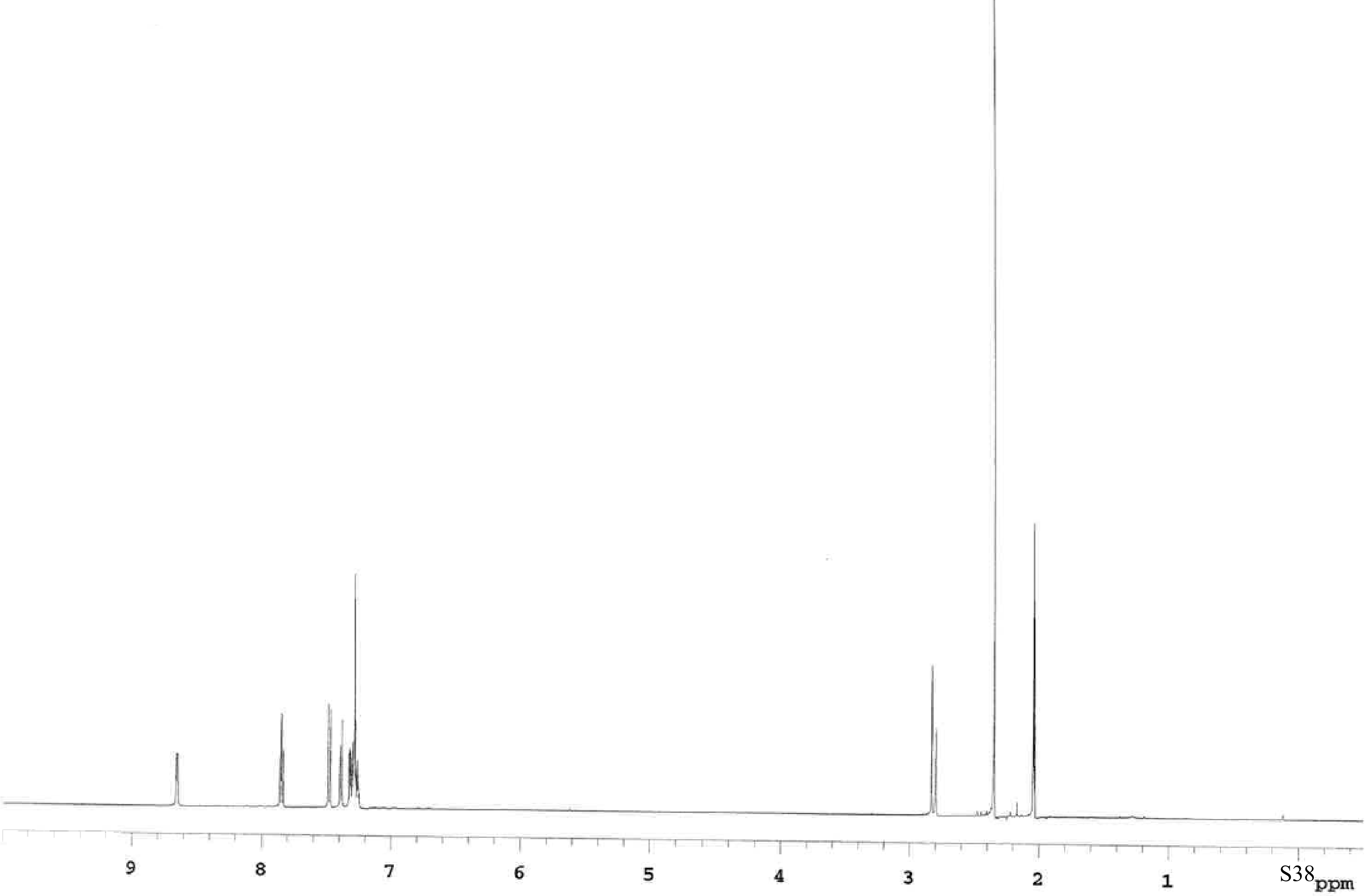

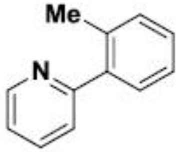

4f

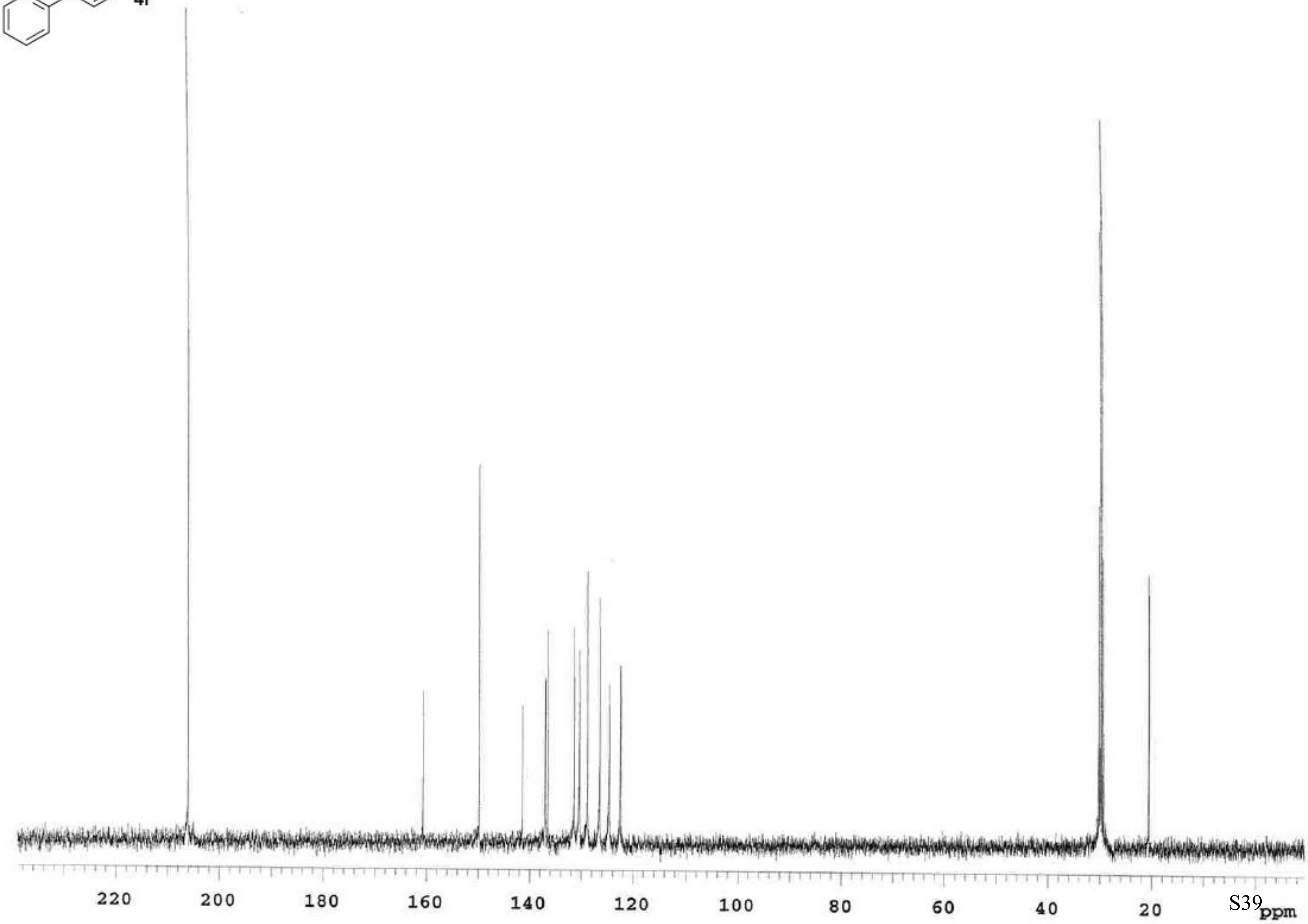

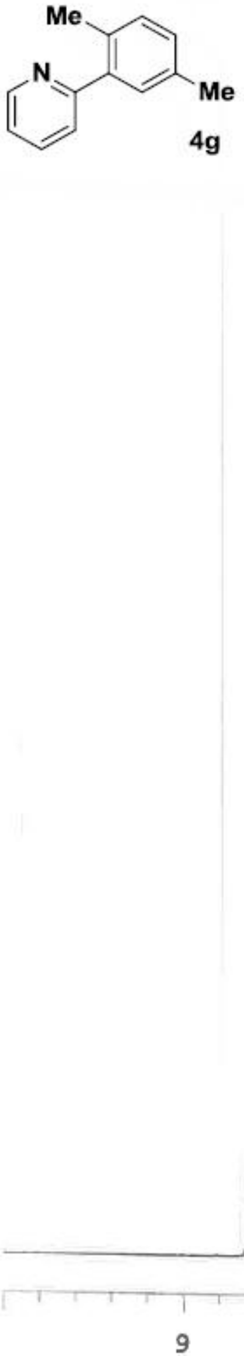

4g

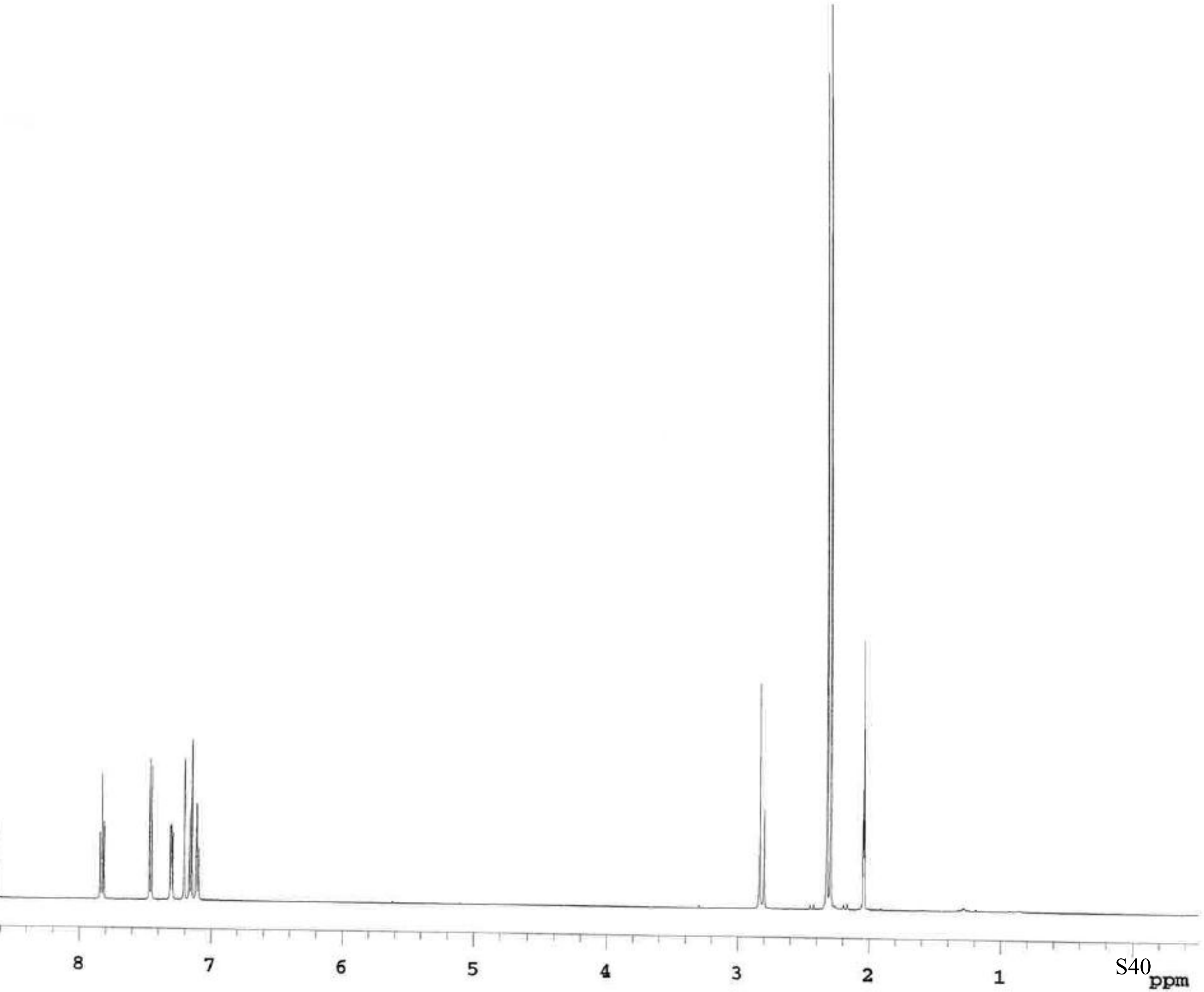

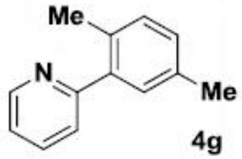

4g

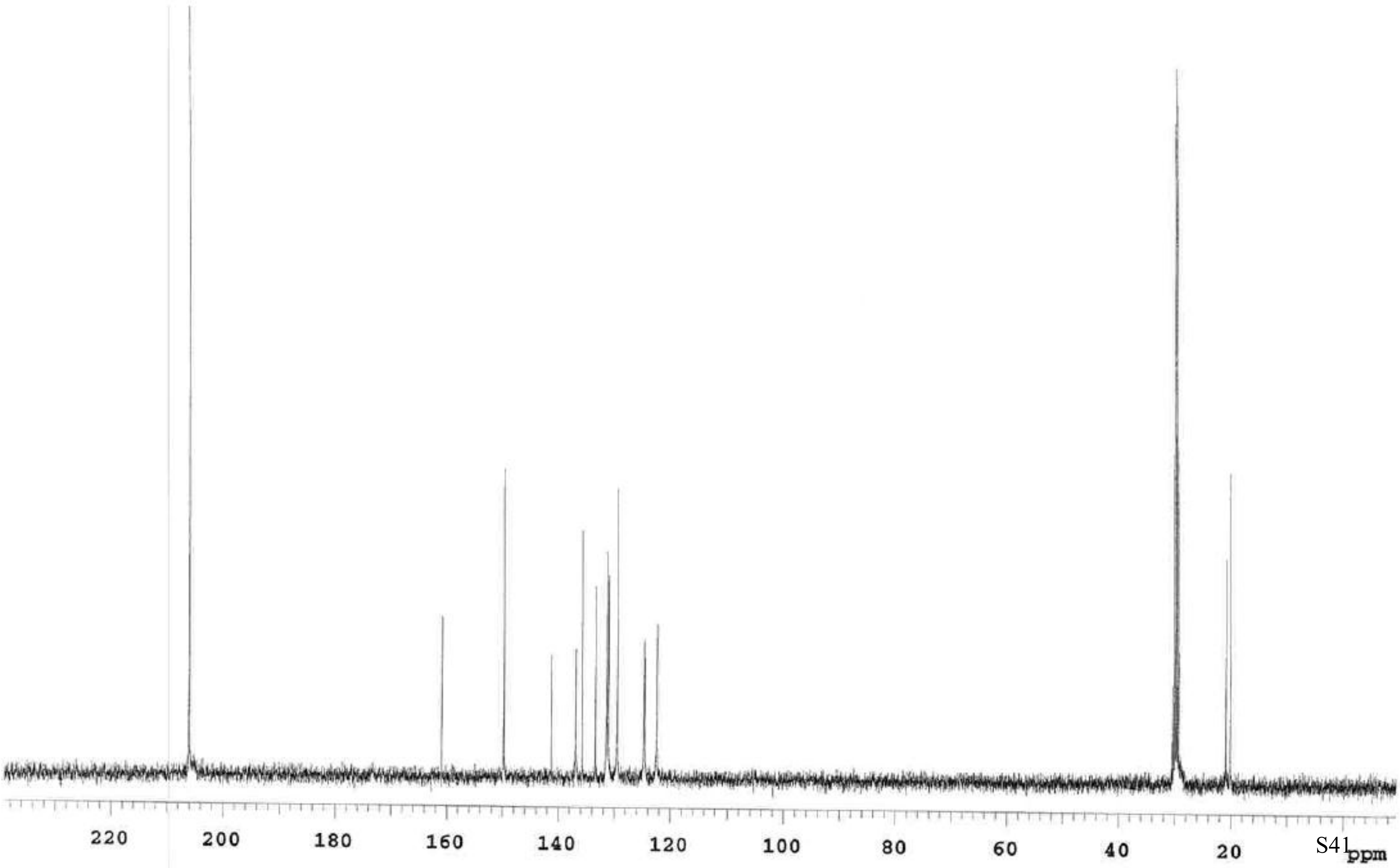

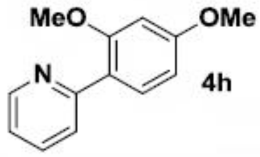

4h

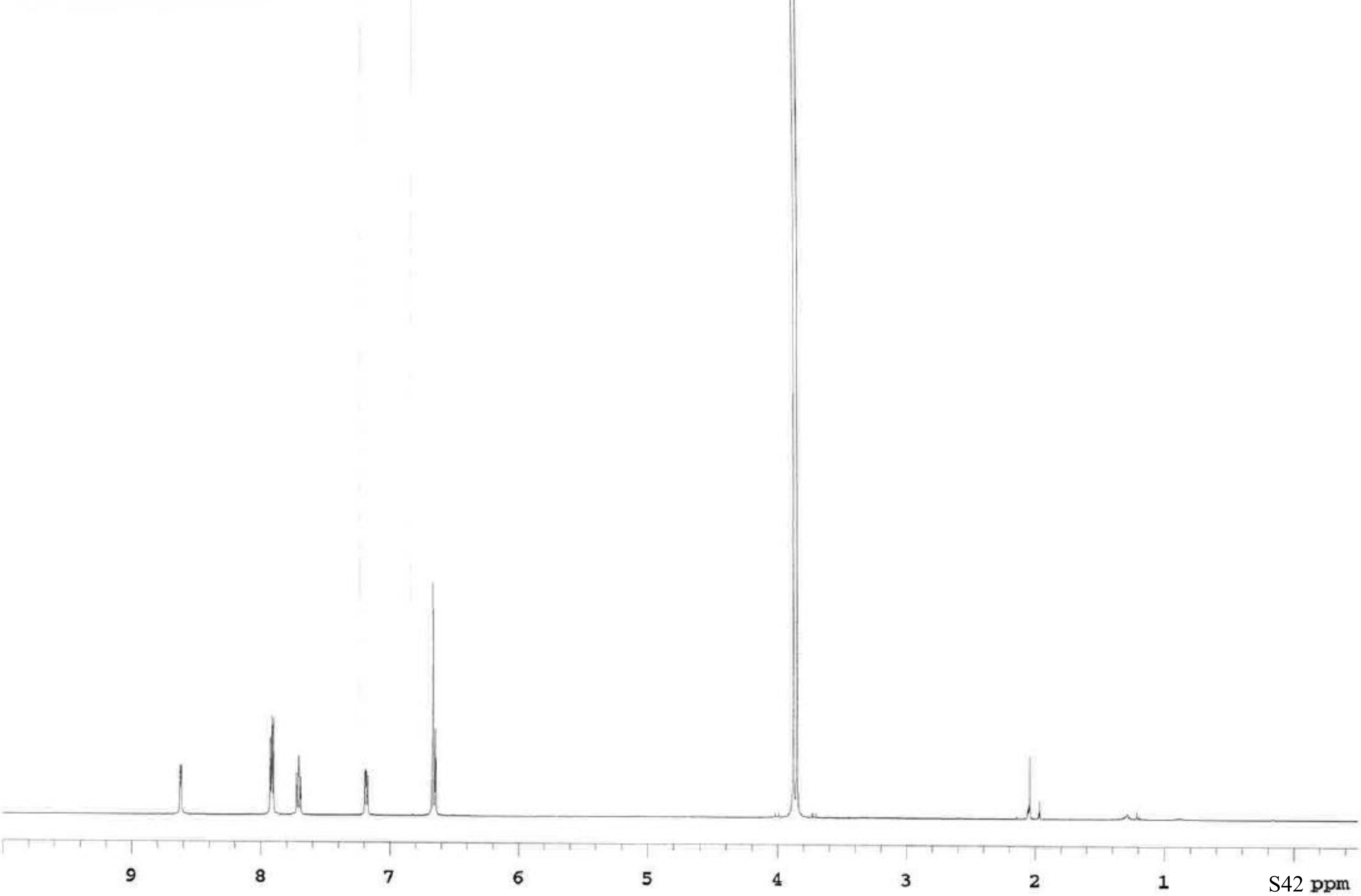

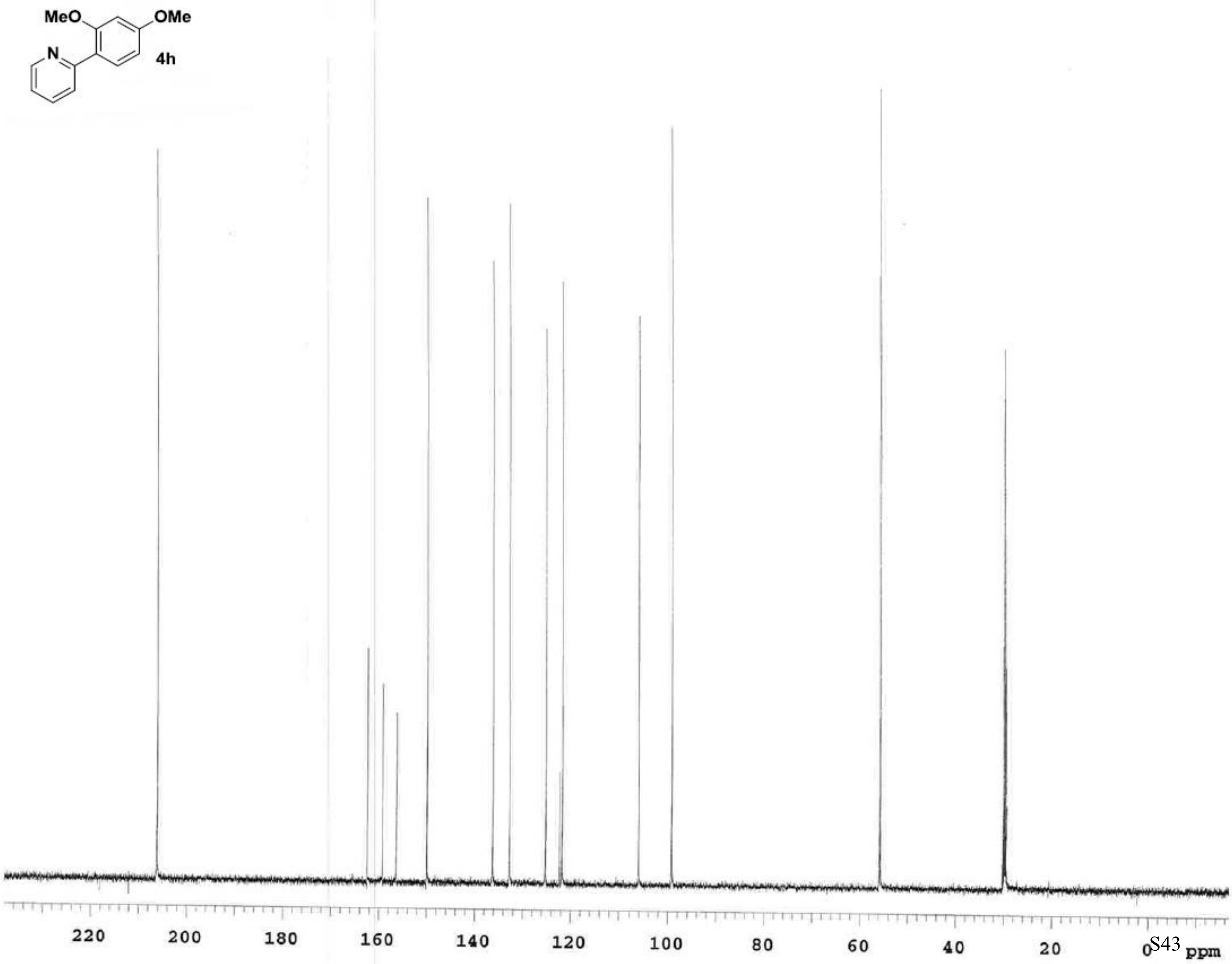
MeO
OMe
N
4h
220
200
180
160
140
120
100
80
60
40
20
0
ppm

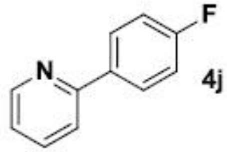

4j

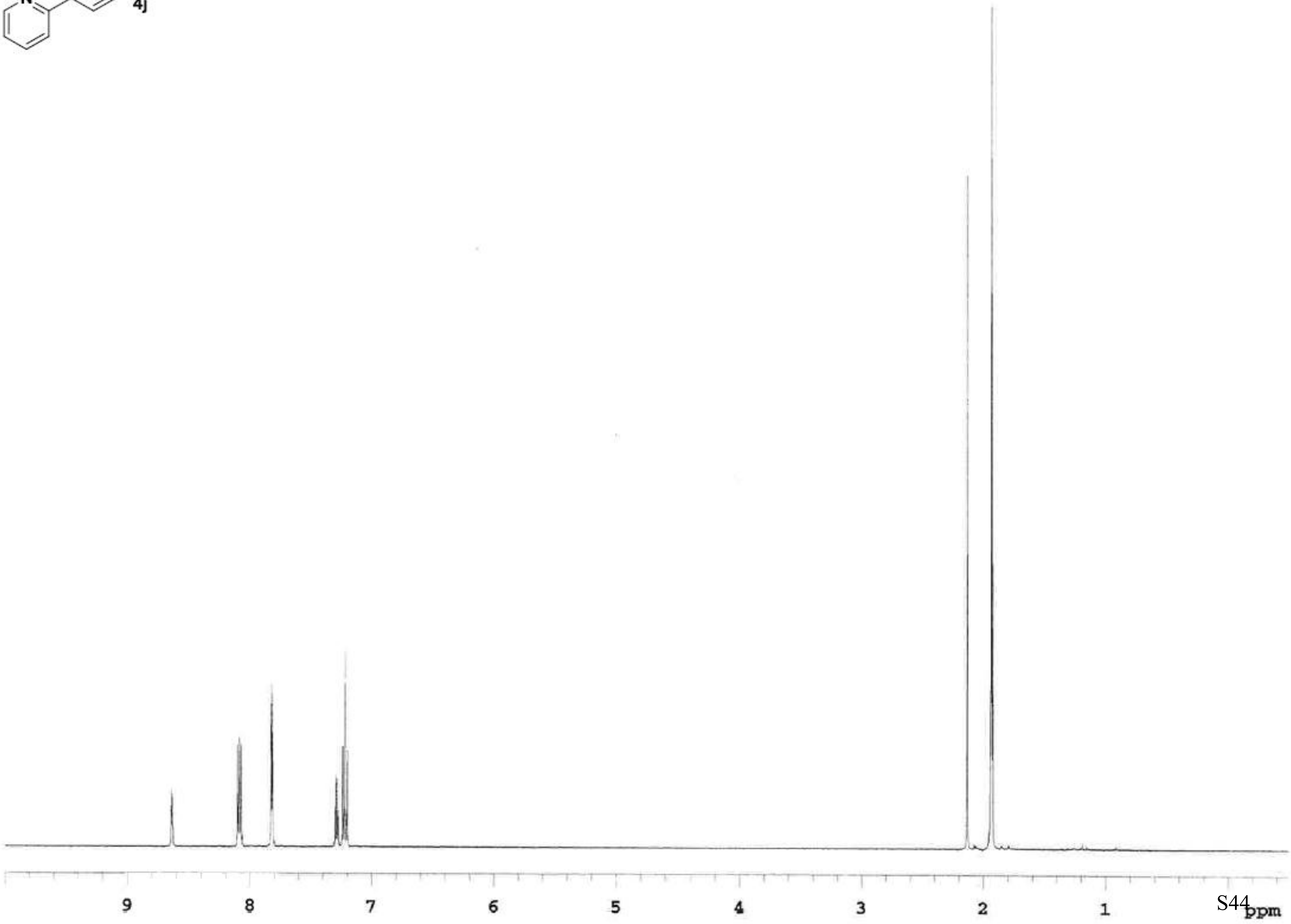

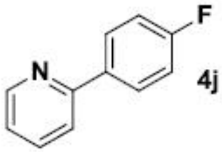

4j

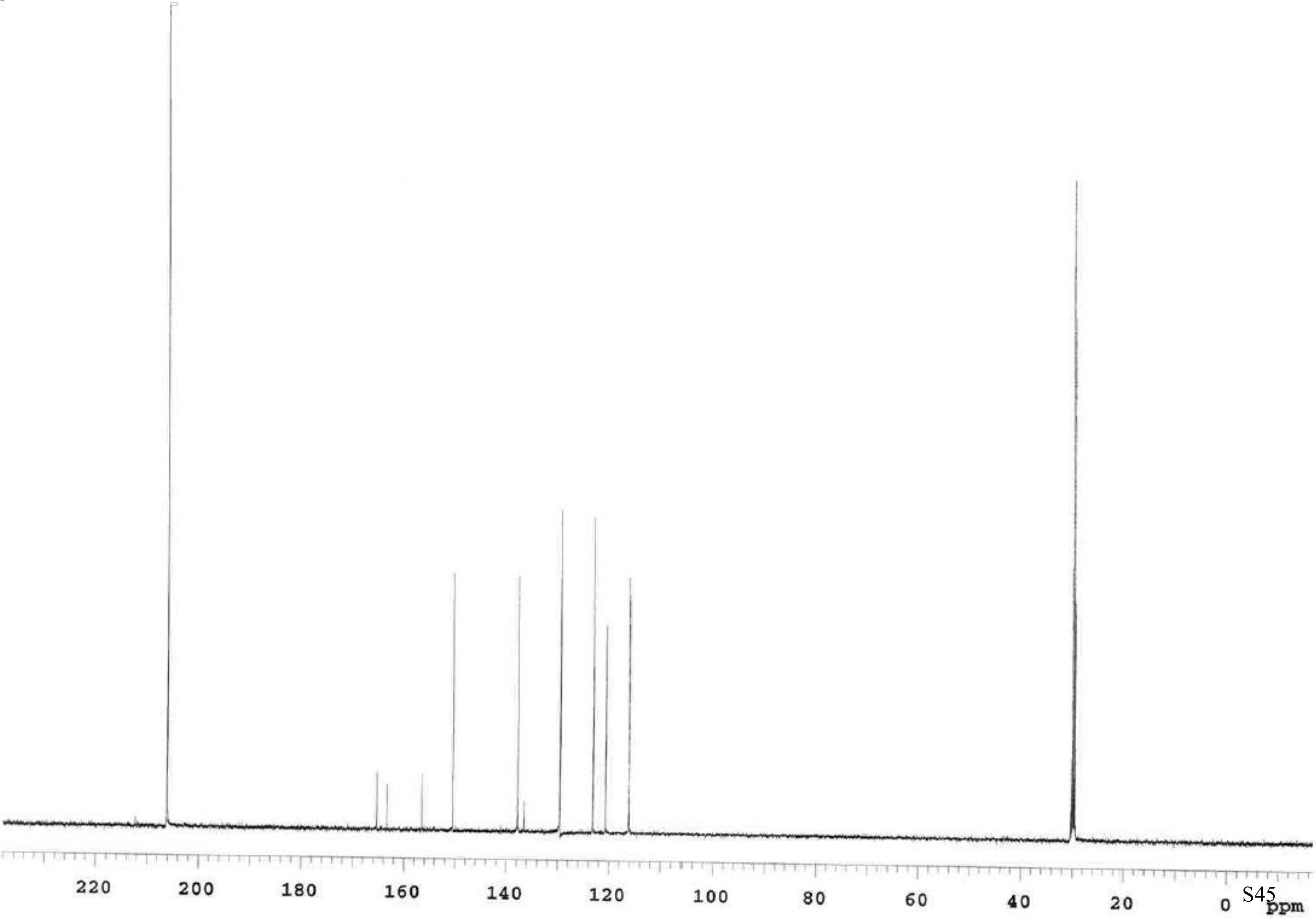

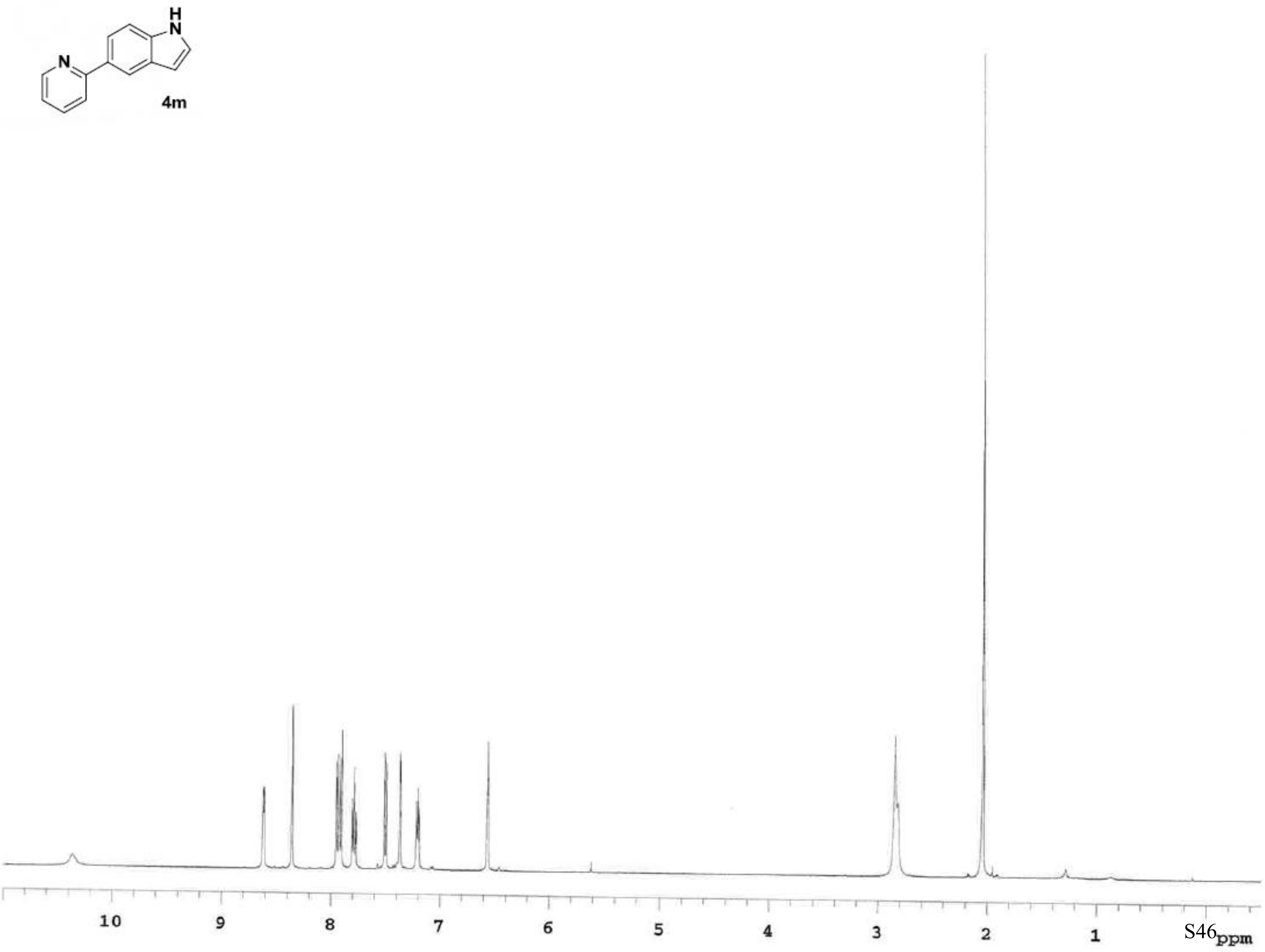
H
N
N
4m
10
9
8
7
6
5
4
3
2
1
ppm

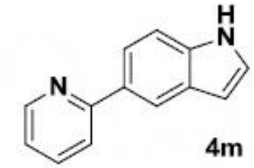

4m

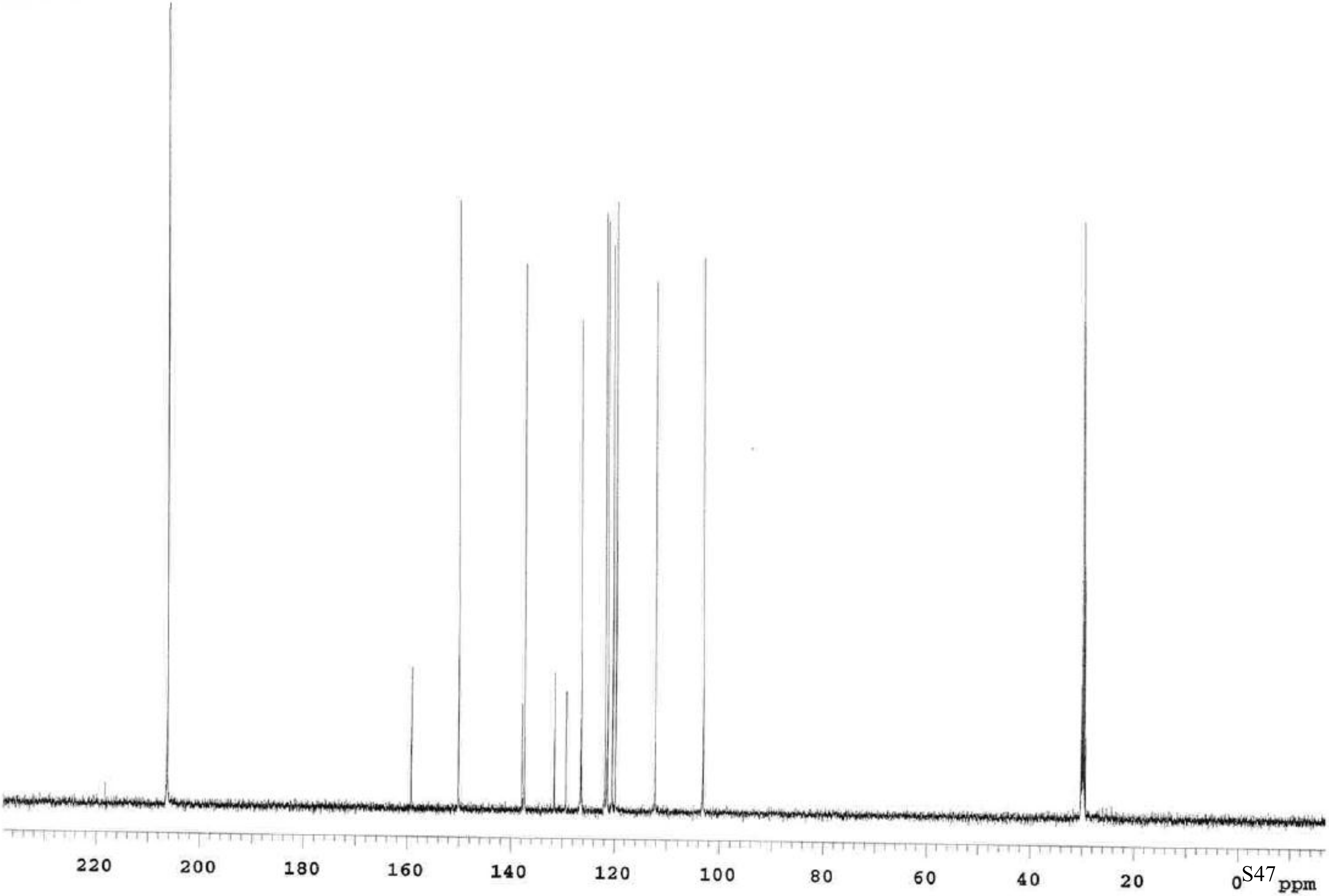

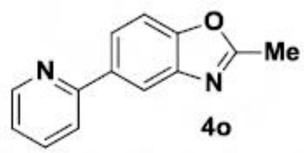

4o

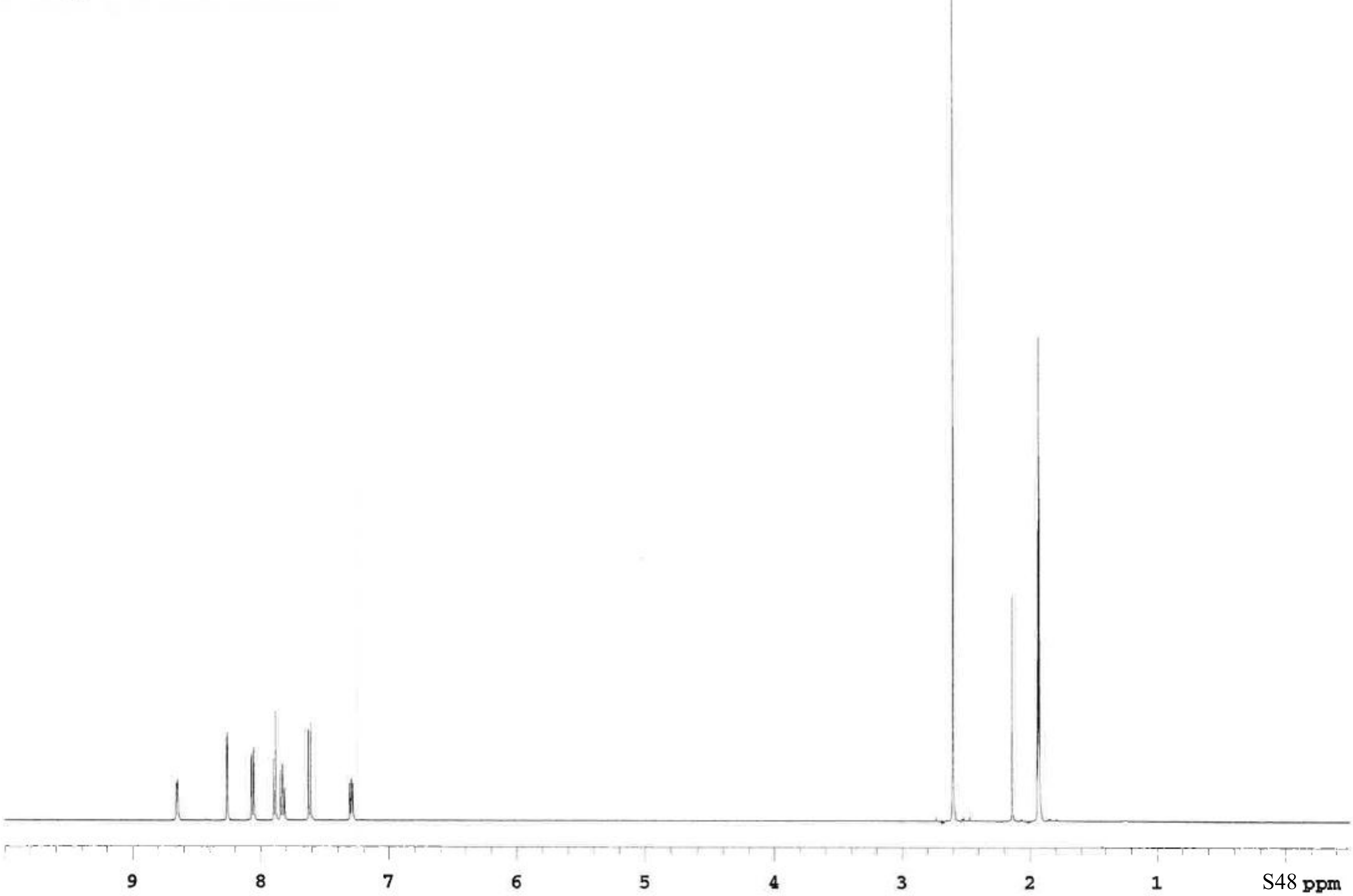

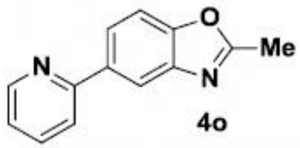
4o

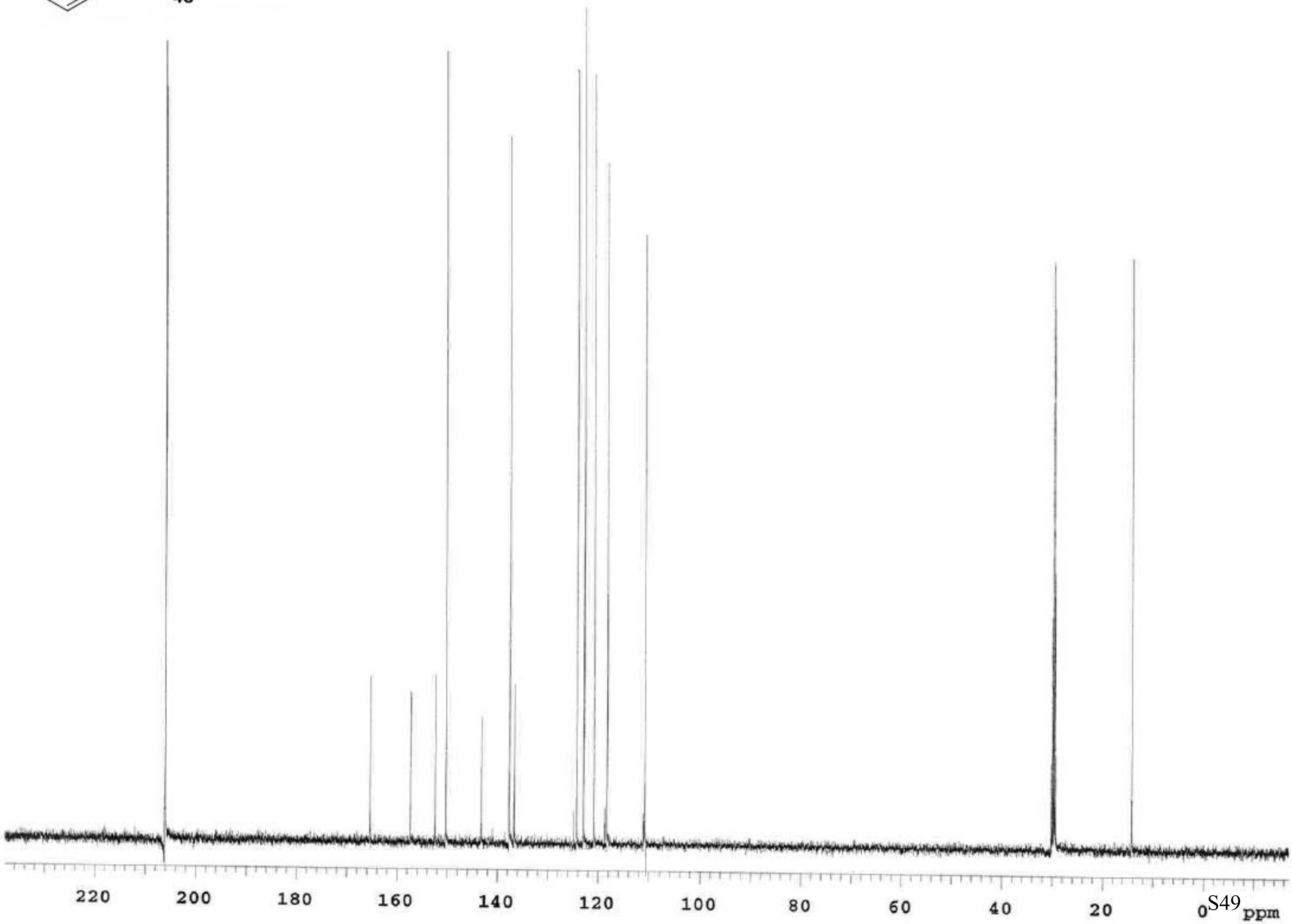

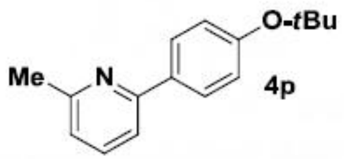

4p

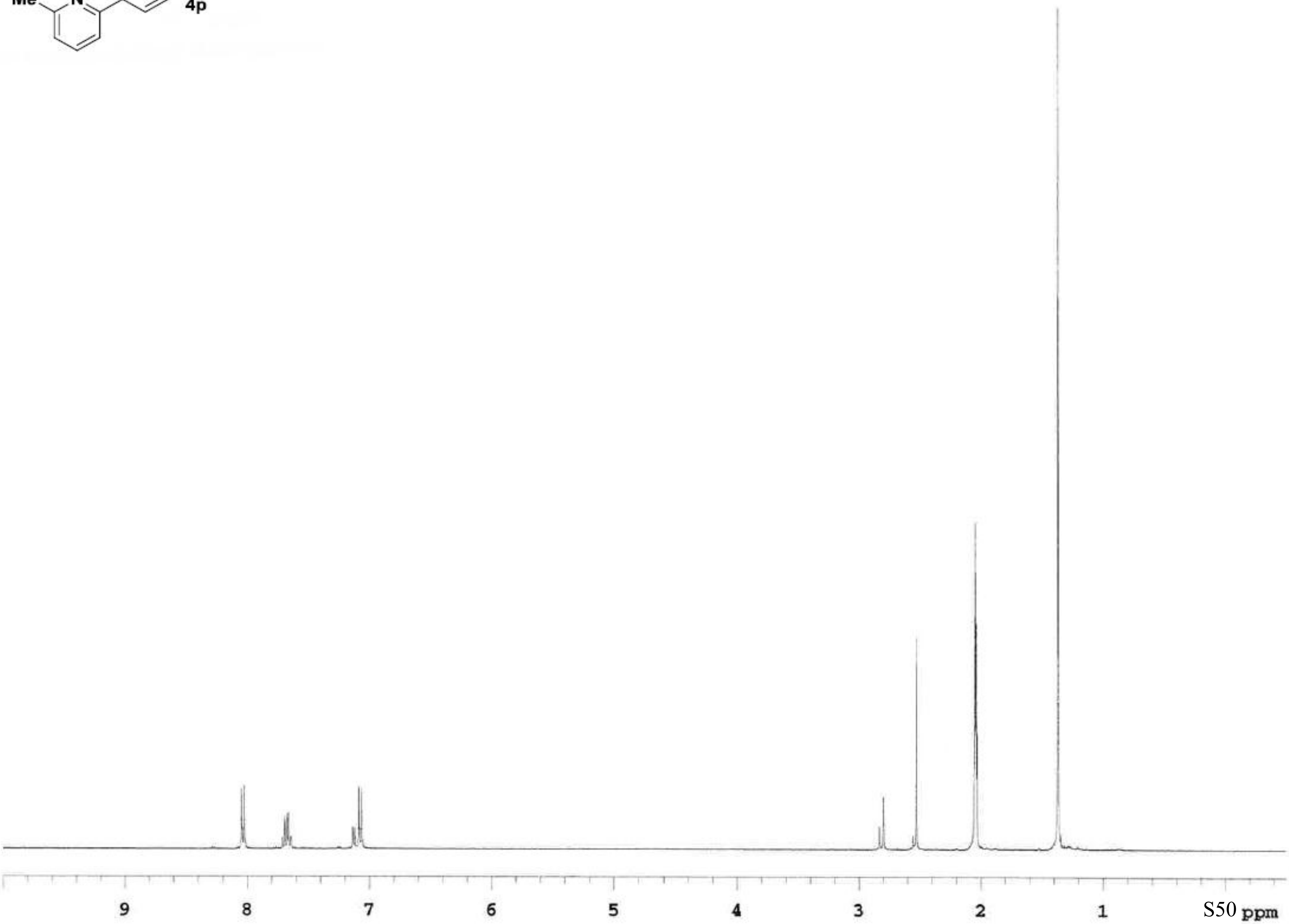

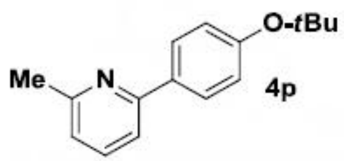

4p

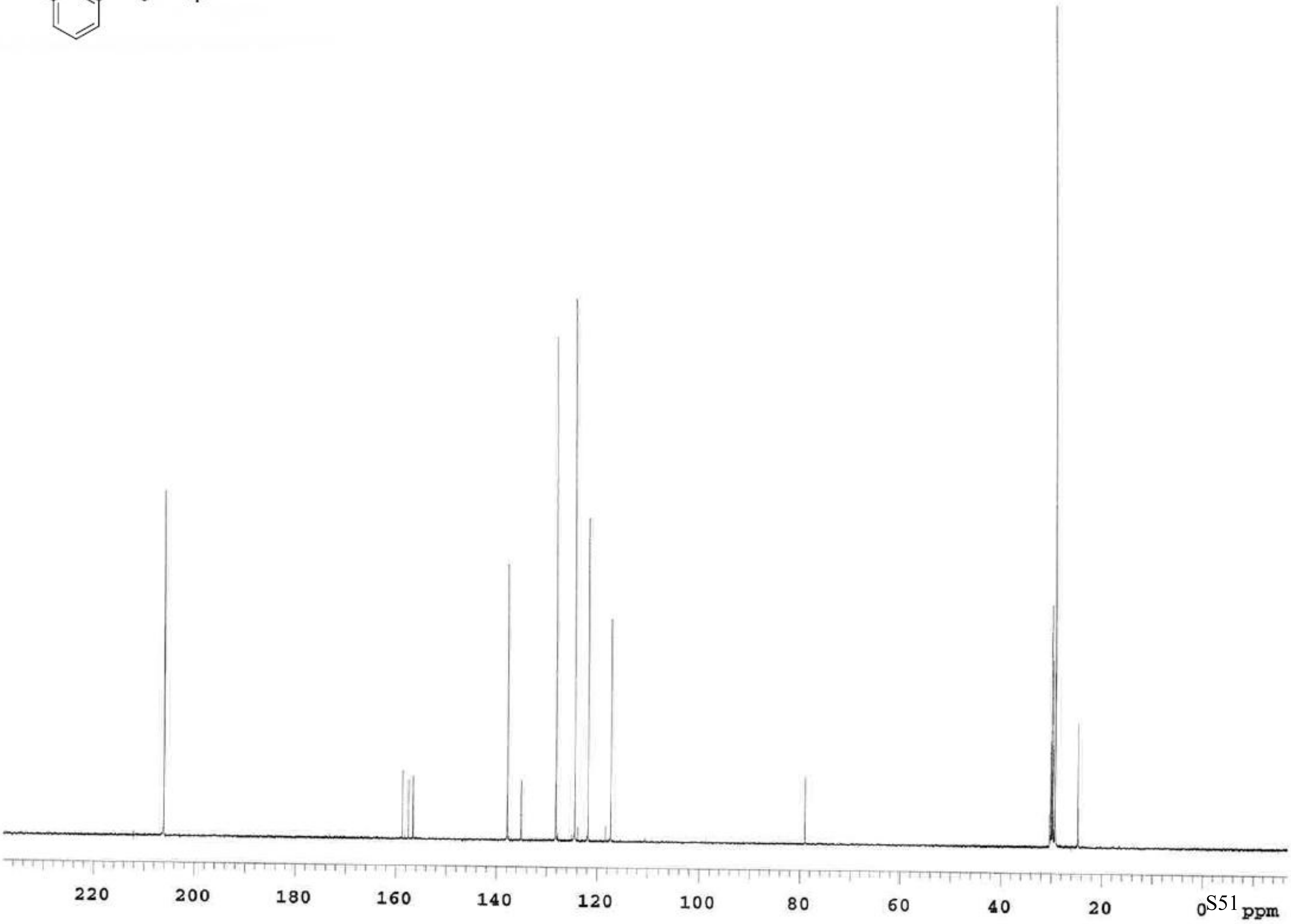

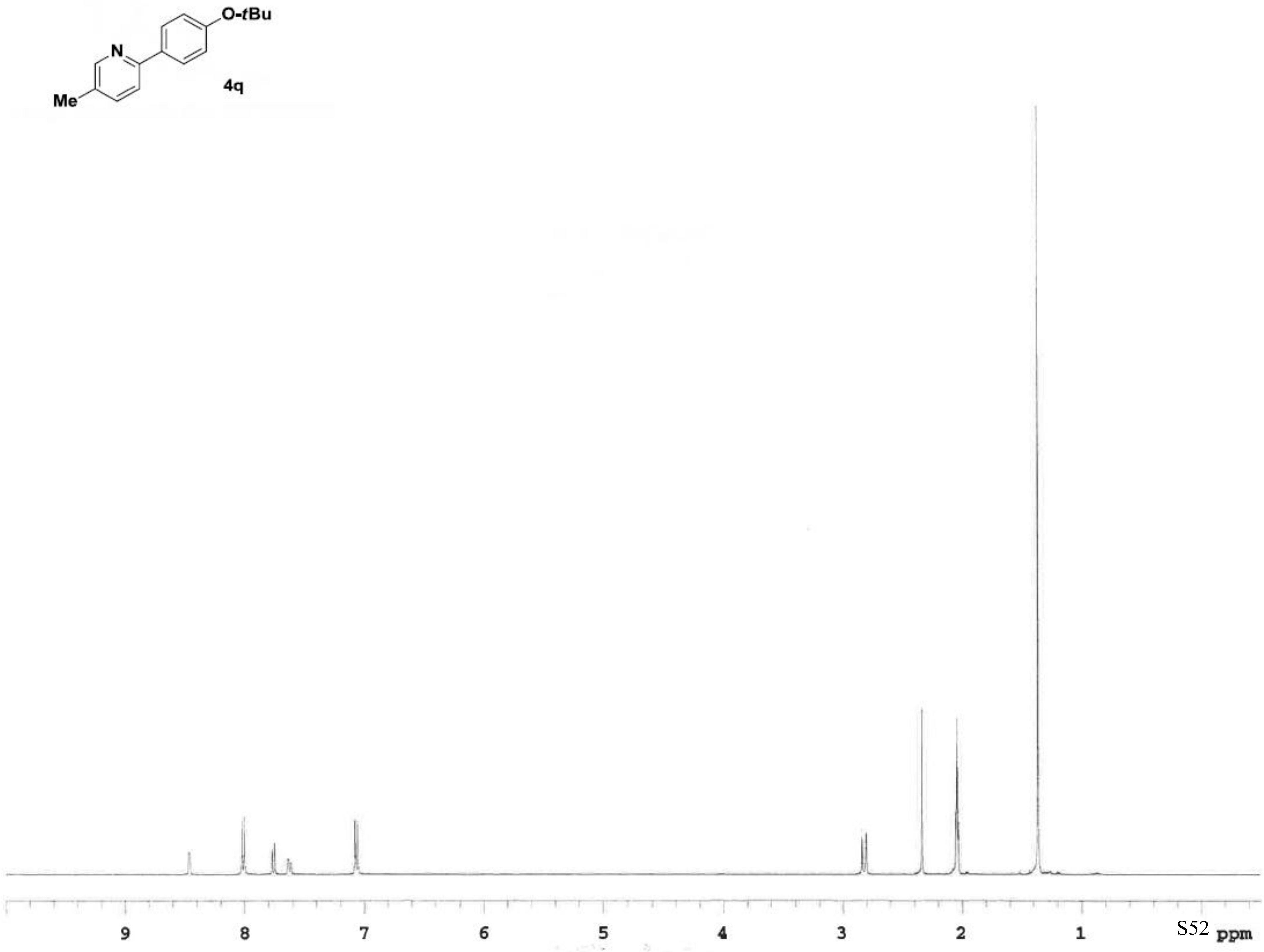
O-tBu
N
Me
4q
9
8
7
6
5
4
3
2
1
ppm

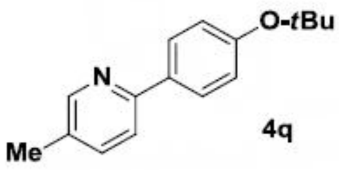

4q

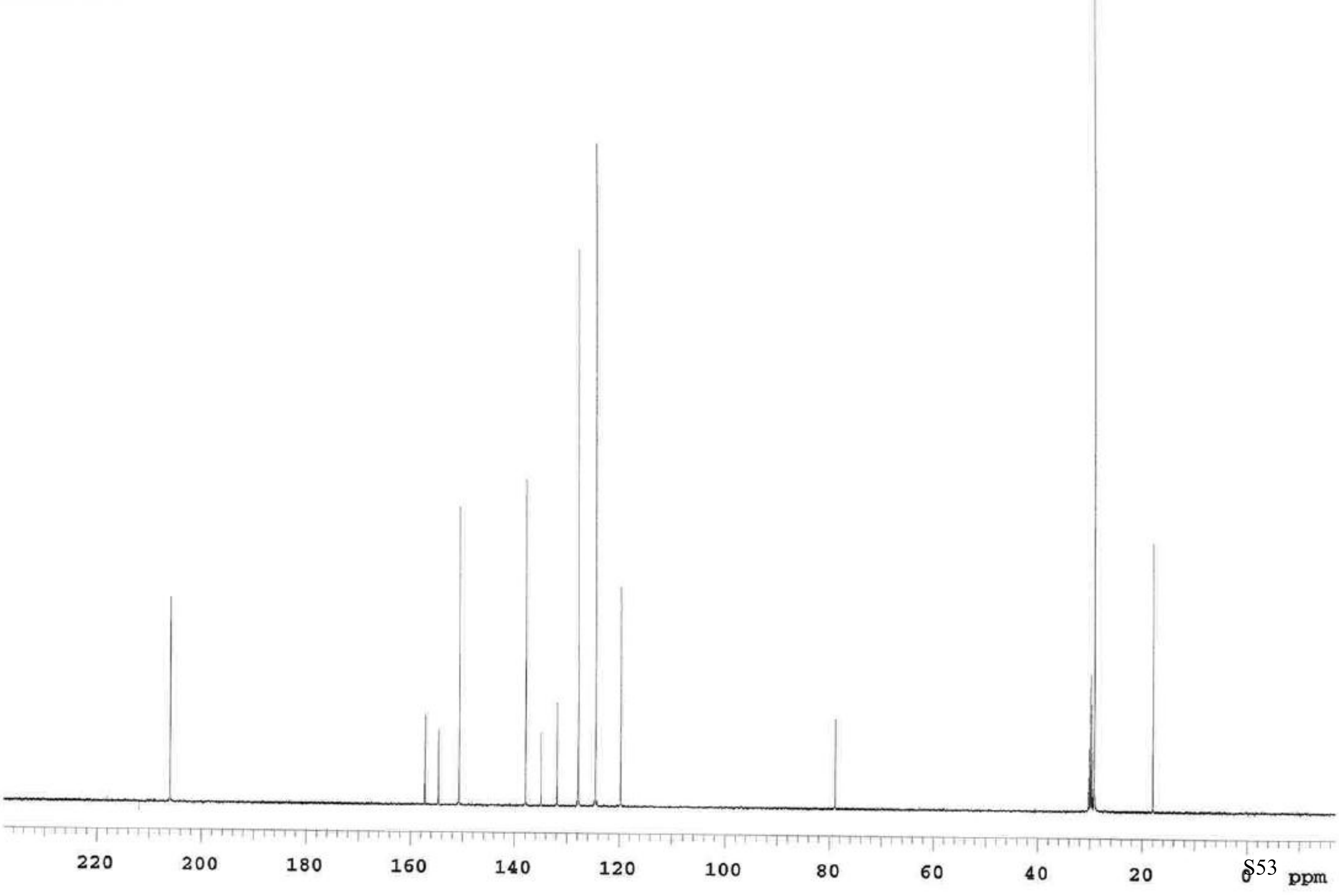

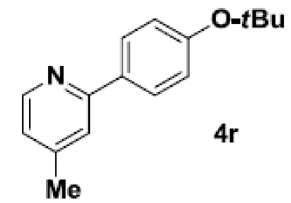

4r

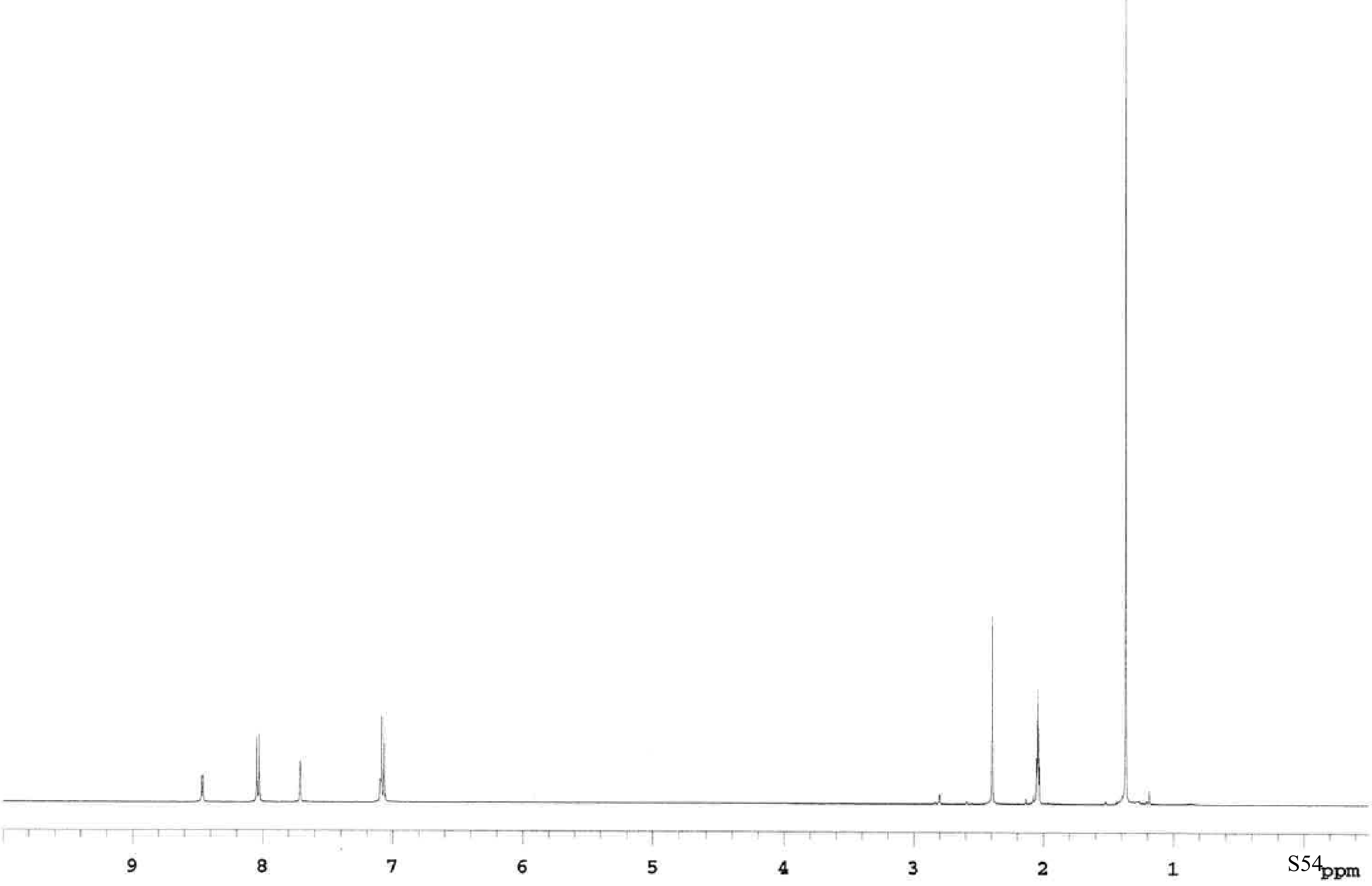

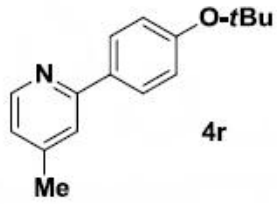

4r

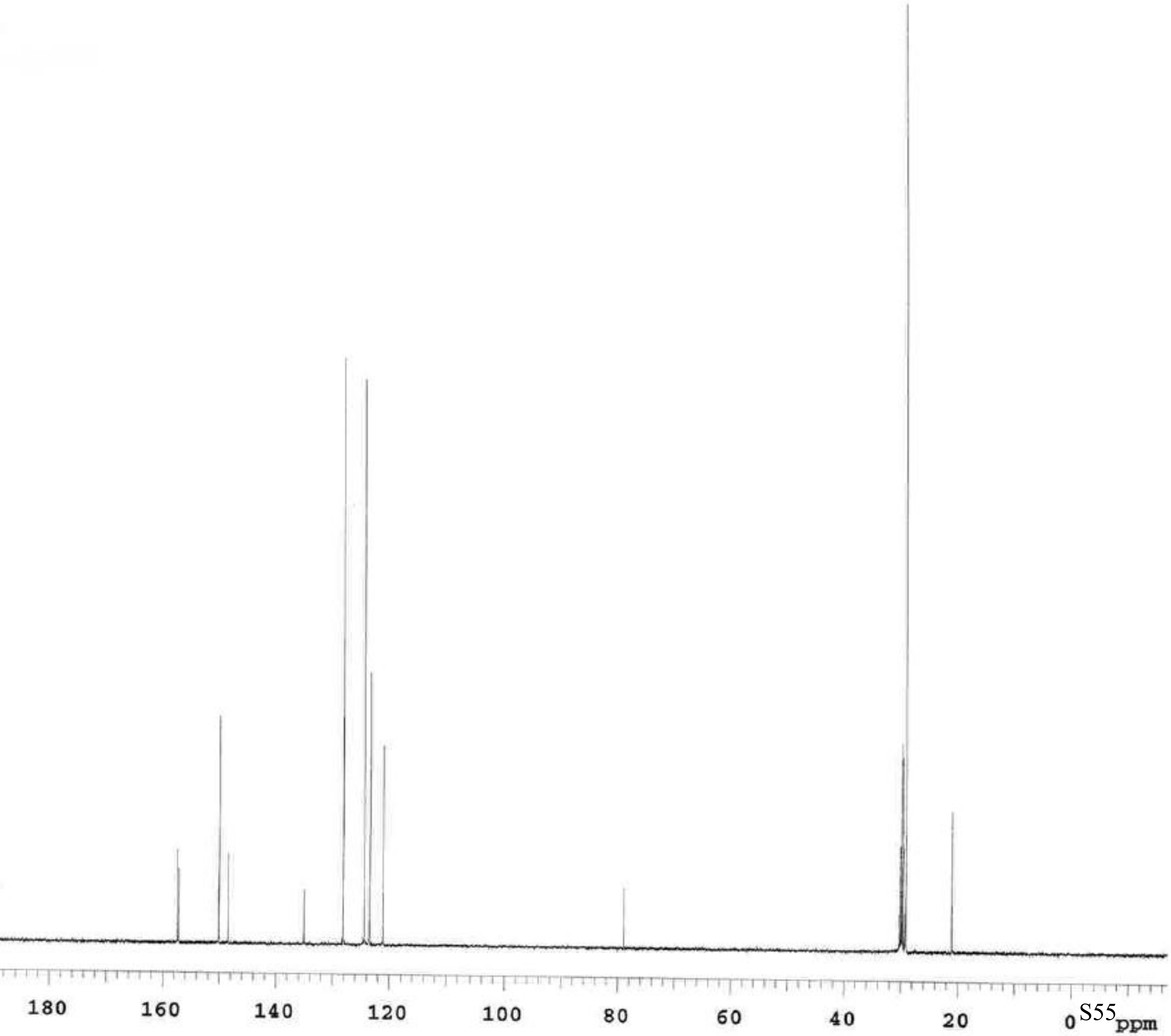

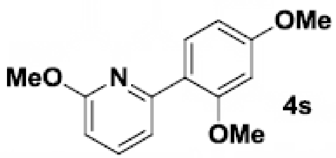

4s

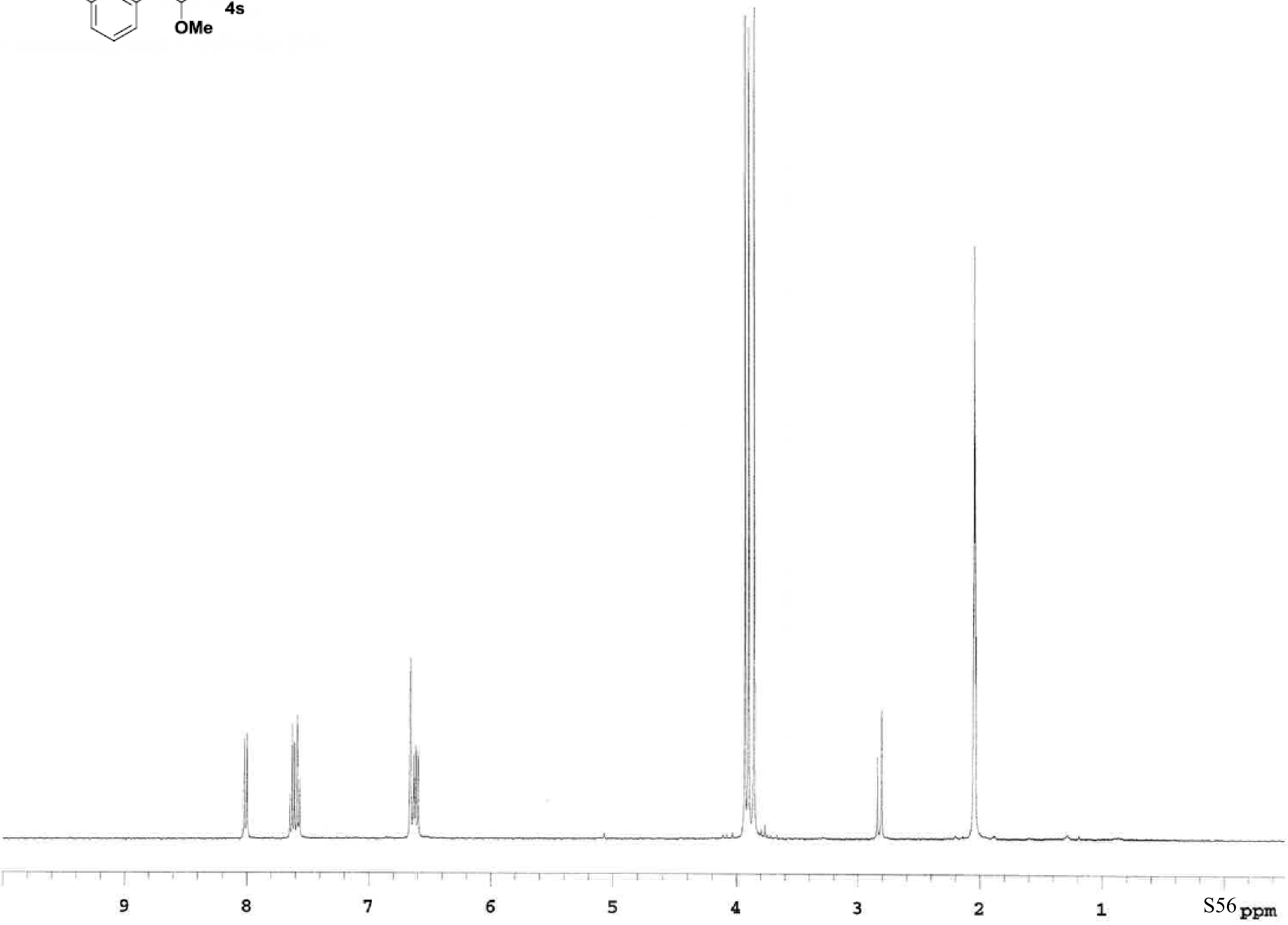

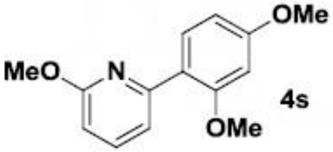

4s

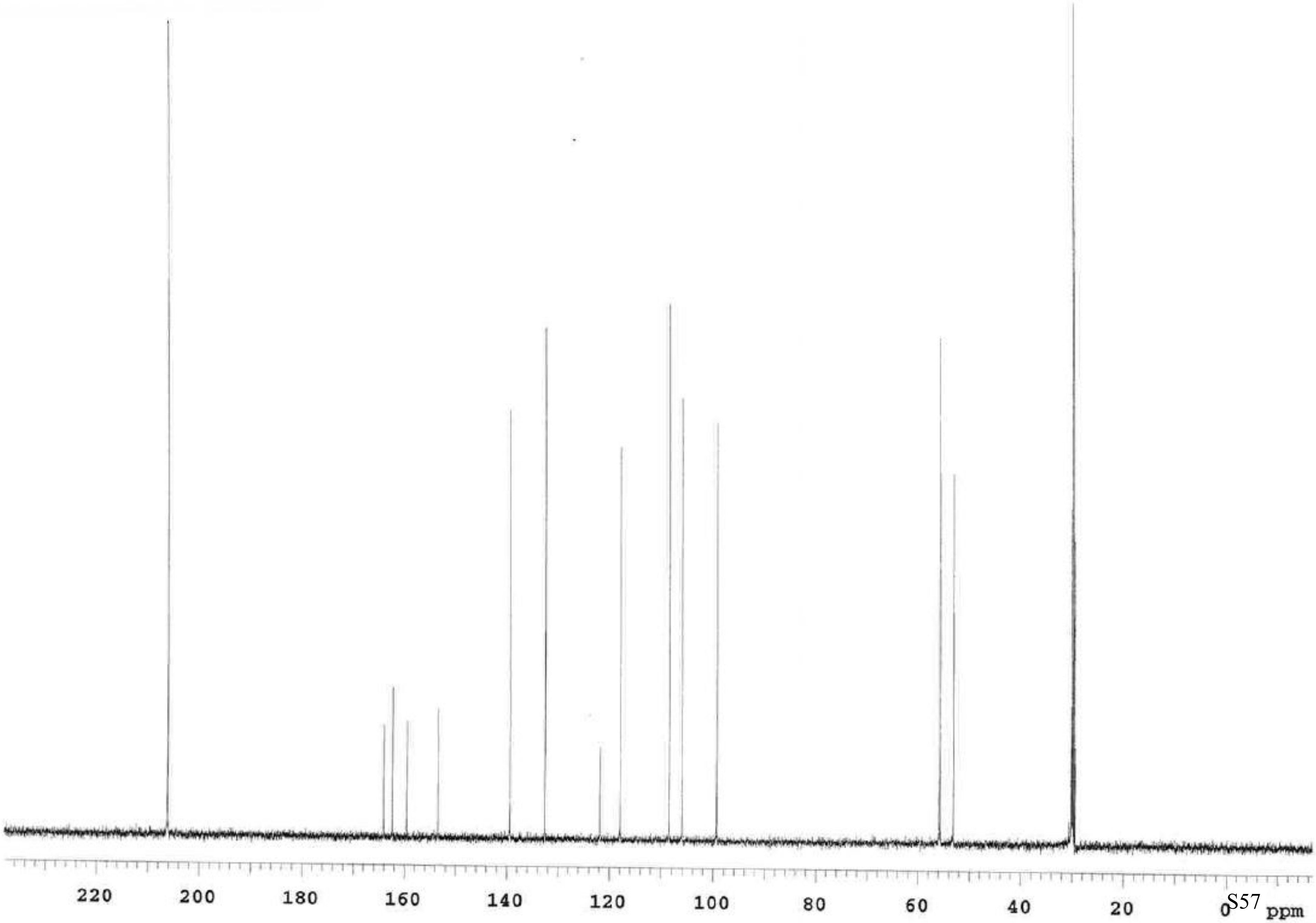

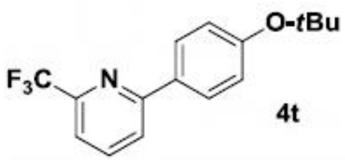

4t

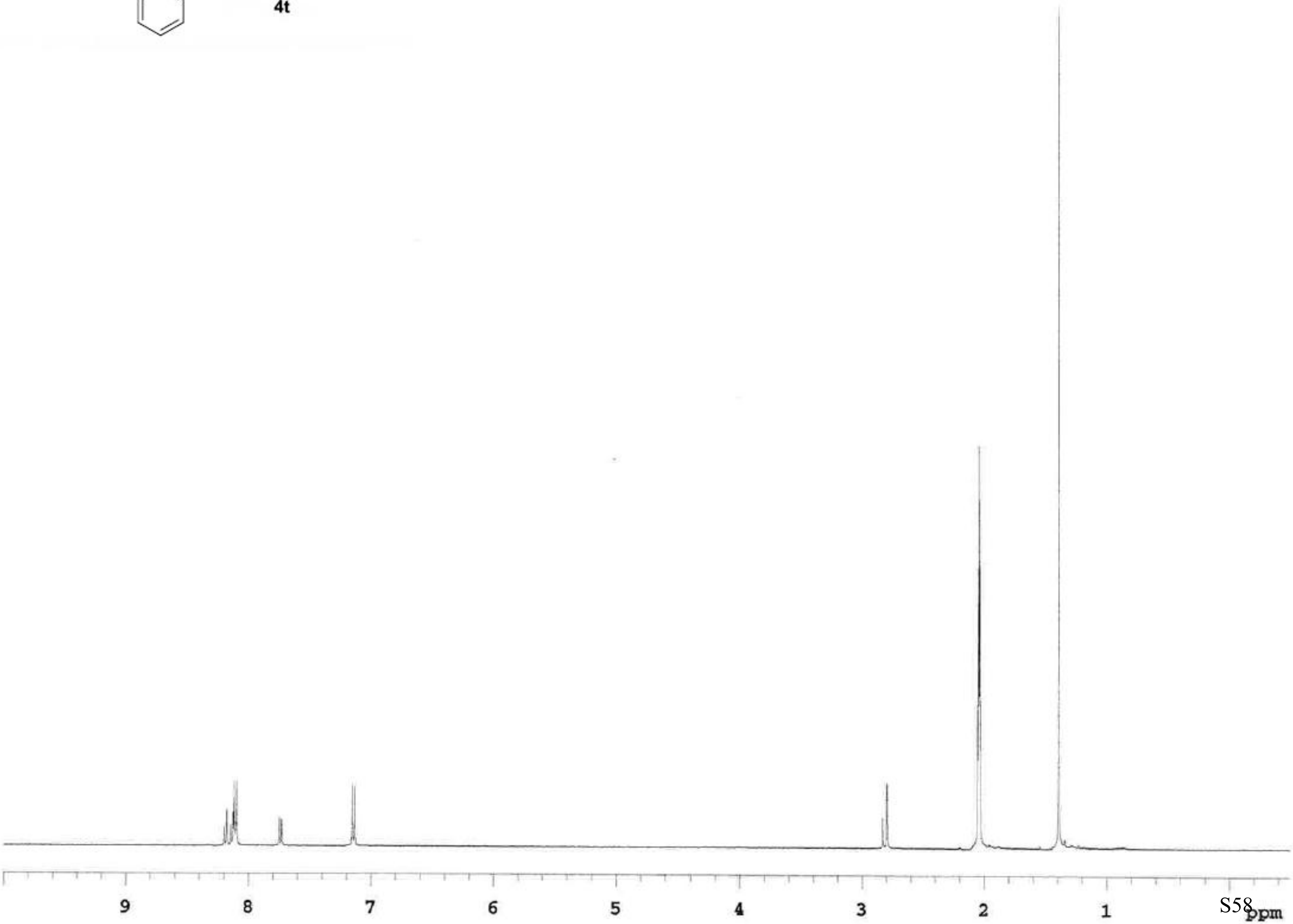

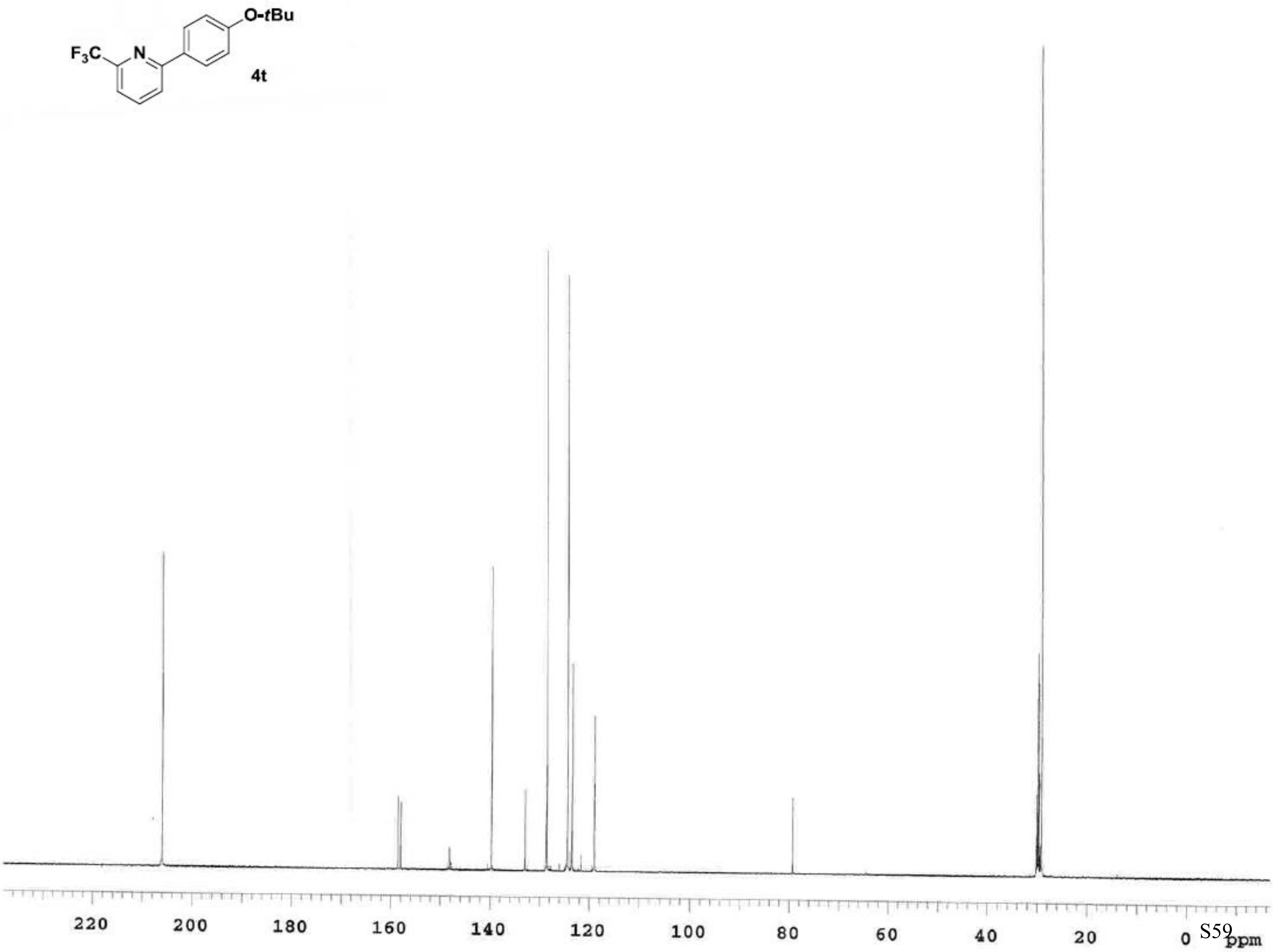
O-tBu
F3C
N
4t
220
200
180
160
140
120
100
80
60
40
20
0
ppm

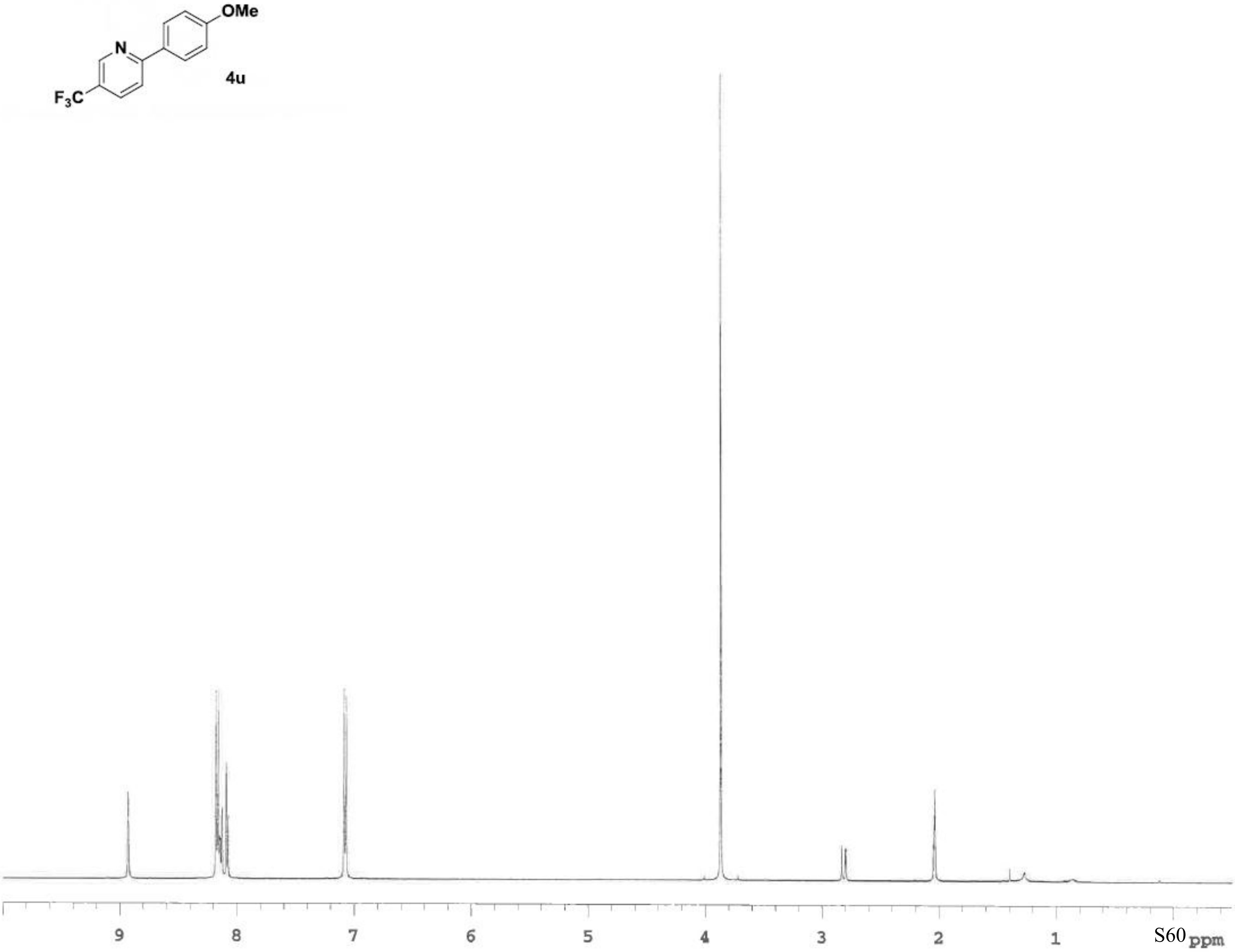
OMe
N
F_3C
4u
9
8
7
6
5
4
3
2
1
ppm

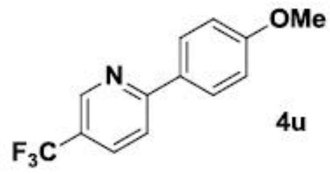

4u

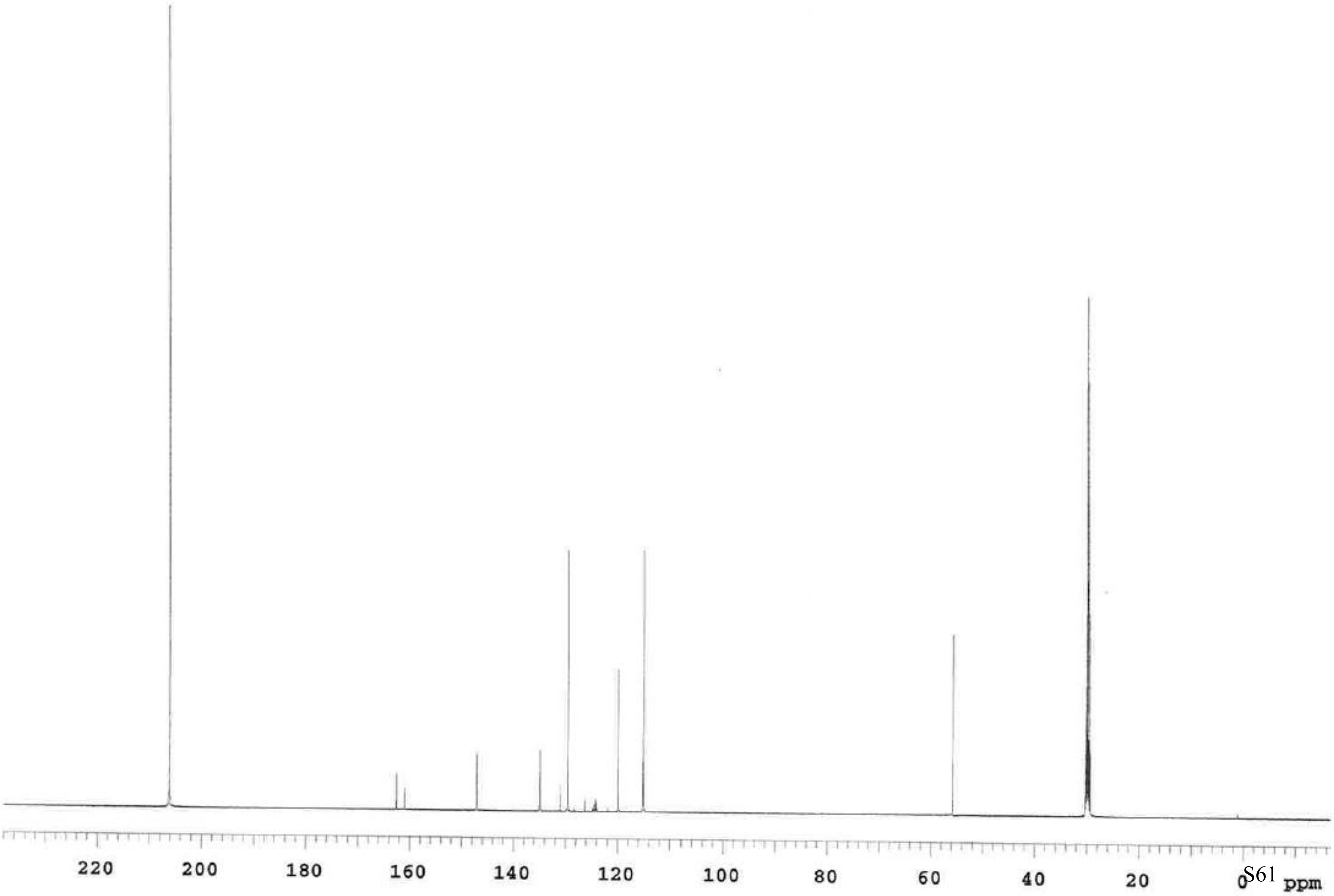

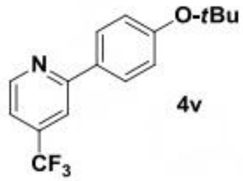

4v

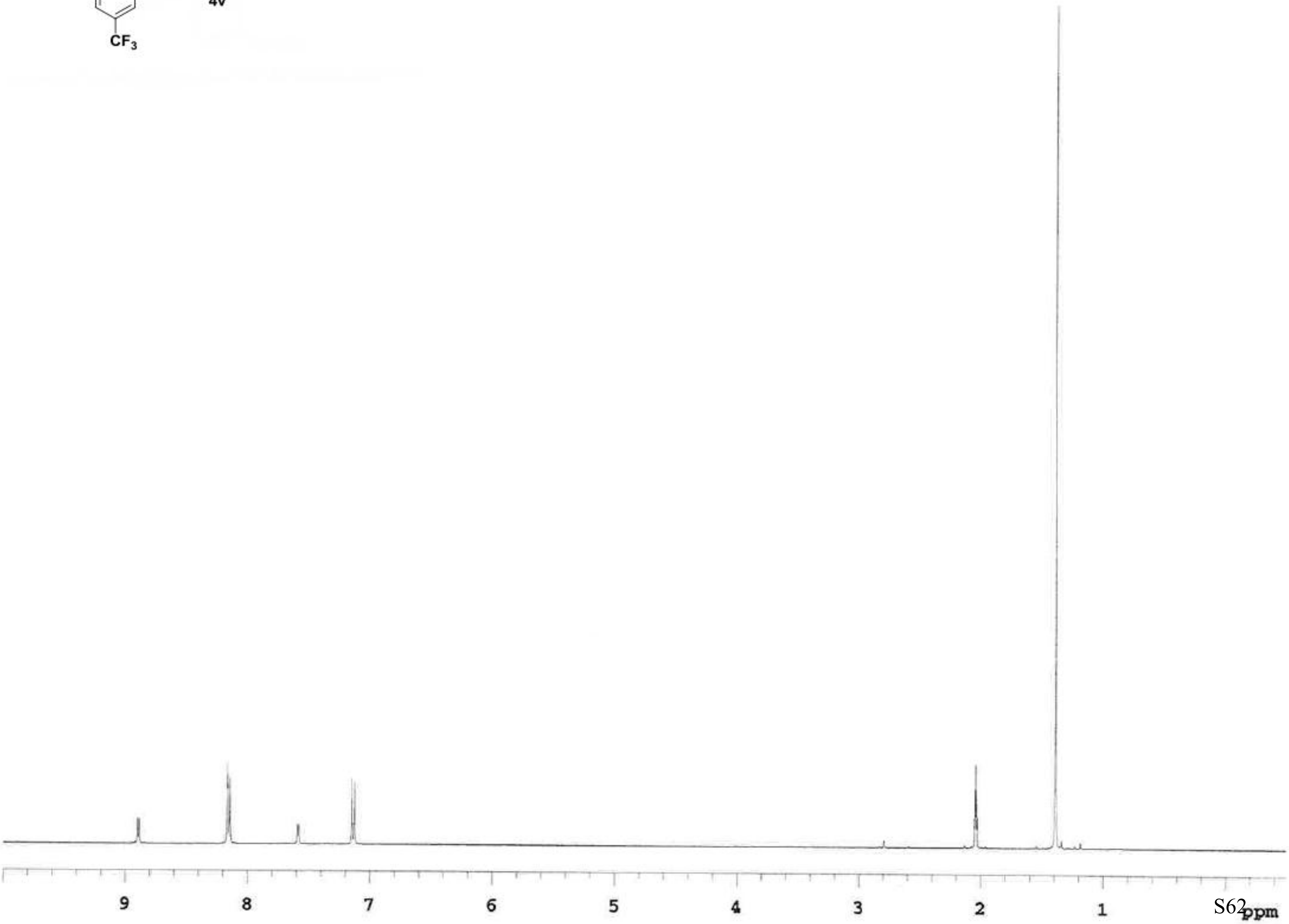

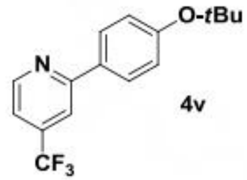

4v

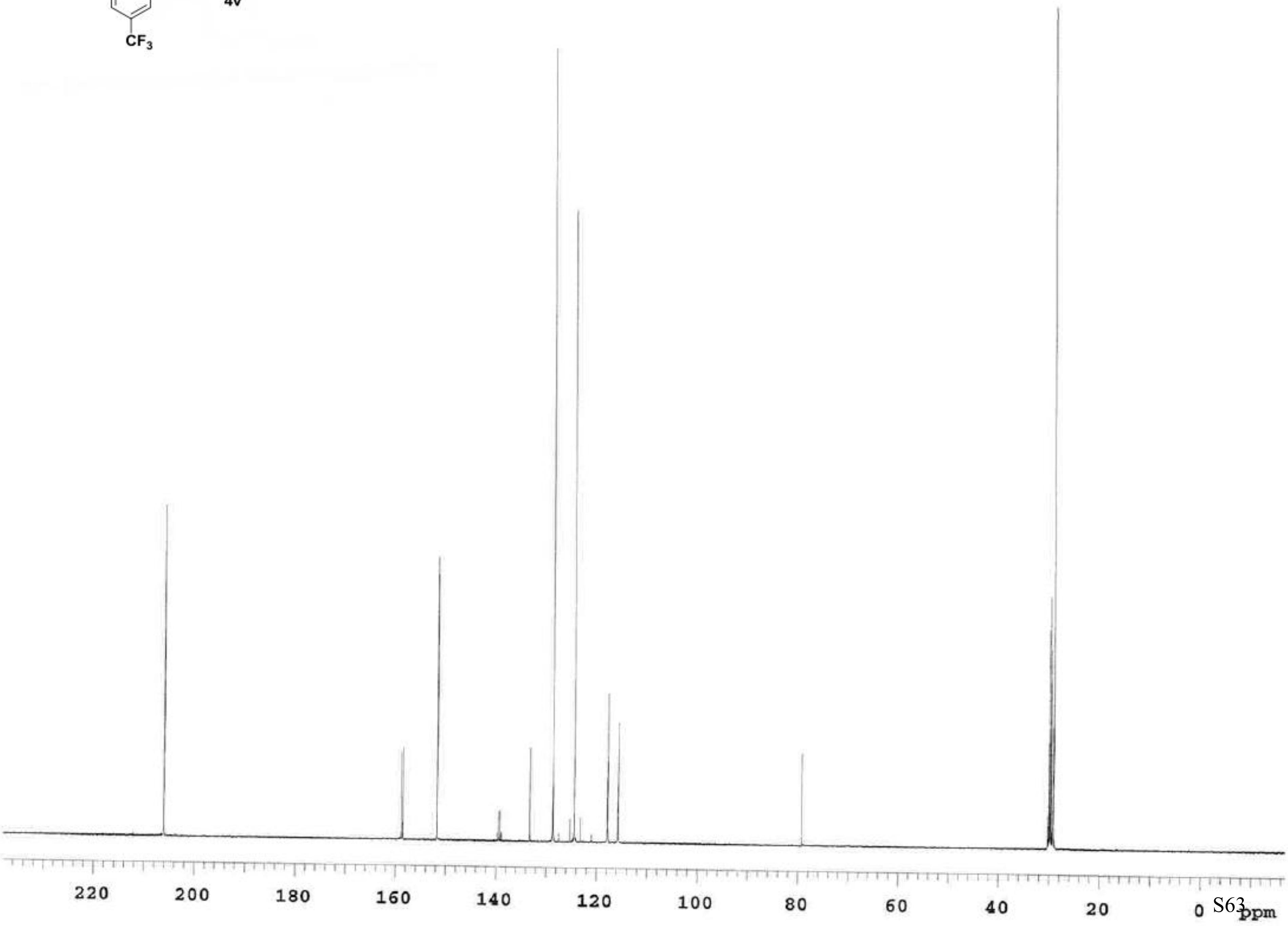

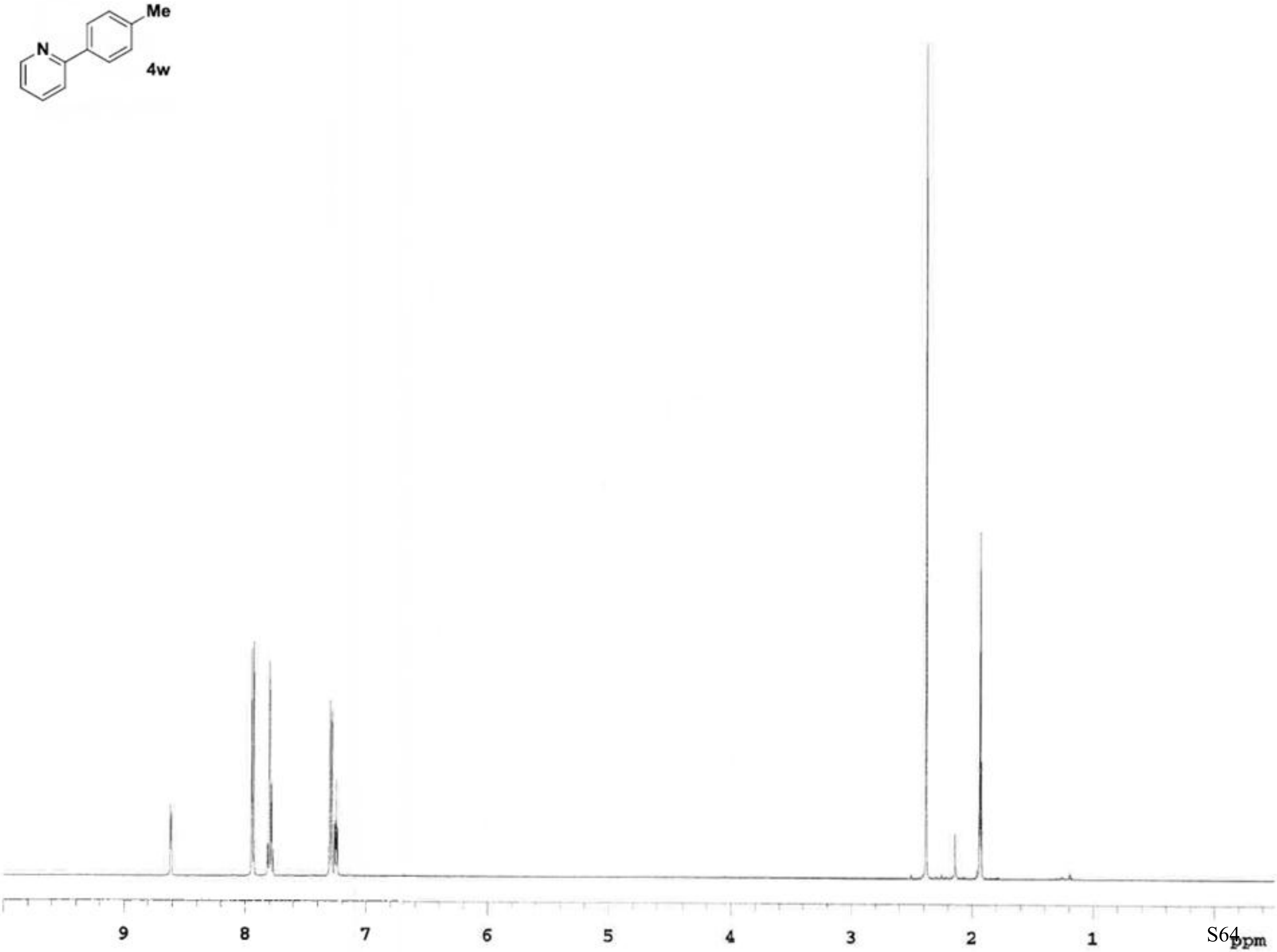

4w

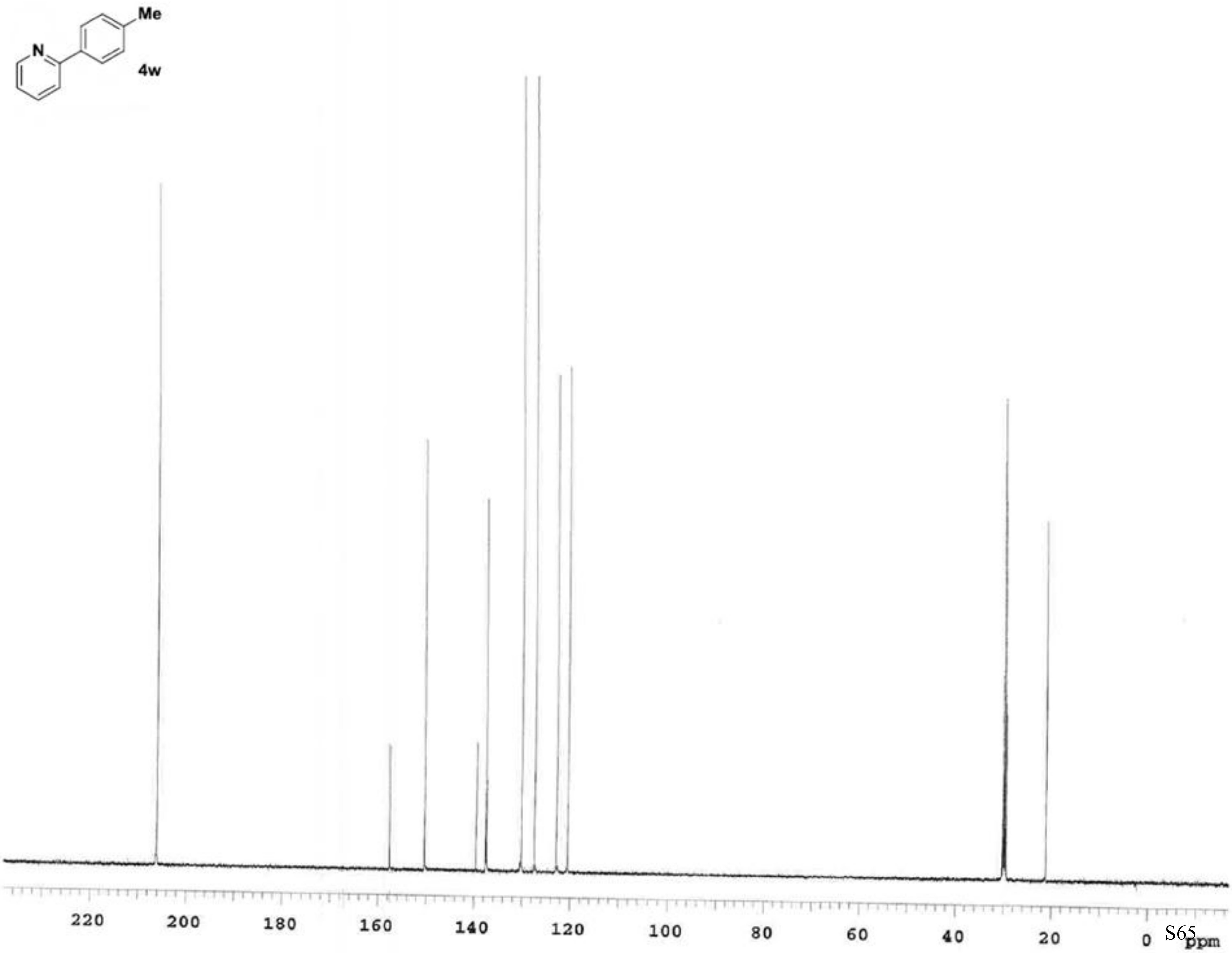
Me
N
4w
220
200
180
160
140
120
100
80
60
40
20
0
ppm

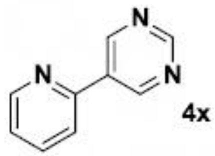

4x

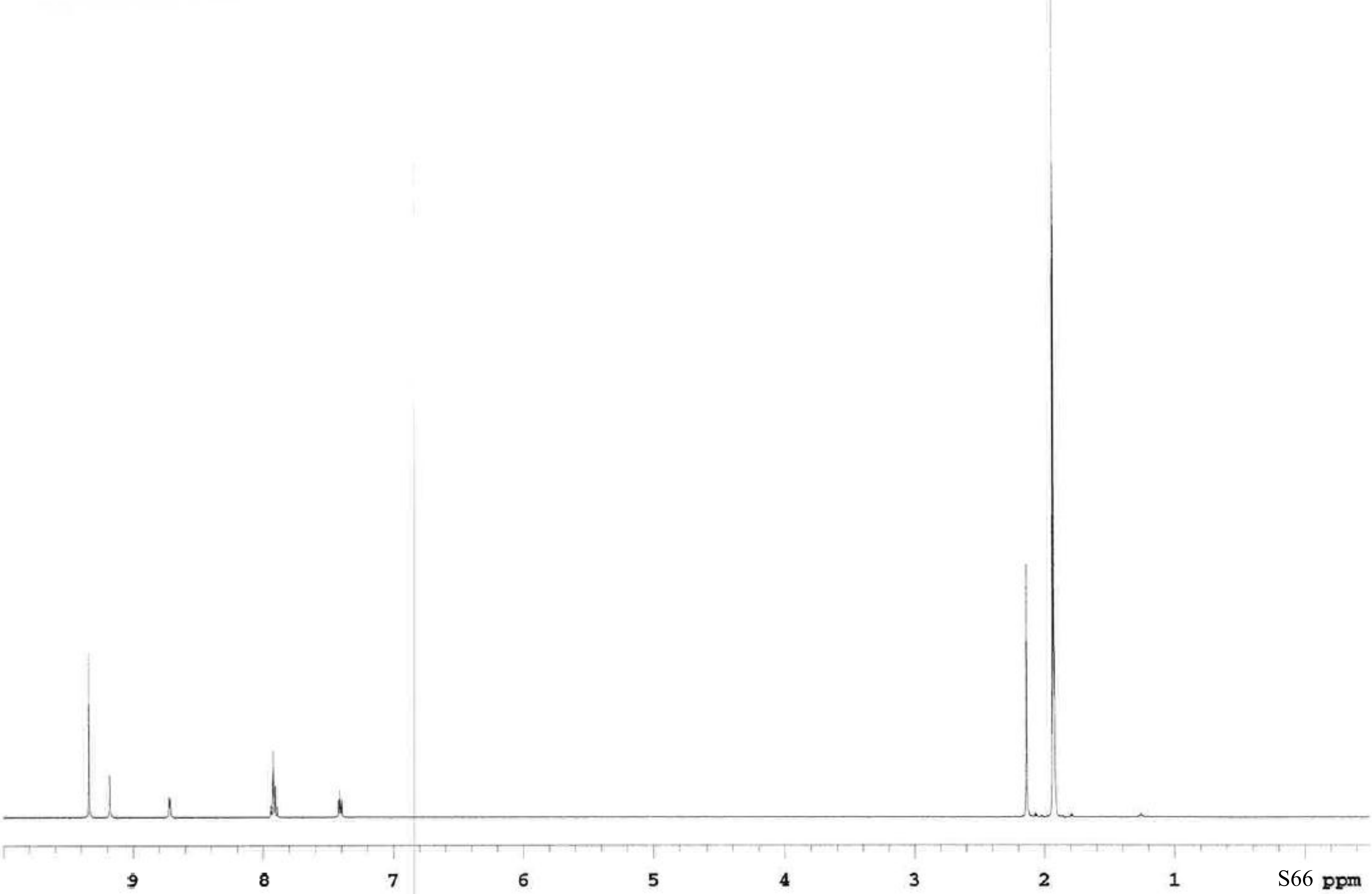

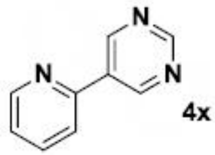

4x

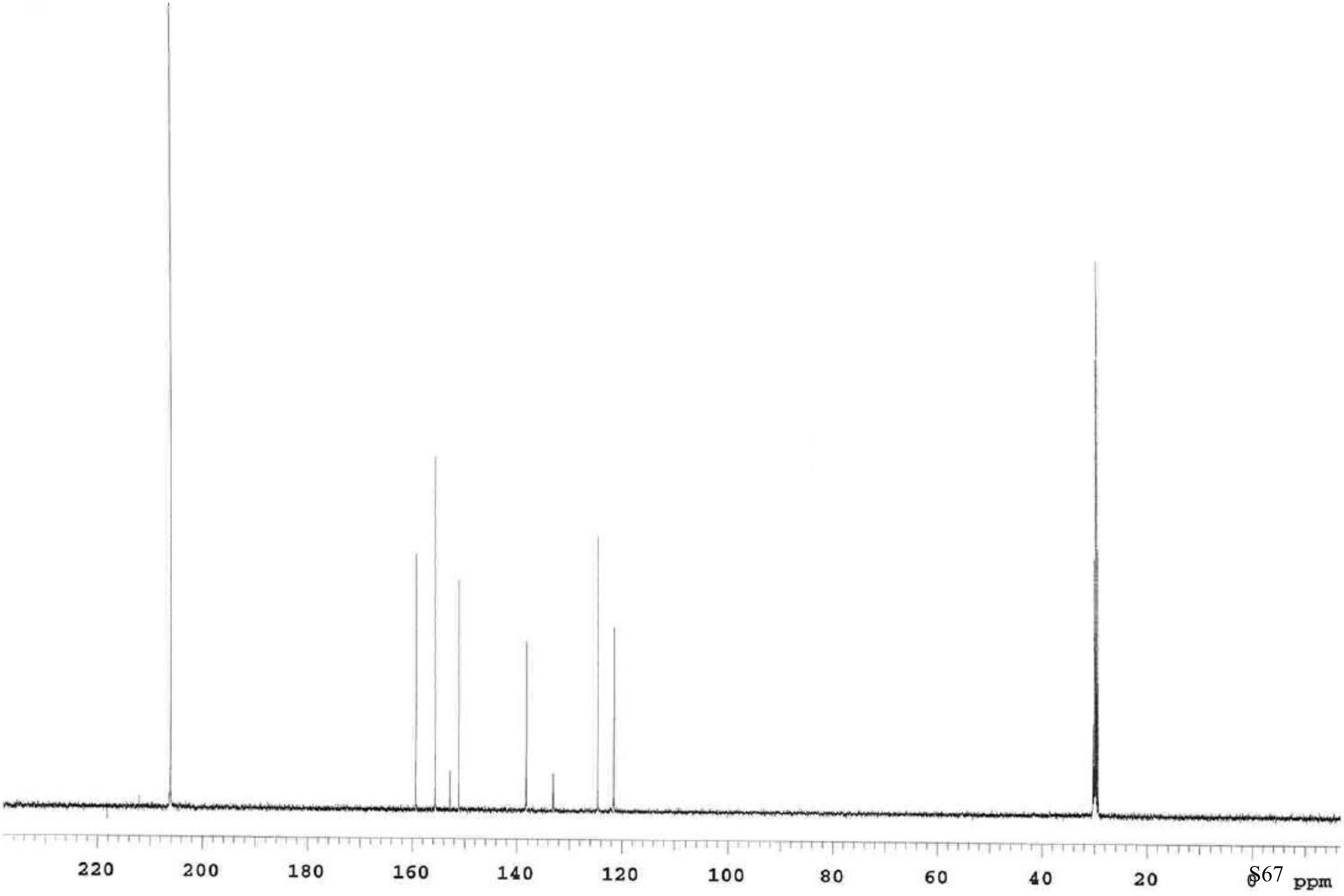

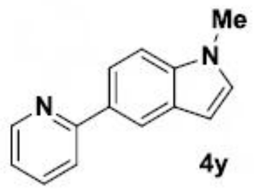

4y

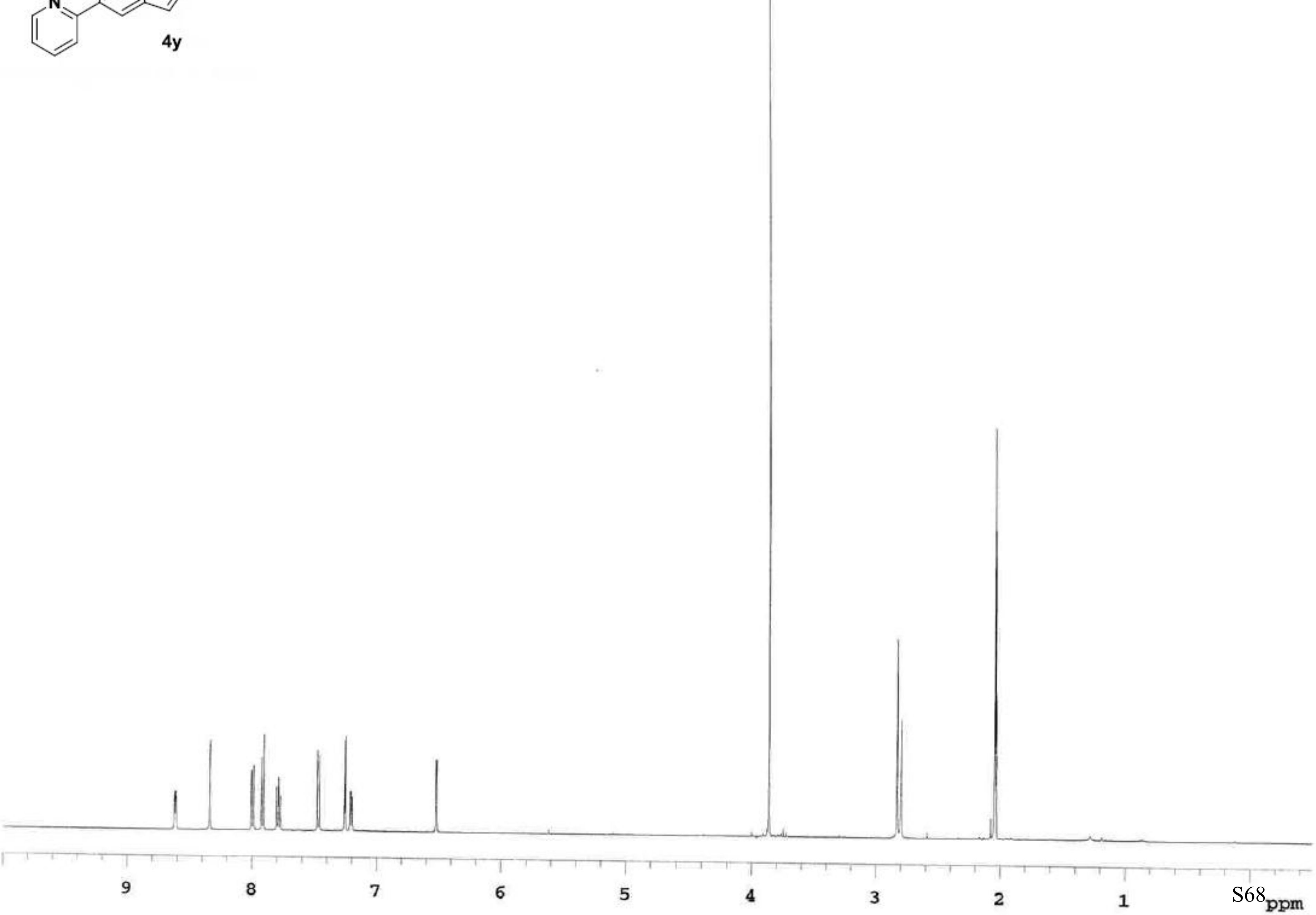

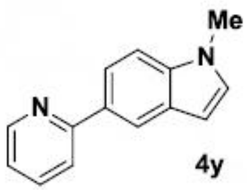

4y

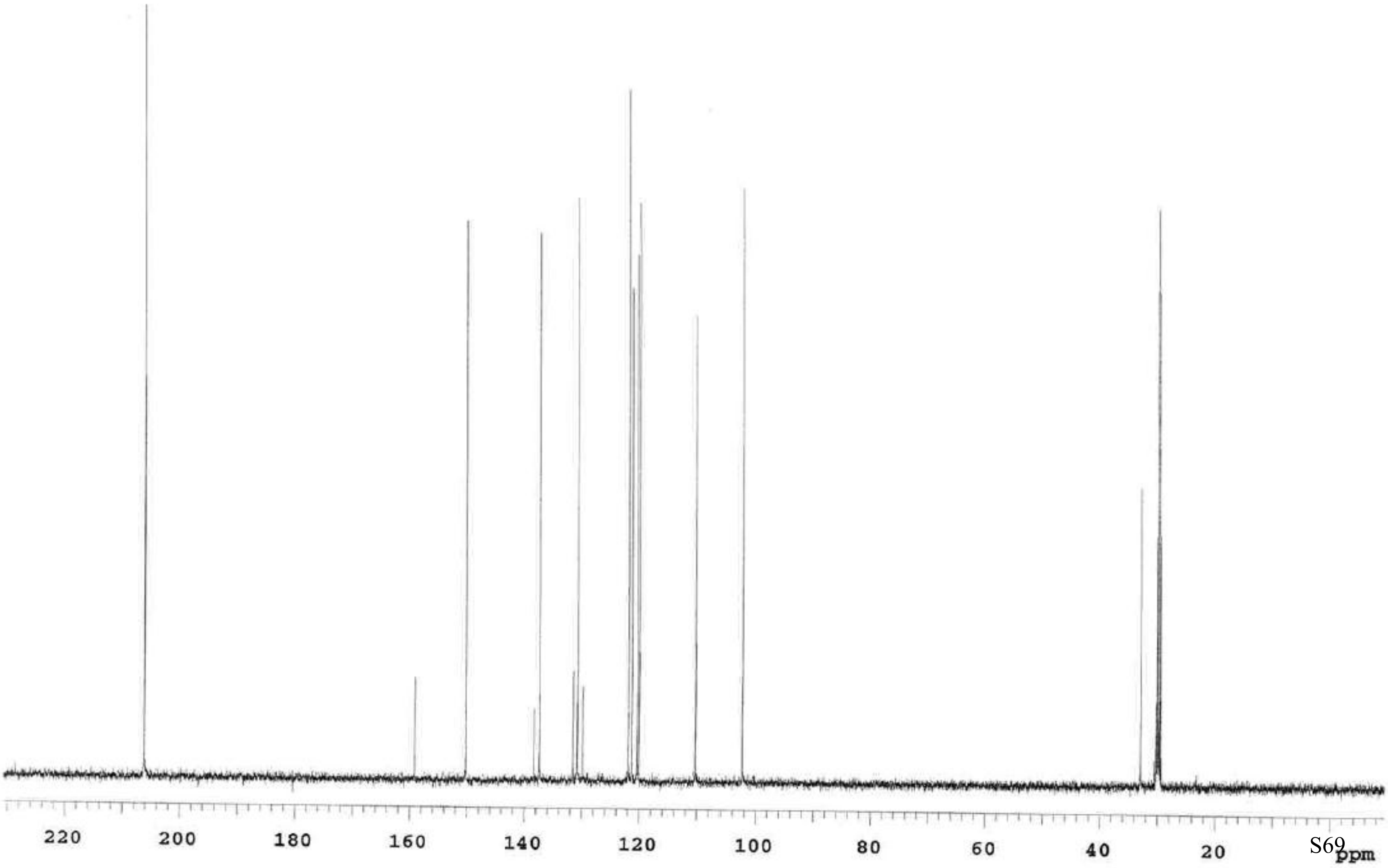

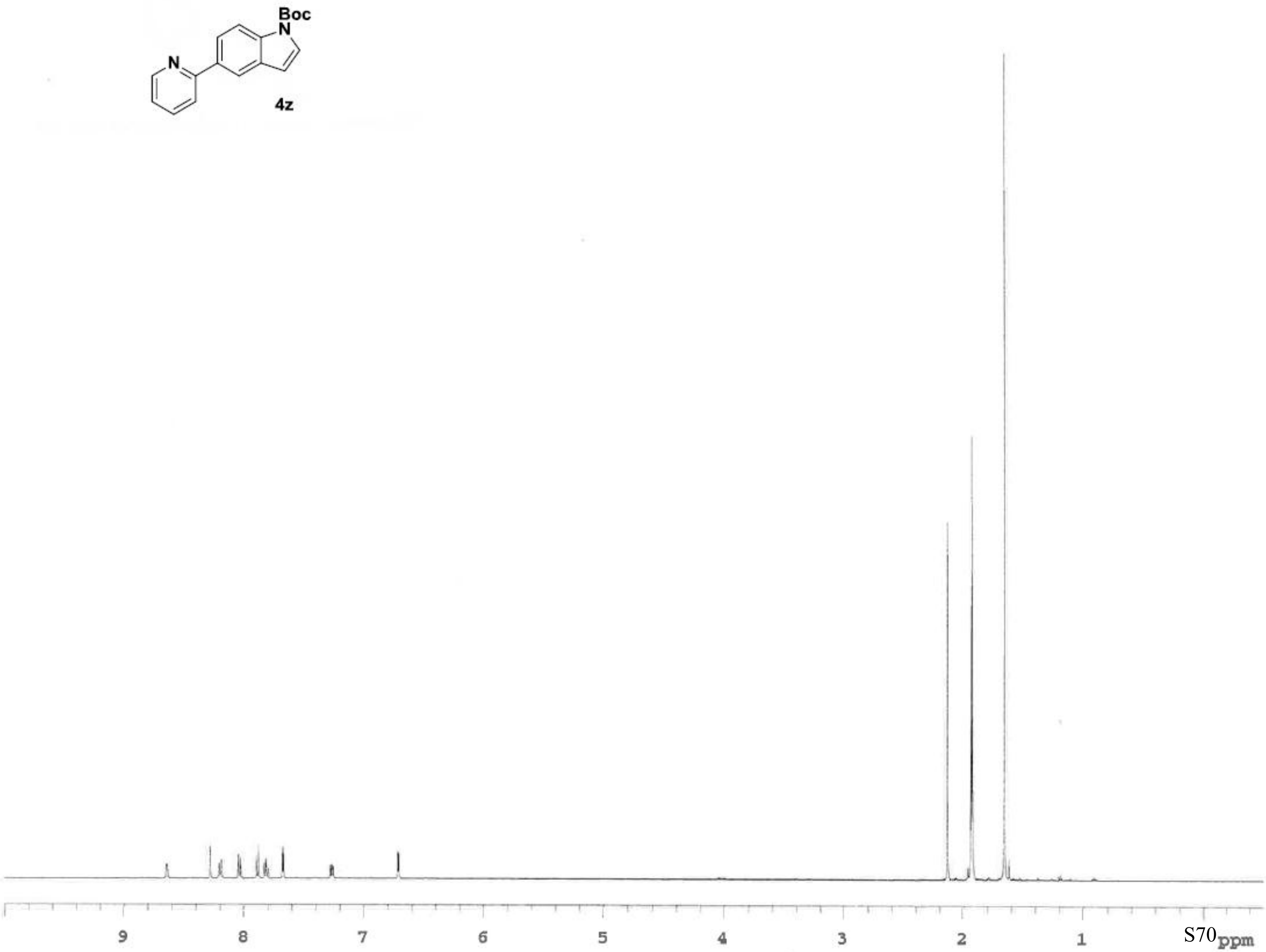
Boc
N
N
4z
9
8
7
6
5
4
3
2
1
ppm

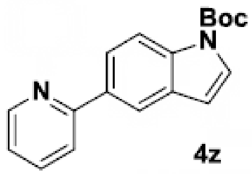

4z

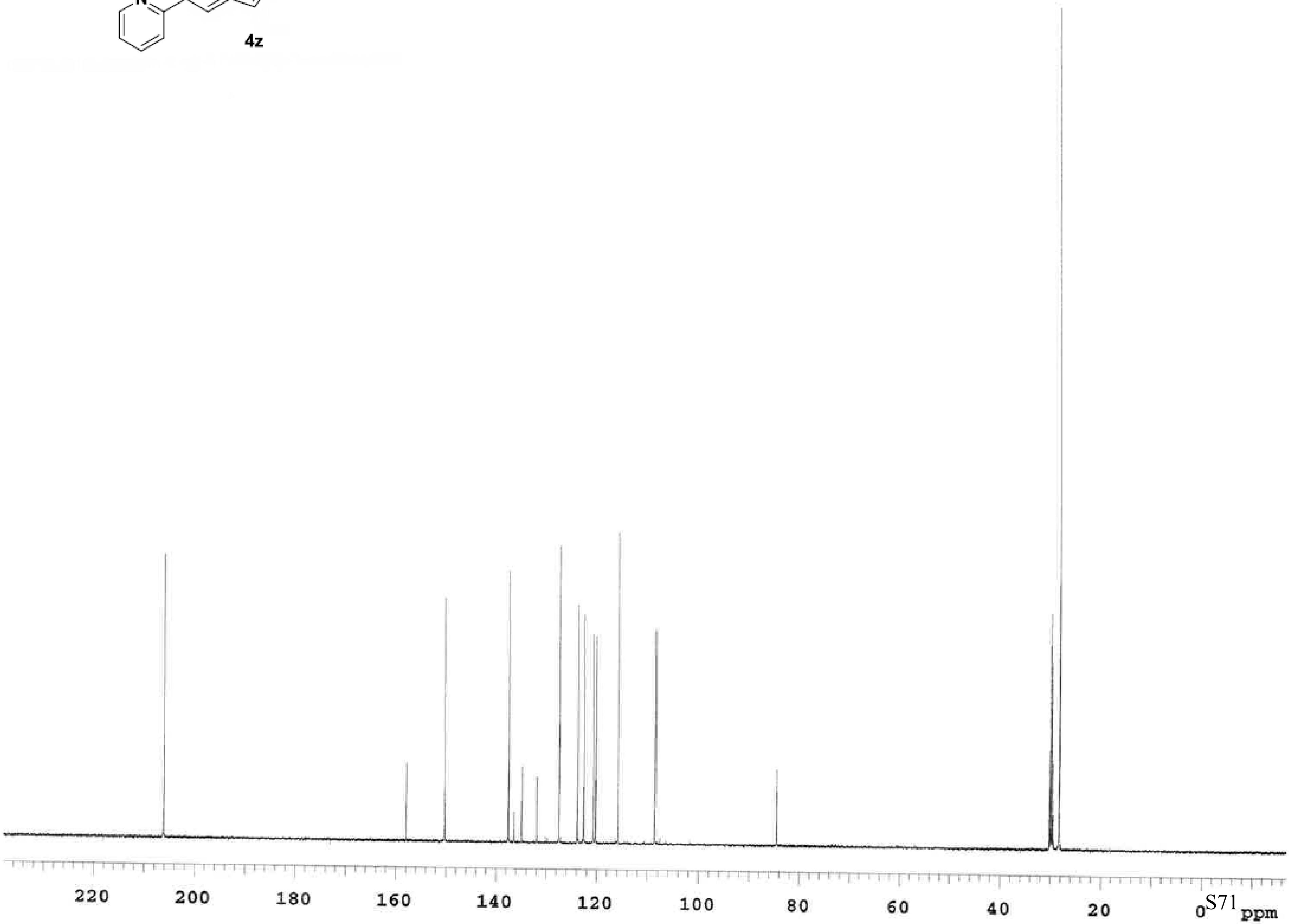

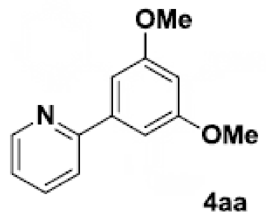

4aa

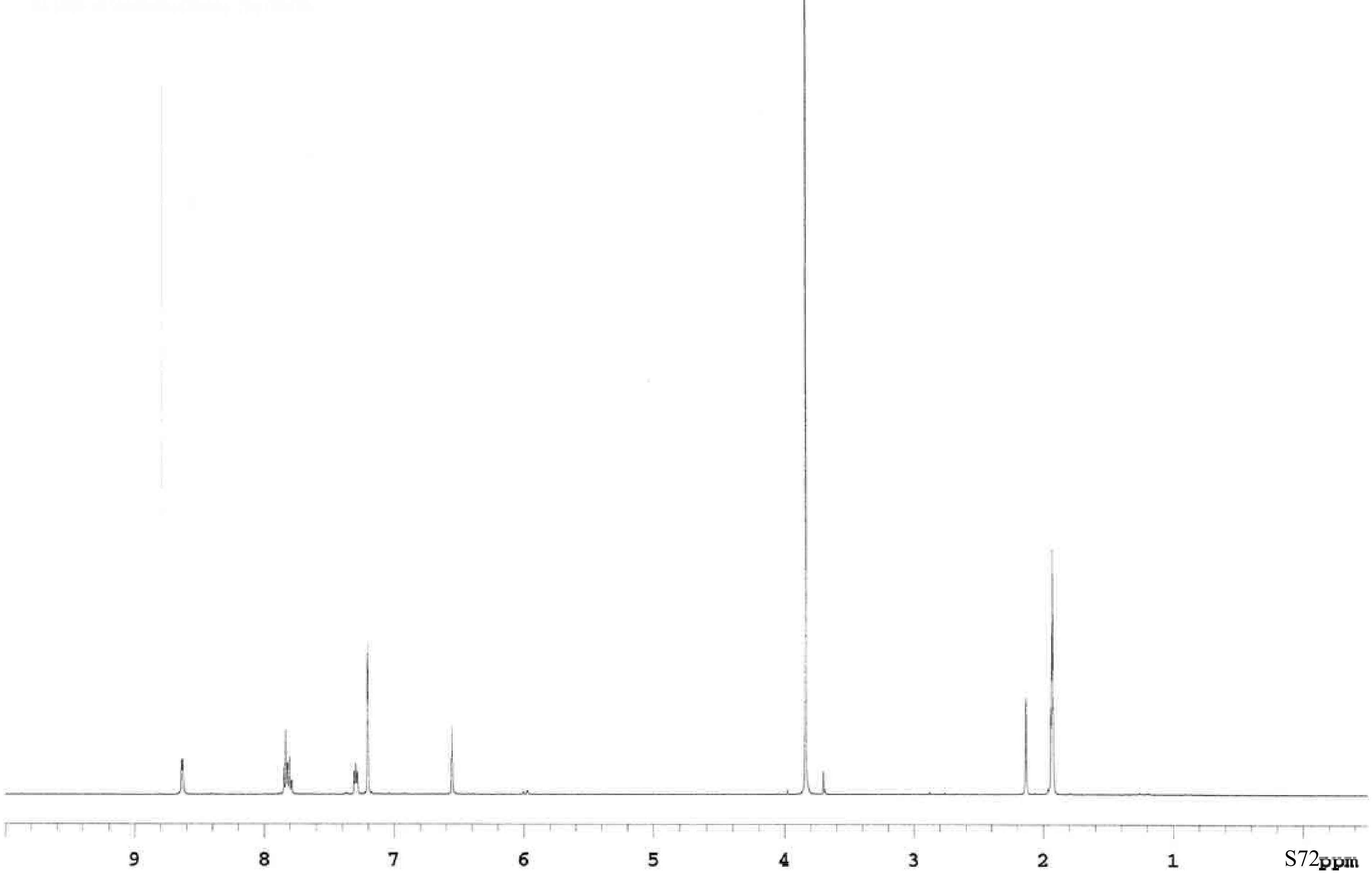

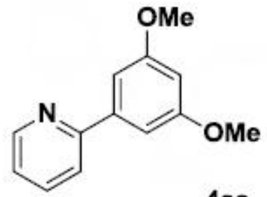

4aa

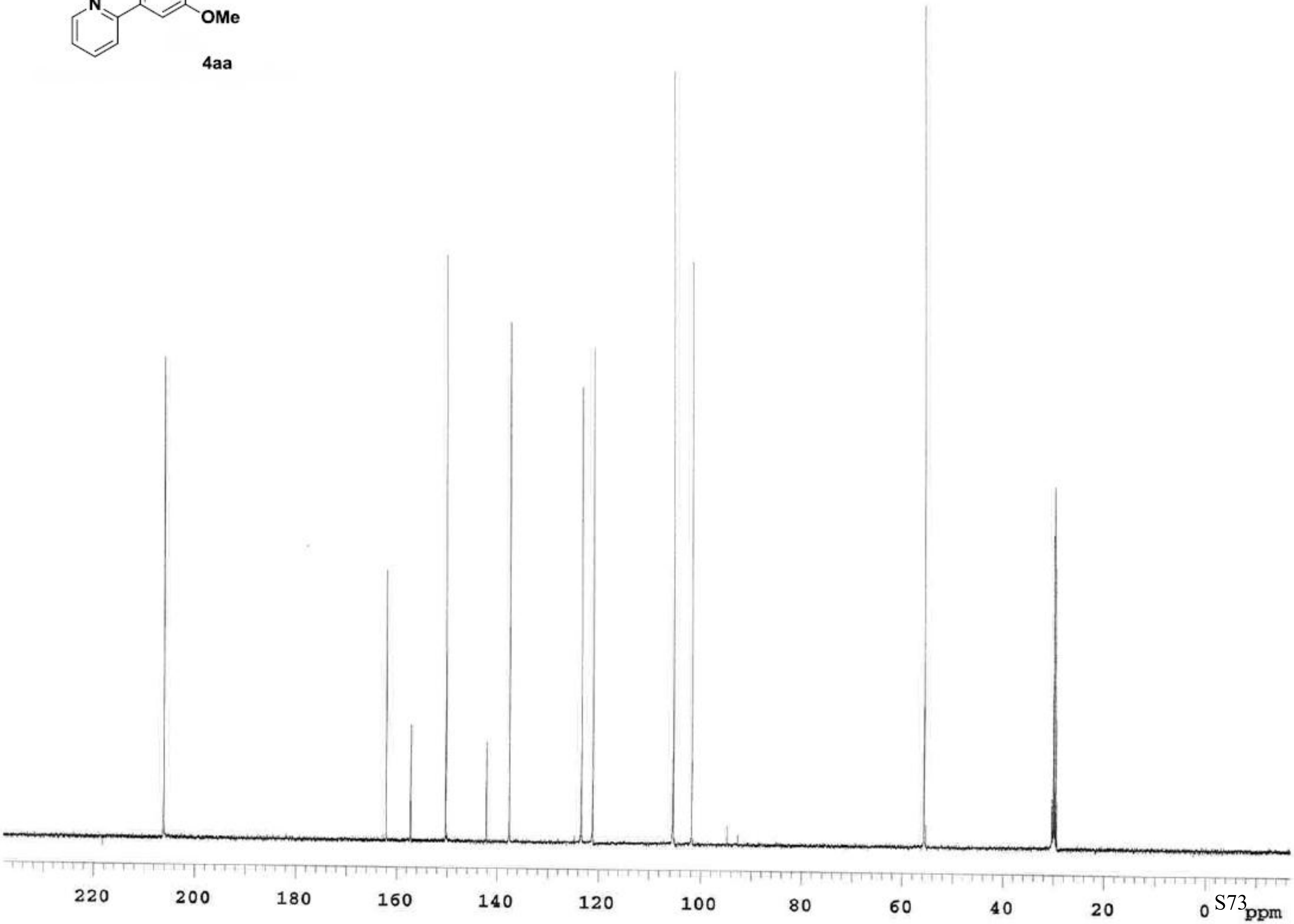

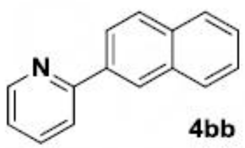

4bb

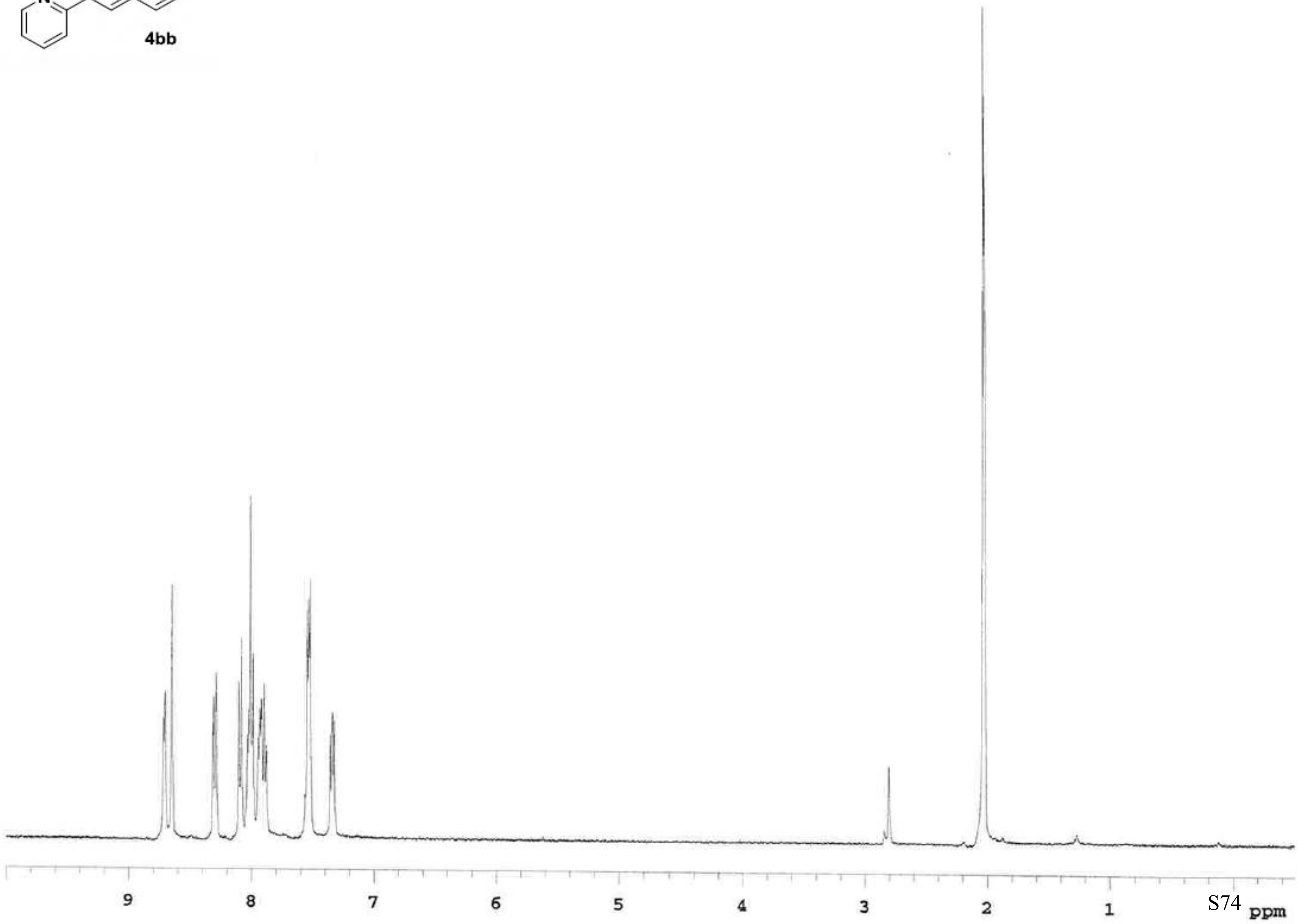

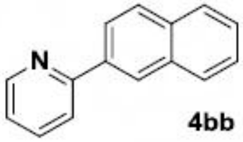

4bb

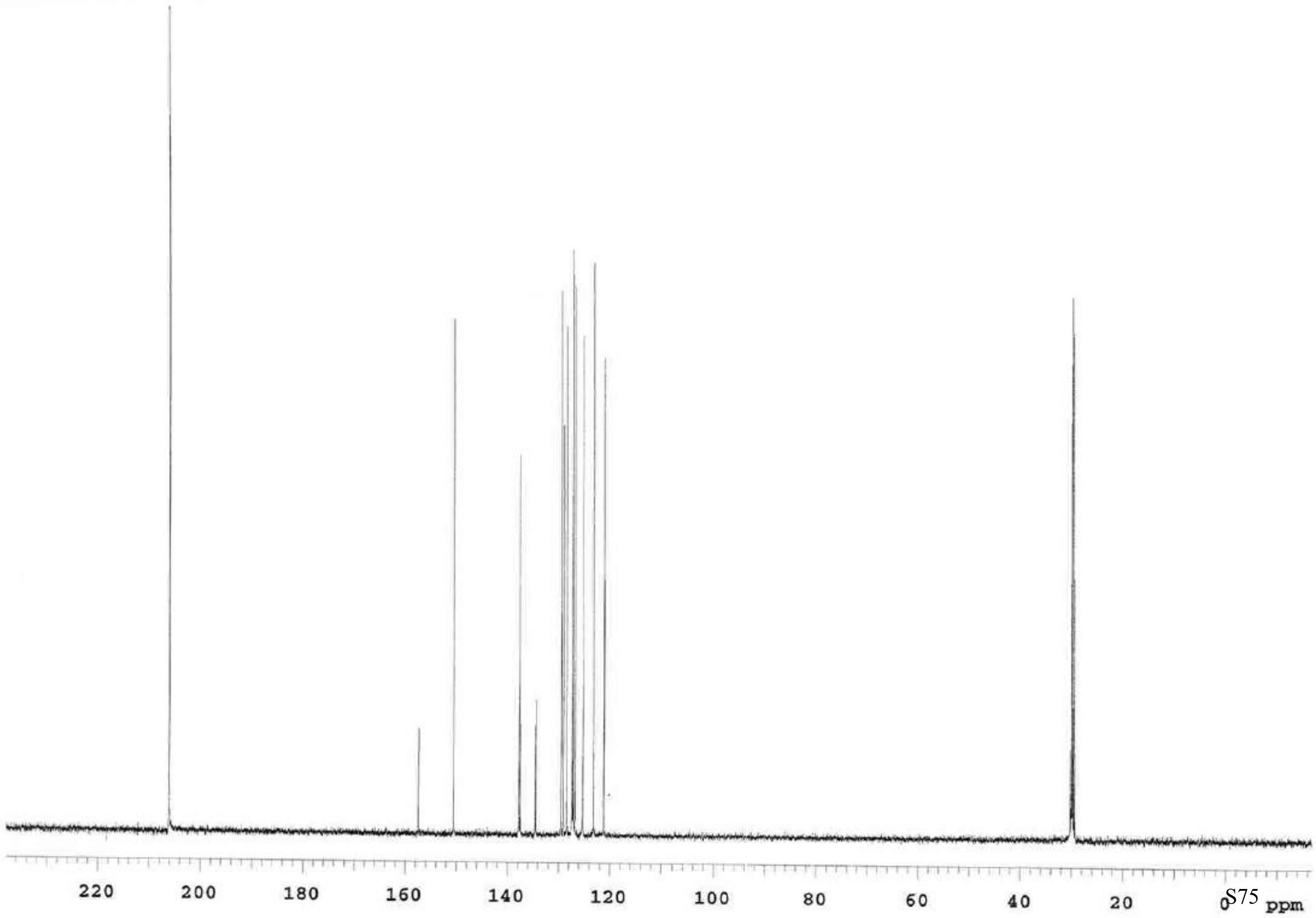

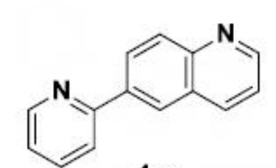

4cc

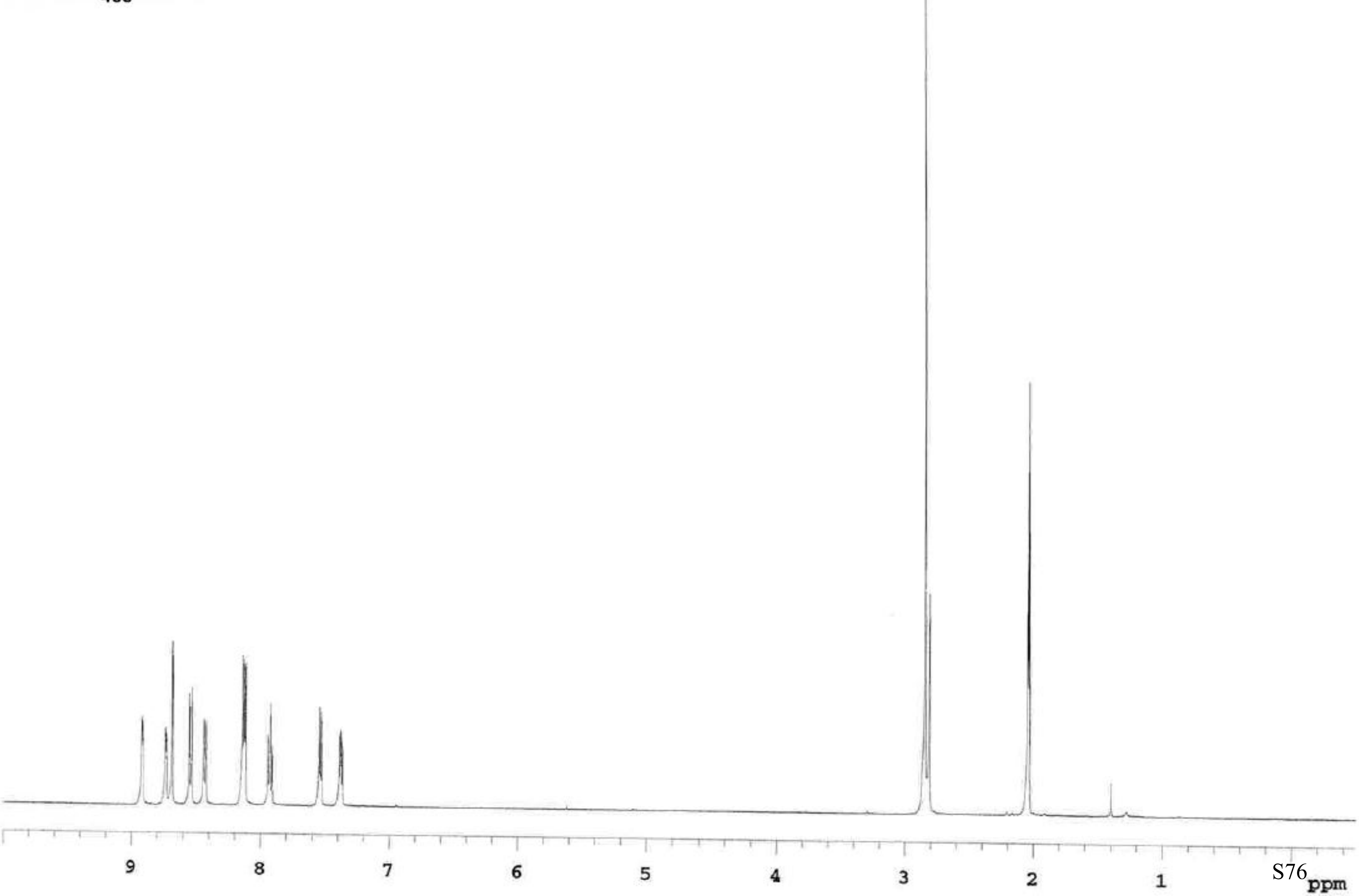

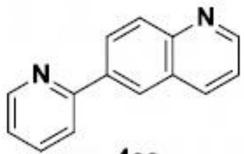

4cc

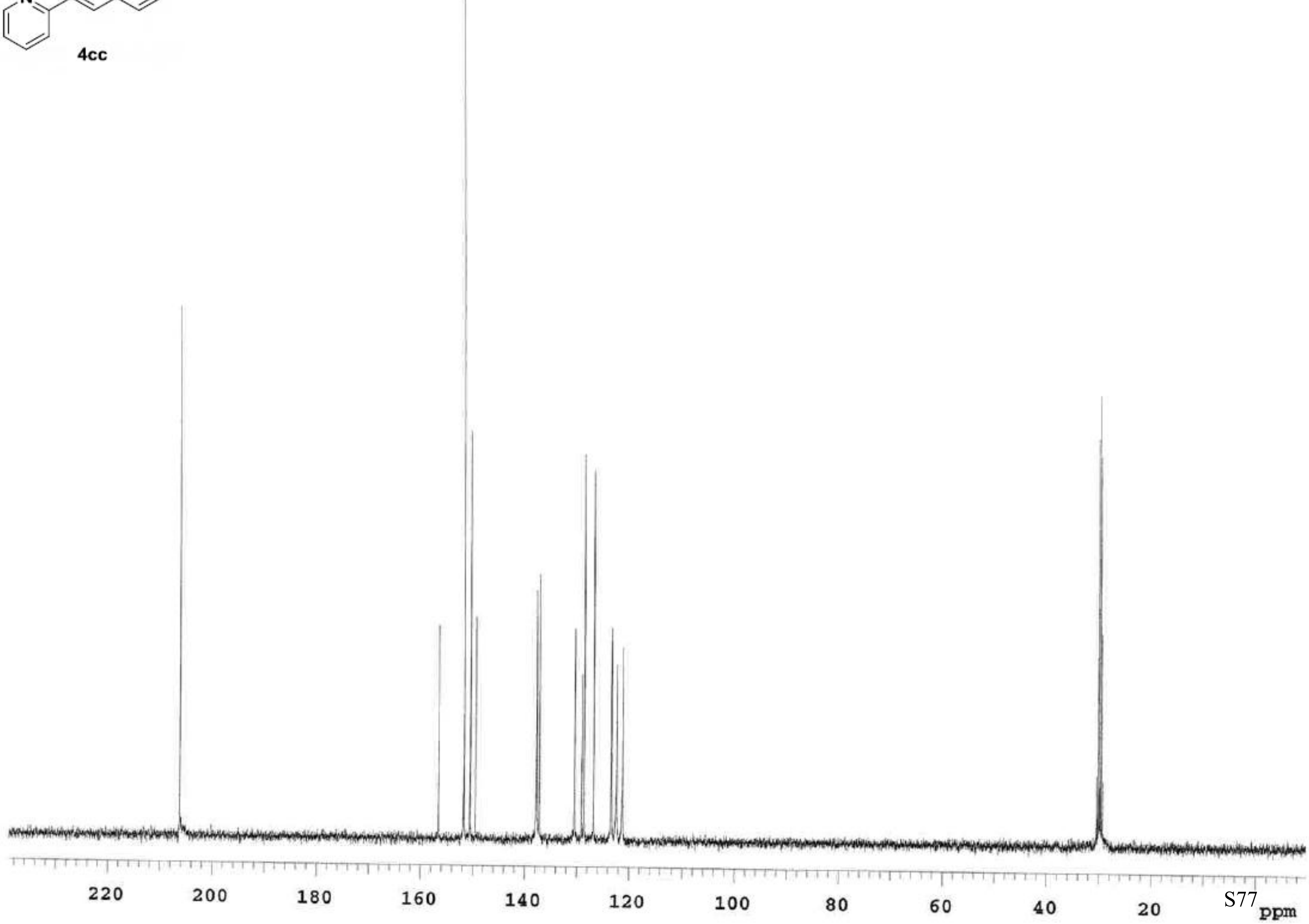

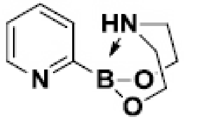

5

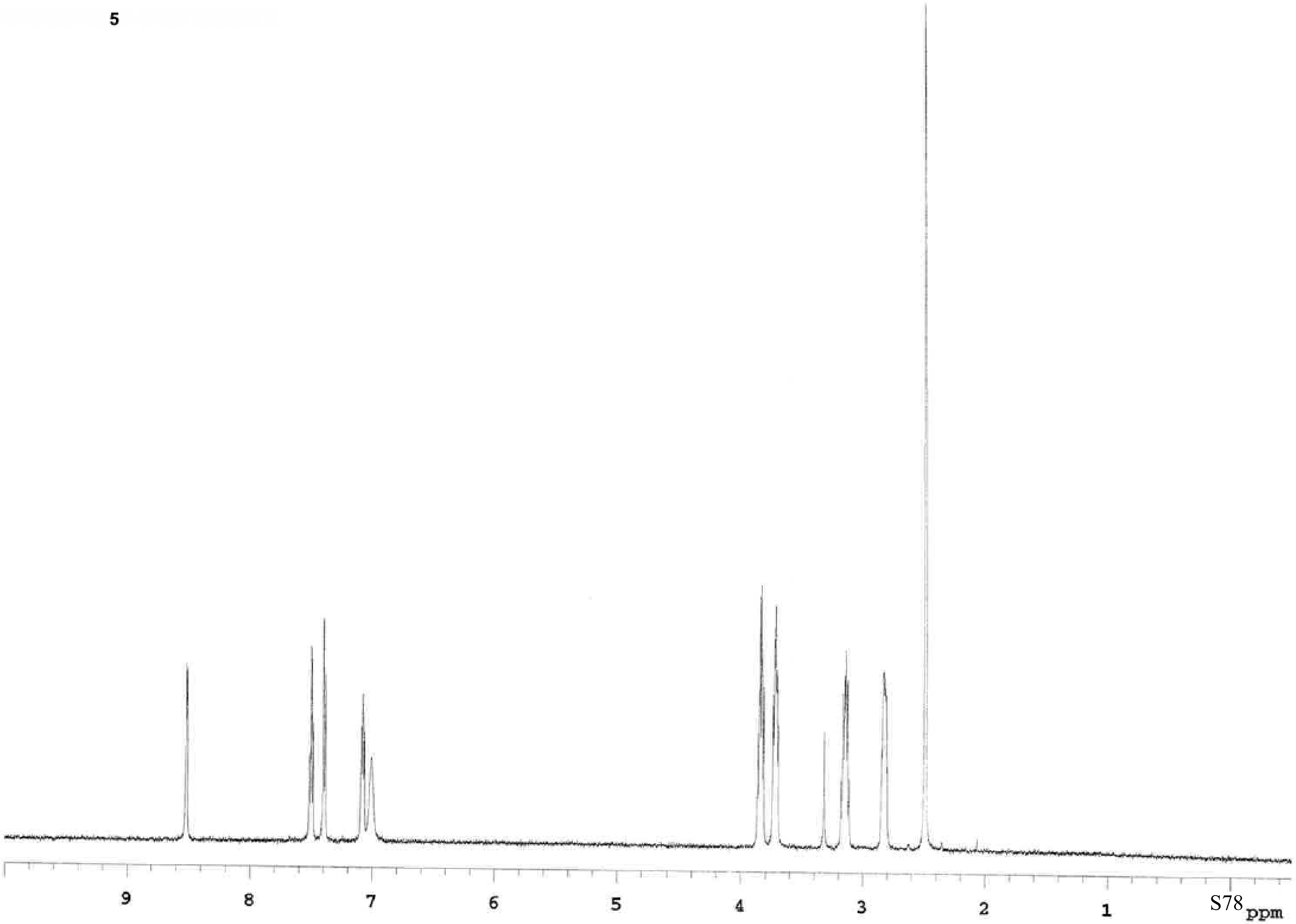

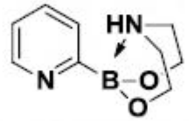

5

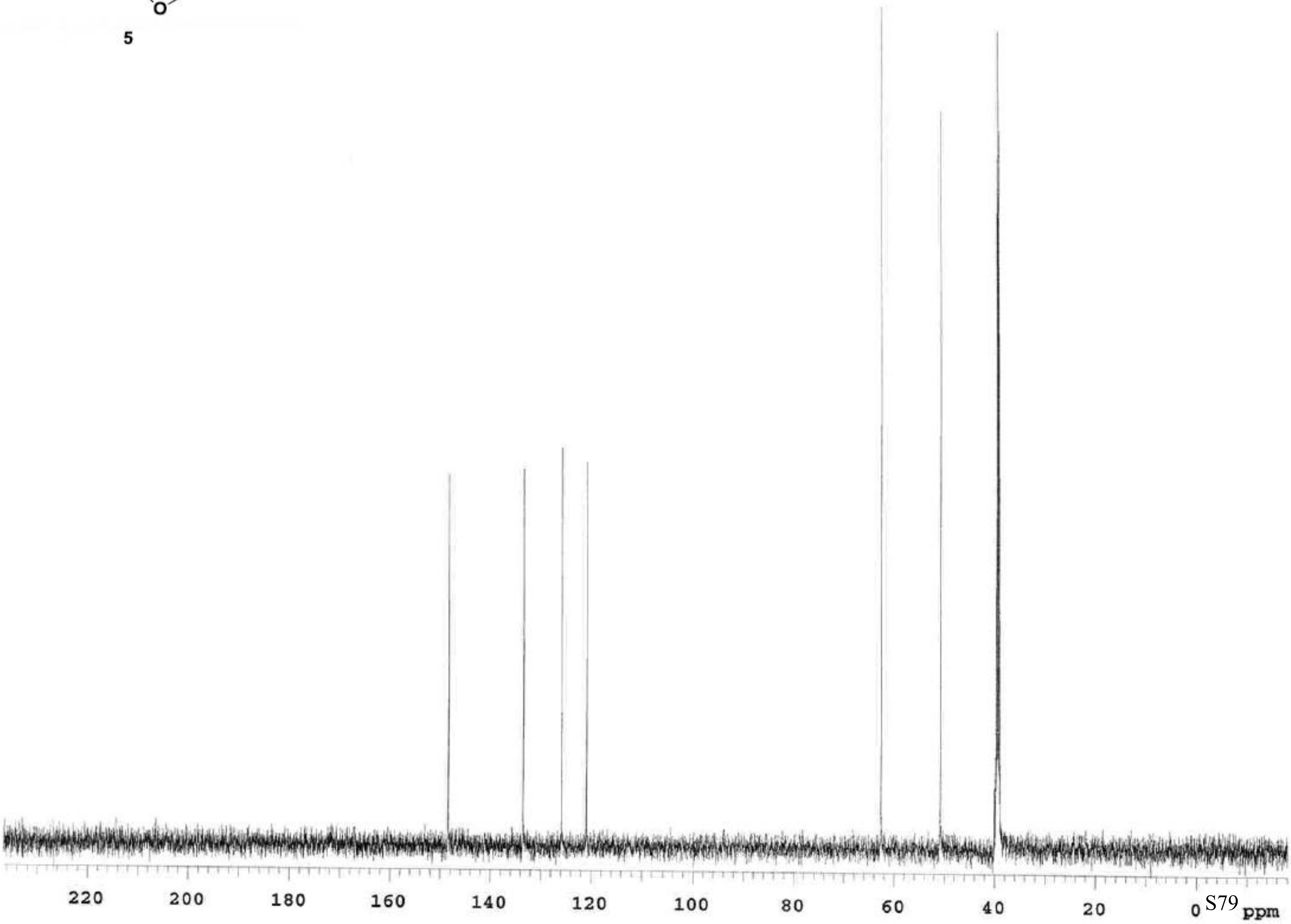

Supplement: Supplementary file 1 [file anie0051-2667-SD1.pdf]
